# Genome-wide identification and evaluation of constitutive promoters in streptomycetes

**DOI:** 10.1186/s12934-015-0351-0

**Published:** 2015-10-29

**Authors:** Shanshan Li, Junyang Wang, Xiao Li, Shouliang Yin, Weishan Wang, Keqian Yang

**Affiliations:** State Key Laboratory of Microbial Resources, Institute of Microbiology, Chinese Academy of Sciences, Beijing, 100101 People’s Republic of China

**Keywords:** Streptomycetes, Constitutive promoter, Transcriptome data, Metabolic engineering, Synthetic biology

## Abstract

**Background:**

Streptomycetes attract a lot of attention in metabolic engineering and synthetic biology because of their well-known ability to produce secondary metabolites. However, the available constitutive promoters are rather limited in this genus.

**Results:**

In this work, constitutive promoters were selected from a pool of promoters whose downstream genes maintained constant expression profiles in various conditions. A total of 941 qualified genes were selected based on systematic analysis of five sets of time-series transcriptome microarray data of *Streptomyces coelicolor* M145 cultivated under different conditions. Then, 166 putative constitutive promoters were selected by following a rational selection workflow containing disturbance analysis, function analysis, genetic loci analysis, and transcript abundance analysis. Further, eight promoters with different strengths were chosen and subjected to experimental validation by green fluorescent protein reporter and real-time reverse-transcription quantitative polymerase chain reaction in *S. coelicolor*, *Streptomyces venezuelae* and *Streptomyces albus*. The eight promoters drove the stable expression of downstream genes in different conditions, implying that the 166 promoters that we identified might be constitutive under the genus *Streptomyces*. Four promoters were used in a plug-and-play platform to control the expression of the cryptic cluster of jadomycin B in *S*. *venezuelae* ISP5230 and resulted in different levels of the production of jadomycin B that corresponded to promoter strength.

**Conclusions:**

This work identified and evaluated a set of constitutive promoters with different strengths in streptomycetes, and it enriched the presently available promoter toolkit in this genus. These promoters should be valuable in current platforms of metabolic engineering and synthetic biology for the activation of cryptic biosynthetic clusters and the optimization of pathways for the biosynthesis of important natural products in *Streptomyces* species.

**Electronic supplementary material:**

The online version of this article (doi:10.1186/s12934-015-0351-0) contains supplementary material, which is available to authorized users.

## Background

Streptomycetes are renowned as a rich source of bioactive natural products of clinical, agricultural, and biotechnological value [1, 2], and they attract large amounts of attention in metabolic engineering and synthetic biology [3]. Promoters play an important role in the aforementioned fields because the fundamental level of transcriptional control takes place at promoter elements that drive gene expression [4, 5]. Therefore, it is crucial to have a powerful promoter toolkit for different purposes of research [6–9].

In streptomycetes, only a limited number of promoters have been described and shown to be functional for the expression of heterologous genes. For example, *ermE*p*, the constitutive promoters of erythromycin resistance gene (*ermE*) of *Streptomyces erythraeus* where the asterisk signifies the presence of a one-base-pair mutation [10]; SF14p, another constitutive promoter isolated form *Streptomyces ghanaensis* phage I19 [11]; *kasO*p*, a strong promoter engineered by removing the binding sites of ScbR and ScbR2 from promoter of *kasO* (*sco*6280) in *Streptomyces coelicolor* [12]; *tipA*p, a thiostrepton-inducible promoter of gene *tipA* from *Streptomyces lividans* 66 [13]; and *nitA*p, an ε-caprolactam-inducible promoter of nitrilase gene (*nitA*) in *Rhodococcus rhodochrous* J1 [14]. Among them, ermE*p is the only acknowledged constitutive promoter. Recently, 10 strong promoters in *Streptomyces albus* were identified in Zhao’s lab, but not all of them were constitutive [15]. Therefore, reliable constitutive promoters are rather limited in streptomycetes.

Constitutive promoters are useful in metabolic engineering and synthetic biology. For instance, constitutive promoters with different strengths are useful for fine-tuning gene expression levels, which can facilitate the pathway optimization of the desired chemicals for higher production [16, 17]. Strong constitutive promoters also can be used to trigger expression of some cryptic clusters, resulting in the discovery of novel natural products [3, 18]. Hence, it is necessary to identify more constitutive promoters in streptomycetes.

Constitutive promoters are usually obtained from the promoters of essential genes whose transcript levels are presumed to be constant [16, 19]. However, for streptomycetes, essential genes are not always constant during the whole life cycle. For example, HrdB (encoded by *hrdB*) is the essential principle sigma factor and is thought to have stable expression in streptomycetes. However, the transcription of *hrdB* is regulated by several factors and its transcript level is not always constant [20–22]. Because *Streptomyces* species have long and complex life cycles, including sophisticated metabolism shifts and morphological differentiation [1], the expression levels of essential genes might be appropriately modulated to adapt to physiological changes. Hence, the traditional approaches to identify constitutive promoters in microbes with short growth periods, such as *Escherichia coli* and *Saccharomyces cerevisiae*, may be not reliable for organisms with complex developmental life cycles like streptomycetes.

With the increasing interest in *Streptomyces* in basic and industrial research, increased transcriptome data are freely available from public databases, especially data from the model organism *Streptomyces coelicolor*, which offers an opportunity to mine reliable constitutive promoters. In this work, based on the systematic analysis of time-series transcriptome data of the model organism *S. coelicolor* cultivated in different conditions, a panel of constitutive promoters with different strengths was identified. These promoters were experimentally evaluated by green fluorescent protein (GFP) reporter and real-time reverse-transcription quantitative polymerase chain reaction (RT-qPCR) in *S. coelicolor* M1146, *Streptomyces venezuelae* WVR2006, and *S. albus* J1074; these promoters appeared to be constitutive in different *Streptomyces* species. These constitutive promoters enriched the promoter toolbox of streptomycetes, which should be of great value for metabolic engineering and synthetic biology in this genus.

## Results

### Selection of genes with constant expression profiles in *S. coelicolor*

**Fig. 1:**
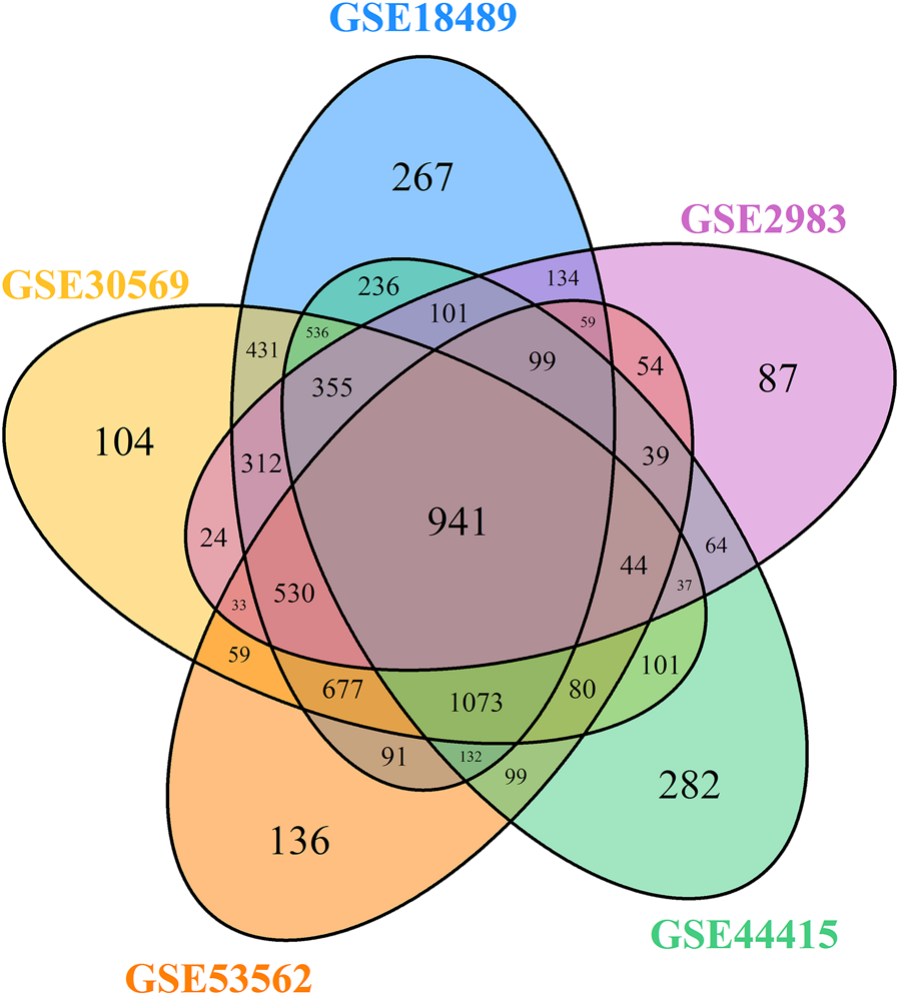
Number of genes with constant expression profiles in *S. coelicolor* M145. The *Venn diagram* indicates the number of stably expressed genes based on the time-series transcriptome of M145 cultivated in different conditions

Constitutive promoters offer relatively constant gene expression profiles. The selection of genes with highly constant expression profiles could allow us to identify constitutive promoters. Therefore, we extracted five sets of time-series transcriptome microarray data of *S. coelicolor* M145 from the National Center for Biotechnology Information Gene Expression Omnibus (NCBI GEO) database: GSE2983, GSE18489, GSE30569, GSE44415, and GSE53562. The five transcriptome datasets were obtained from the growth of M145 in different conditions, such as medium and culture temperature (Additional file 1: Table S1). Global analysis of gene expression profiles in the five datasets showed that there were 2990, 6019, 5375, 4218, and 4145 genes with constant expression profiles in GSE2983, GSE18489, GSE30569, GSE44415, and GSE53562, respectively (Additional file 2: Dataset S1). To obtain genes with stable expression profiles in the five aforementioned conditions, the intersection of the stably expressed genes in the five datasets was determined, and it contained 941 genes (Fig. 1), implying that the promoters of these genes might be constitutive.

### Rational selection of putative constitutive promoters in *S. coelicolor*

**Fig. 2:**
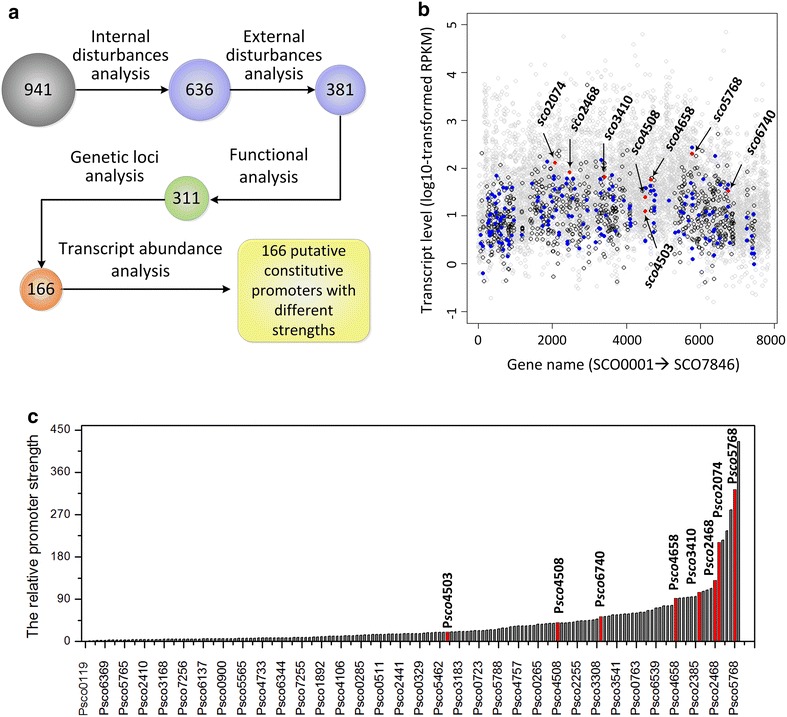
Rational selection of constitutive promoters in *S. coelicolor*. **a** Flow chart of the selection of constitutive promoters. **b** Transcript levels of genes with stable expression profiles after primary identification (941, *black circles*) and rational selection (166, *blue dots*). The *red dots* indicated genes whose promoters were selected for experiment validation. **c** The relative strengths of the 166 putative constitutive promoters based on RNA-Seq. The relative strengths of these promoters were reflected by the reads per kilobase per million mapped reads (RPKM) values of their downstream genes. The strength of the promoter of *sco*0119, which had the lowest RPKM value, was set to one. The *red column* indicates the experimentally tested constitutive promoters

To identify credible constitutive promoters, the 941 genes were further subjected to a series of rational analyses to obtain more reliable genes with stable expression profiles (Fig. 2a). First, internal disturbance analysis was performed to examine whether the expression profiles of the 941 genes remained constant in front of different mutations. Here, the Δ*glnK* and Δ*phoP* mutants of *S. coelicolor* M145 and their corresponding time-series transcriptome microarray datasets GSE30570 and GSE31068 (Additional file 1: Table S1) were used. There were 110 and 195 genes, whose expression profiles were strain-specific in the Δ*glnK* and Δ*phoP* mutants, respectively, and were removed from our analysis (Additional file 3: Dataset S2). Then, external disturbance analysis was conducted on the remaining 636 genes (Additional file 4: Dataset S3). Our previous work demonstrated that the type II polyketide jadomycin B (5 μM) can act as an external antibiotic signal to modulate the behaviors of *S. coelicolor* [23]. Thus, the transcriptome data of *S. coelicolor* M145 and its Δ*scbR*2 mutant treated with 5 μM jadomycin B (GSE53563) was used to eliminate genes whose expression levels were sensitive to external stress. In this step, 179 and 76 genes were discarded in M145 and its Δ*scbR*2 mutant, respectively (Additional file 3: Dataset S2), and the number of the remaining genes was 381 (Additional file 4: Dataset S3).

Functional analysis was carried out to remove genes whose expression profiles were potentially not stable, although they were qualified according to our current conditions. Genes were classified according to Clusters of Orthologous Groups of proteins categories [24]. Here, four genes falling into the category of [Q] (secondary metabolite biosynthesis, transport and catabolism) were excluded (Additional file 3: Dataset S2), because most genes related to secondary metabolites were obviously temporally controlled, which might be ascribed to multiple regulators, as well as complex physiological or environmental signals [23, 25–27]. Another interesting group was the regulators, whose expression profiles are usually condition-dependent and influenced by a highly dynamic complex regulatory network of *S. coelicolor* [28, 29]. In total, 311 genes (Additional file 4: Dataset S3) were kept after removing the 70 genes (Additional file 3: Dataset S2) that were predicted to be regulators.

There are many cases in which genes in the same operon have different expression profiles [30], implying that there was a potential risk to choosing the promoters of operons as the constitutive promoters. Therefore, genetic loci analysis was performed by predicting operons of M145 based on RNA-Seq (see “Methods”) and removing genes involved in these operons to obtain reliable putative constitutive promoters. There were 145 genes in 127 operons (Additional file 3: Dataset S2), and all of them were eliminated, generating 166 genes with stable expression profiles (Additional file 4: Dataset S3).

According to the genome map of *S. coelicolor* A3(2), the approximate positions of the promoters of the 166 genes could be determined (Additional file 5: Table S2). Here, the 500-bp sequences upstream of the translation start sites (ATG or GTG) of the 166 genes were extracted for further experiments. These regions contained the full-length promoters and the native 5′ untranslated regions (5′ UTRs) of the corresponding mRNAs. To preliminarily speculate the promoter strength, gene transcript levels reflected by reads per kilobase per million mapped reads (RPKM) (see "Methods") were analyzed based on RNA-Seq; this analysis revealed that the 166 genes varied in a large scope, which approximately covered the abundances of 90 % of all genes in M145 (Fig. 2b). Therefore, we ranked the relative strengths of 166 constitutive promoters according to the RPKM values, and the relative strengths also spanned a wide spectrum (Fig. 2c).

### Evaluation of the constitutive promoters in *S. coelicolor*

To examine whether the putative constitutive promoters obtained by systematic analyses were reliable, eight promoters with different levels of strength were selected for experimental tests. The *gfp* reporter gene without promoter was used as a control, and the same reporter controlled by the widely used constitutive promoter ermE*p was used as a reference. The eight promoters were evaluated by determining the fluorescence intensity of GFP and the relative mRNA level of *gfp* in *S. coelicolor* M1146. This strain was chosen because it is unable to produce actinorhodin (Act), prodiginines (Red), calcium-dependent antibiotic (CDA) and the type I yellow *coelicolor* polyketide (yCPK) [31], which can significantly reduce the background interference to the fluorescence of GFP.

**Fig. 3:**
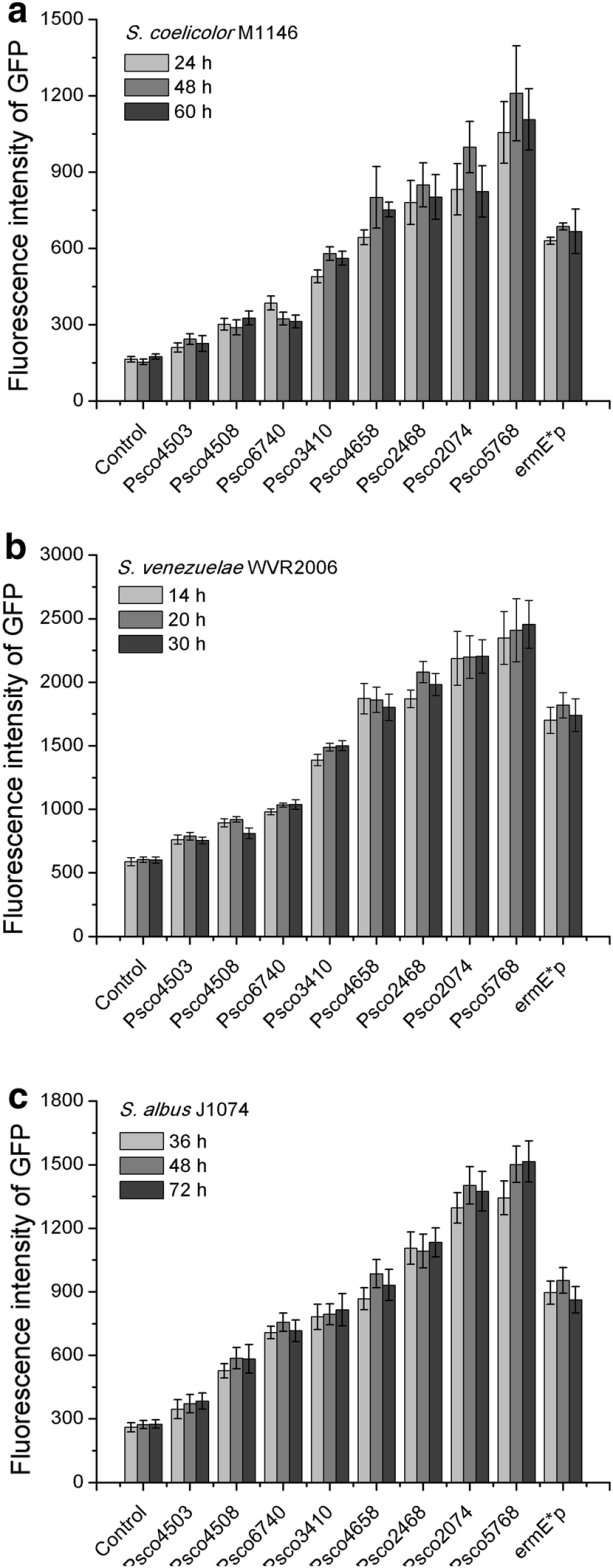
Evaluation of the selected promoters using a GFP reporter. **a** Fluorescence intensities were determined in *S. coelicolor* M1146 derivatives with different promoters. **b** Fluorescence intensities were determined in *S. venezuelae* WVR2006 derivatives with different promoters. **c** Fluorescence intensities were determined in *S. albus* J1074 derivatives with different promoters. “Control” indicates strains with a promoterless GFP reporter. The ermE* promoter was tested in different *Streptomyces* species for comparison. The values are means  ±  standard deviation (SD) from three independent experiments. Data obtained from different growth stages of each strain were not statistically significant

**Fig. 4:**
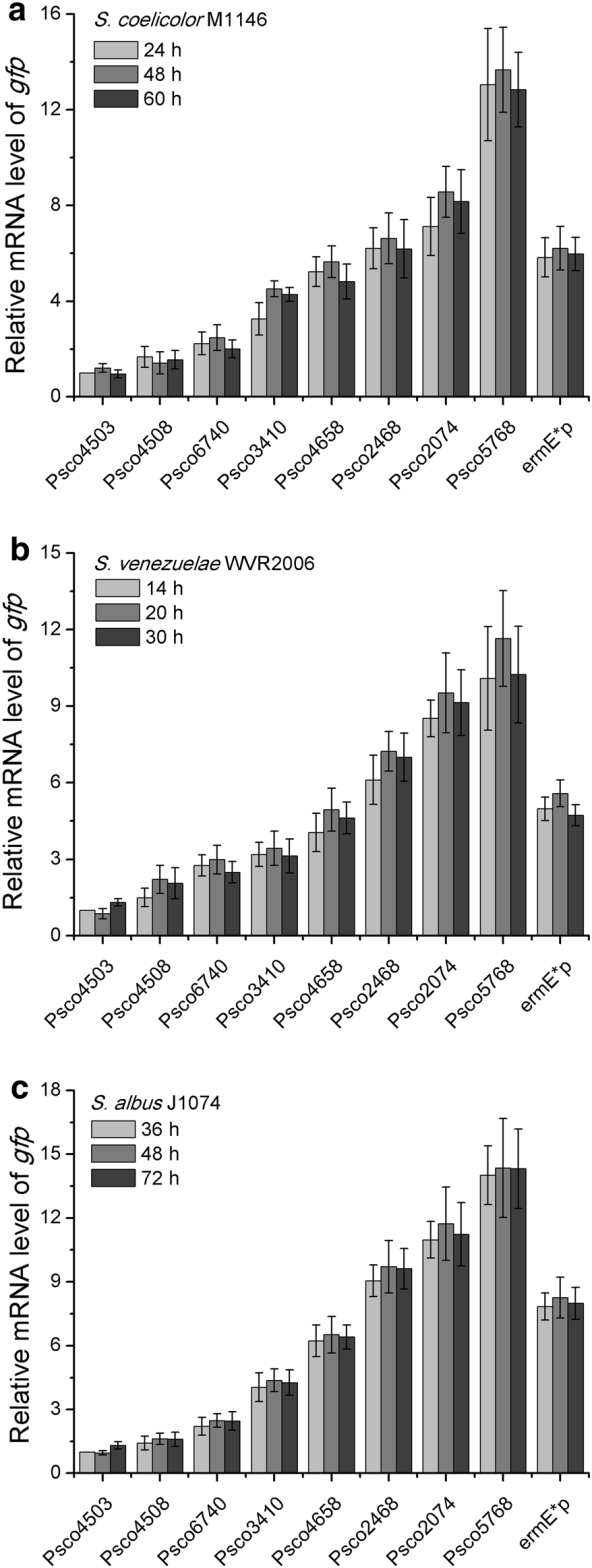
Real-time RT-qPCR assays of the selected promoters. **a** The relative mRNA of *gfp* controlled by different promoters in *S. coelicolor* M1146. **b** The relative mRNA of *gfp* controlled by different promoters in *S. venezuelae* WVR2006. **c** The relative mRNA of *gfp* controlled by different promoters in *S. albus* J1074. The values are means  ±  SD from three independent experiments. Data obtained from different growth stages of each strain were not statistically significant

**Fig. 5:**
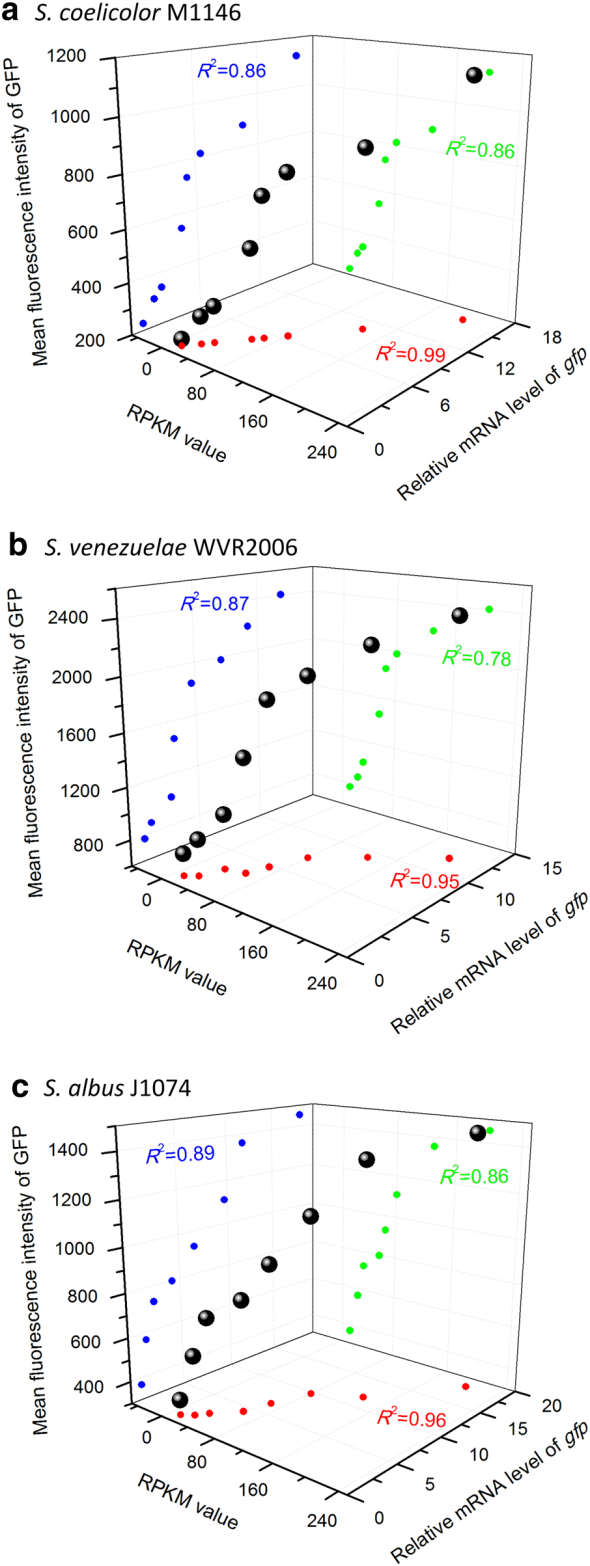
Correlations among data related to promoter strengths obtained by GFP reporter, real-time RT-qPCR, and RNA-Seq. **a** The positive linear relationships of data obtained in *S. coelicolor* M1146 derivatives. **b** The positive linear relationships of data obtained in *S. venezuelae* WVR2006 derivatives. **c** The positive linear relationships of data obtained in *S. albus* J1074 derivatives. *Black dots* in the three-dimensional spaces indicate the overall relationship of promoter strengths reflected by different approaches. The *red*, *green*, and *blue dots* scattered in the three *different planes* indicate the relationships of promoter strengths measured by any two approaches among the three approaches. For each promoter, the data were the averages of strengths generated by three different approaches. *R* ^2^ was the linear regression coefficient

Samples for experiments were harvested at the exponential phase (24 h), transitional phase (48 h), and stationary phase (60 h) (Additional file 6: Figure S1A). For each strain, there was no significant difference among the intensity of GFP fluorescence in different growth stages, meaning all of the tested promoters drove constant gene expression profiles (Fig. 3a), which was consistent with the stable transcript levels of *gfp* determined by real-time RT-qPCR (Fig. 4a). The orders of promoter strength reflected by the GFP reporter and real-time RT-qPCR were identical to the result generated by RNA-Seq (RPKM value). Moreover, positive linear relationships (*R*^2^ > 0.85) existed among the RPKM value, relative mRNA level of *gfp* and fluorescence intensity of GFP (Fig. 5a). These observations demonstrate the high consistency of the results of systematic analysis and experiments, and they suggest that the 166 constitutive promoters might be very reliable in *S. coelicolor*.

### Evaluation of the constitutive promoters in other *Streptomyces* species

To examine whether the 166 constitutive promoters were applicable in the genus of *Streptomyces*, the eight selected promoters were further experimentally evaluated in *S. venezuelae* WVR2006 [32] and *S. albus* J1074 [33]. The experimental designs in the two species were the same as those in *S. coelicolor* M1146. Samples were harvested in exponential phase, transitional phase, and stationary phase: 14, 20, and 30 h for *S. venezuelae* WVR2006 and 36, 48, and 72 h for *S. albus* J1074 (Additional file 6: Figure S1B and C). As shown in Fig. 3b, all eight promoters yielded constant expression of GFP at different growth stages in *S. venezuelae* WVR2006, which also agreed with the stable transcript levels of *gfp* by real-time RT-qPCR (Fig. 4b). The orders of promoter strength ranked by GFP fluorescence intensity and relative mRNA level of *gfp* were both in accordance with that sequenced with RPKM values (RNA-Seq), and positive linear correlations (*R*^2^ > 0.78) were also observed among the results generated by the three different approaches (Fig. 5b). As we speculated, similar results were also obtained in *S. albus* J1074 (Figs. 3c, 4c, 5c). The highly consistent results generated in different *Streptomyces* species indicated that the eight chosen promoters, as well as the rest of the identified promoters, might be reliable constitutive promoters in this genus.

### Engineering the production of jadomycin B in *S. venezuelae*

**Fig. 6:**
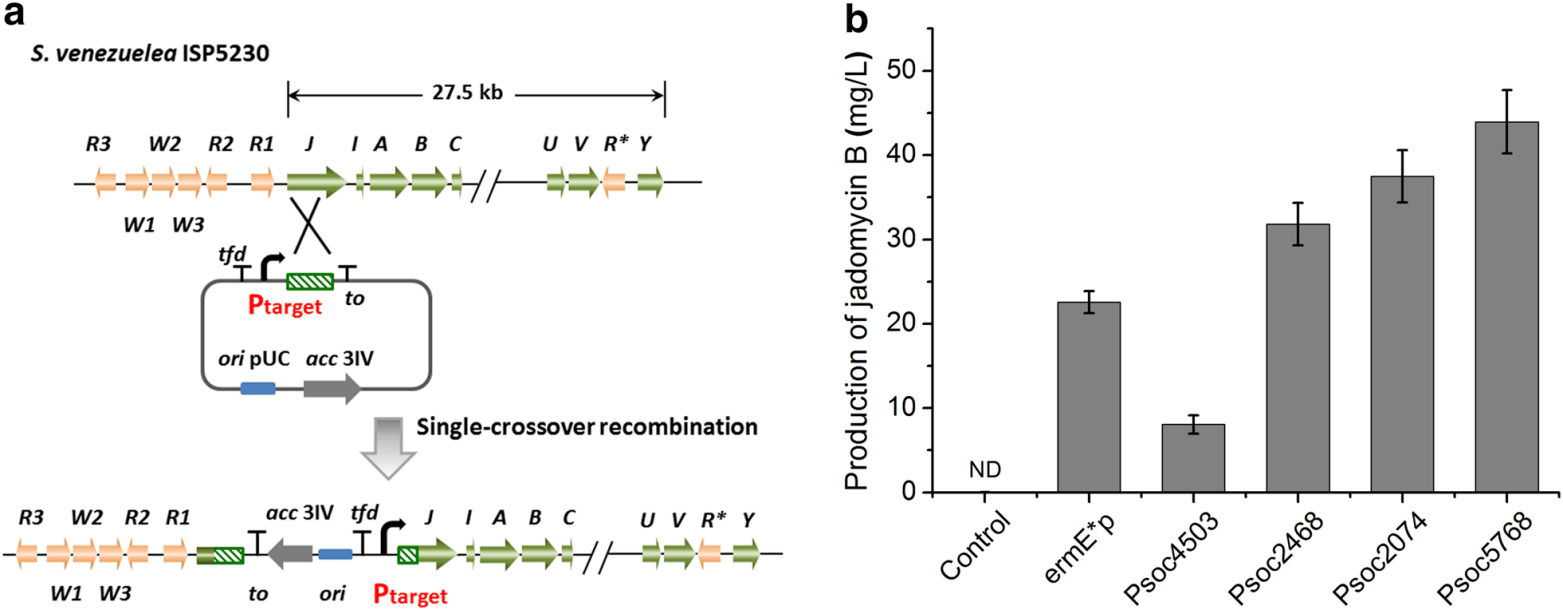
Engineering of *S. venezuelae* ISP5230 using constitutive promoters with different strengths to produce jadomycin B. **a** Diagram for the construction of *S. venezuelae* ISP5230 derivatives with different constitutive promoters by single-crossover recombination. **b** Production of jadomycin B driven by different promoters. “Control” indicates that there was no promoter upstream of *jadJ*. “ND” means that jadomycin B was not detected. The values are means  ±  SD from three independent experiments

To demonstrate an application of the above-evaluated constitutive promoters, they were used to control the production of jadomycin B, which is a type II polyketide antibiotic and is synthesized by a cryptic cluster in *S. venezuelae* ISP5230 [34]. As all 22 structural genes (from *jadJ* to *jadV*) in *jad* cluster are co-transcribed [35], we placed heterologous promoters upstream of *jadJ* in this work to drive the whole jadomycin biosynthetic cluster (Fig. 6a). Among the eight evaluated constitutive promoters, P*sco*4503 had the lowest promoter strength, and P*sco*2468, P*sco*2074, and P*sco*5768 that were stronger than ermE*p were selected. Controlled by the strongest constitutive promoter P*sco*5768, the production of jadomycin B reached 44 mg/L, which was approximately twofold of that controlled by ermE*p. Meanwhile, there was a 5.5-fold range of production generated by the strongest (P*sco*5768) and weakest (P*sco*4503) constitutive promoter (Fig. 6b). These results suggested that the 166 constitutive promoters identified in this work might have a greater range of promoter strengths, which might fulfill the requirements for fine-tuning gene expression in metabolic engineering and synthetic biology in the genus *Streptomyces*.

## Discussion

Constitutive promoters are crucial in metabolic engineering and synthetic biology. In general, constitutive promoters should maintain constant expression that is independent of internal and external variations, such as life cycle and culture conditions. The lack of available constitutive promoters in streptomycetes urges us to enrich this toolbox for *Streptomyces* species.

Recently, researchers have mined constitutive promoters from genome-scale data of *S. albus*, and four of 10 tested promoters were confirmed to be constitutive [15]. The relatively high false-positive rate in the selection of constitutive promoters might be ascribed to the limitations of the transcriptome data, which were obtained from growth in two target culturing conditions and at two defined time points and were inadequate to mimic the profiles of gene transcripts in various conditions. Therefore, we used eight sets of time-series transcriptome microarray data, including five datasets obtained from growth in different media and three datasets obtained from growth in different disturbances, to identify reliable genes with stable expression profiles in various growth stages and culture conditions. Consequently, all of the eight selected promoters from the 166 identified promoters proved to be constitutive in different conditions (Figs. 3, 4, 5), implying that the 166 promoters that we identified are likely to be constitutive in the genus *Streptomyces*. Therefore, our strategy to identify of constitutive promoters in this work was reliable.

The 500-bp sequences upstream of the translation start site of the 166 genes were cloned for promoter evaluation. This region contained the native 5′ UTR of an mRNA and the full-length promoter [15] to ensure that the putative constitutive promoters functioned well. It was also necessary to use the native 5′ UTR to enable the comparison of promoter strengths in diverse backgrounds because the 5′ UTR plays an important role in maintaining the stability of mRNA in prokaryotes [36, 37]. As suspected, the relative promoter strengths generated by the GFP reporter, real-time RT-qPCR, and RNA-Seq were consistent in different tested *Streptomyces* species (Fig. 5). These results suggested that the length of the promoters that we selected might ensure the constant expression of the downstream genes in *Streptomyces* with diverse genetic backgrounds, and the relative strengths of these constitutive promoters were comparable to those in *S. coelicolor* M145.

Among the eight tested constitutive promoters, only three of them possessed higher strength than ermE*p. One reason for this was that we primarily focused on the selection of reliable constitutive promoters based on genes with highly constant expression profiles, while promoters that possessed a high strength within a narrow time window were filtered by the strict cutoff principles. When necessary, stronger constitutive promoters could also be obtained by further promoter engineering [4]. Actually, the 166 constitutive promoters that we identified possessed a wide spectrum of promoter strengths, which should be a valuable resource to meet various requirements of metabolic engineering and synthetic biology in the genus *Streptomyces*.

## Conclusions

In summary, by taking advantage of transcriptome data of the model organism *S. coelicolor*, we were able to select regions of the 166 constitutive promoters by following a strict and rational workflow. Eight of the promoters were experimentally evaluated in three different *Streptomyces* species, and the high consistencies among the data obtained by different approaches implied that the 166 promoters might be reliable constitutive promoters in this genus. Four of the evaluated promoters were used to trigger the cryptic cluster of jadomycin B, and the production of jadomycin B was positively correlated with the promoter strength, suggesting that these constitutive promoters with a wide range of strength might be valuable in metabolic engineering and synthetic biology. Moreover, the systematic and rational strategy employed in this work could be used as a reference for constitutive promoter mining in other organisms.

## Methods

### Bacterial strains and growth conditions

Plasmids and strains used in this work are listed in Table 1. All plasmids were propagated in *E. coli* JM109 (Novagen) cultured in Luria–Bertani (LB) broth with 25 μg/mL apramycin at 37 °C. *E. coli* ET12567/pUZ8002 for conjugation was grown in LB at 37 °C supplemented with antibiotics (chloramphenicol, 25 μg/mL; kanamycin, 25 μg/mL). For spore preparations, *S. coelicolor* M145 [2], M1146 [31], *S. albus* J1074 [33] and their derivatives were maintained on mannitol-soya flour (MS) agar plates, while *S. venezuelae* ISP5230 [38], *S. venezuelae* WVR2006 [32], and their derivatives were grown on maltose-yeast extract-malt extract (MYM) agar plates. The conjugation of all *Streptomyces* strains were implemented on MS agar plates with nalidixic acid (25 μg/mL) and apramycin (25 μg/mL). For fermentations, the derivatives of *S. coelicolor* M1146, *S. venezuelae* WVR2006 and *S. albus* J1074 were shaken at 250 rpm in the liquid supplemented minimal medium (SMM), MYM, and tryptic soy broth (TSB), respectively. All cultivations were carried out at 28 °C.

**Table 1:** Strains and plasmids used in this work

Name	Description	Source
*E. coli*		
JM109	General cloning host for plasmid manipulation	Novagen
ET12567/PUZ8002	Donor strain for conjugation between *E. coli* and *Streptomyces*	[44]
*S. coelicolor*		
M145	The model organism of *Streptomyces*	[44]
M1146	Derivative of M145, completely lacks the biosynthetic clusters of Act, Red, CDA and yCPK	[31]
*S. venezuelae*		
ISP5230	Wild-type strain for jadomycin B production	[49]
WVR2006	Jadomycin biosynthetic gene cluster deletion mutant	[32]
*S. albus* J1074	*S. albus* G1 (DSM 41398) derivative with the defective SalGI restriction modification system heterologous host	[33]
Plasmids		
pIJ8660	Containing the reporter gene *gfp* between the major transcription terminator of phage *tfd* and *to*	[42]
pDR4-E	Containing ermE* promoter	[12]
pIMermE*	Insert ermE*p into pIJ8660 to control the expression of *gfp*	This work
pIM4503	Replace ermE*p and RBS of pIMermE* with P*sco*4503	This work
pIM4508	Replace ermE*p and RBS of pIMermE* with P*sco*4508	This work
pIM6740	Replace ermE*p and RBS of pIMermE* with P*sco*6740	This work
pIM3410	Replace ermE*p and RBS of pIMermE* with P*sco*3410	This work
pIM4658	Replace ermE*p and RBS of pIMermE* with P*sco*4658	This work
pIM2468	Replace ermE*p and RBS of pIMermE* with P*sco*2468	This work
pIM2074	Replace ermE*p and RBS of pIMermE* with P*sco*2074	This work
pIM5768	Replace ermE*p and RBS of pIMermE* with P*sco*5768	This work
pLN0	Remove the elements for site-specific recombination of pIMermE*	This work
pLNermE*	Insert 5′ end of *jadJ* gene into pLN0 under the control of ermE*p	This work
pLNsco6740p	Replace ermE*p and RBS of pLNermE* with P*sco*6740	This work
pLNsco5768p	Replace ermE*p and RBS of pLNermE* with P*sco*5768	This work
pLNsco2074p	Replace ermE*p and RBS of pLNermE* with P*sco*2074	This work
pLNsco2468p	Replace ermE*p and RBS of pLNermE* with P*sco*2468	This work

### Analysis of microarray transcriptome data of *S. coelicolor*

All of the transcriptome microarrays were available from the NCBI GEO database with the accession numbers GSE2983, GSE18489, GSE30569, GSE44415, GSE30570, GSE31068, GSE53562, and GSE53563 (Additional file 1: Table S1). For each transcriptome microarray, the transcript signals of each gene were normalized to that obtained at the first sampling time point (or control). If all of the fold changes (increased or decreased) of a gene were no more than two ($\left| {{ \log }_{2}^{\text{fold change}} } \right| \le 1$), this gene was thought to have a constant expression profile, and its promoter was considered to be constitutive.

### Evaluation of promoter strength based on RNA-Seq

Cultures of M145 in liquid SMM were sampled at 24 h and used for massively parallel cDNA sequencing. The cDNA libraries were prepared and analyzed on Illumina HiSeq 2000 platform. Samples were sequenced twice to obtain appropriate deep sequencing results. Raw data were processed by removing those with low quality (phred quality <5) and sequencing adaptors. The remaining clean reads were aligned to the edited genome of *S. coelicolor*. Mapping the total number of reads to each gene was implemented by Picard tools (http://picard.sourceforge.net/). Promoter strength could be reflected by RPKM, which was calculated with the following formula [39].

$$ {\text{RPKM = }}\frac{\text{total reads of the target gene (million)}}{{{\text{total reads mapped in the genome }}\left( {\text{million}} \right) \times {\text{length of the target gene (kb)}}}} $$

### Operon analysis based on RNA-Seq

Operons were predicted by DOOR 2.0 [40] and Rockhopper [41] based on RNA-Seq data and manually confirmed.

### Construction of plasmids and strains

Primers used for plasmid and strain construction are listed in Additional file 7: Table S3. To characterize the identified the promoters, plasmid pIJ8660 [42], which can integrated into the *Streptomyces* chromosome by site-specific recombination at the phage Ф31 attachment site, was amplified using primers IJF and IJR to generate the linear fragment pIJ8660 containing the *gfp* reporter gene. By using the genomic DNA of *S. coelicolor* M145 as template, the 500-bp upstream regions of gene *sco*4503, *sco*4508, *sco*6740, *sco*3410, *sco*4658, *sco*2468, *sco*2074 and *sco*5768 were amplified with primer pairs of 4503F and 4503R, 4508F and 4508R, 6740F and 6740R, 3410F and 3410R, 4658F and 4658R, 2468F and 2468R, 2074F and 2074R and 5768F and 5768R, respectively. Promoter ermE* was amplified from pDR4-E using the primer pair ermEF and ermER. Each PCR product contained the promoter and its related 5′ UTR. These PCR products were purified and then linked with pIJ8660 using the Gibson assembly method [43] to generate the target promoters-controlled GFP reporters pIM4503, pIM4508, pIM6740, pIM3410, pIM4658, pIM2468, pIM2074, pIM5768 and pIMermE*, respectively.

To engineer *S*. *venezuelae* ISP5230 to enable jadomycin B production using the evaluated promoters, the fragment of pIMermE* was amplified using primers LNF and LNR and self-ligated to remove the elements for site-specific recombination, generating pLN0. The fragment of pLN0 was amplified using primers LN0F and LN0R to remove the gene of *gfp*, and 5′ end of the *jadJ* gene was amplified using primers JF and JR. Then the two fragments were assembled using the Gibson assembly method to generate pLNermE*. To replace ermE* with other identified promoters, the fragment of pLN1 was treated with *Bsa*I to remove ermE*, and the promoters of *sco*6740, *sco*5768, *sco*2074 and *sco*2468 were amplified using primers 6740F and 6740R1, 5768F and 5768R1, 2074F and 2074R1 and 2468F and 2468R1, respectively. Then the fragment of pLN1 and the four promoters were assembled using the Gibson assembly method to generate pLNsco6740p, pLNsco5768p, pLNsco2074p, and pLNsco2468p.

The plasmid-containing *E. coli* JM109 was co-cultured with *S. coelicolor*, *S. venezuelae*, and *S. albus*, generating the corresponding *Streptomyces* transformants by conjugation between *E. coli* and *Streptomyces* species [44]. The recombinants were confirmed by PCR with the primer *gfp*-cx-R and the corresponding forward primer of the eight genes.

### Measurement of flu**o**rescence intensity of GFP

Cultures (1 mL) were sampled at appropriate times, and the supernatant was removed by centrifugation (4 °C, 10,000×*g*). Cells were washed with deionized water three times. For *S. coelicolor* derivatives, the pellets were resuspended in 1 mL binding buffer (20 mM Tris–HCl, 0.5 M NaCl, 10 % (v/v) glycerol and 20 mM imidazole, pH 7.4) and subjected to ultrasonication (power 30 W, pulse 10 s, stop 10 s, for 5 min) to generate cell extract. The quantification of GFP fluorescence intensity was implemented by normalizing the fluorescence intensity to the protein concentration of 200 μL total protein [45, 46]. The concentration of the total protein was assayed by the Bradford method [47]. For derivatives of *S. venezuelae* and *S. albus*, the GFP fluorescence intensity was quantified by normalizing the fluorescence intensity of 200 μL deionized water resuspensions to the wet weight of cells in 200 μL cultures [45]. The GFP fluorescence was detected using a microplate reader (BioTek, USA) at the absorption wavelengths of 488 and 529 nm. All experiments were repeated in triplicate. Differences among GFP fluorescence intensities obtained at different growth stages were analyzed by one-way analysis of variance (ANOVA), and *p* value  < 0.05 was considered statistically significant.

### Total RNA isolation

Cells of *S. coelicolor* (24, 48, and 60 h), *S. venezuelae* (14, 20, and 30 h), and *S. albus* (36, 48, and 72 h) were quickly harvested by fast filtration, flash frozen in liquid nitrogen, and ground into powder for total RNA extraction using TRNzol according to the manufacturer’s instructions (Tiangen, China). The integrity and quantity of the isolated RNA were checked by denaturing agarose gel electrophoresis and NanoDrop 2000 spectrophotometer (NanoDrop Technologies, USA), respectively.

### Measurement of mRNA level of *gfp*

Strains with the GFP reporter were cultured to determine the promoter strength according to the relative mRNA level of *gfp*. For real-time RT-qPCR experiments, the first-strand synthesis of cDNA was carried out using 1 μg total RNA with a PrimeScript™ RT Reagent kit with gDNA Eraser (TaKaRa, Japan) following the manufacturer’s instructions. Oligonucleotides used for real-time RT-qPCR are listed in Additional file 7: Table S3. The PCR procedures were as follows: reactions were performed in 72-well plates using an ABI7500 (Applied Biosystems, USA). The PCR mixtures were prepared by following the introduction of SYBR^®^*Premix Ex Taq*™ II (Tli RNaseH Plus) and ROX plus (TaKaRa, Japan). Each 20-μl reaction contained 10 μL 2 × PreMix, 0.5 μL of each primer (10 μM) and 2 μL fivefold diluted cDNA. The reaction parameters were as follows: 95 °C for 30 s, followed by 45 two-step amplification cycles consisting of denaturation at 95 °C for 5 s, and annealing at 60 °C for 40 s. The amplification specificity of each assay was confirmed by melting curve analysis carried out at 60–95 °C. Results were collected and analyzed using the supporting 7500 software (v2.0.4). The *hrdB* gene was used as an internal control [12]. All samples were run in triplicate. Differences among the relative levels of *gfp* obtained at different growth stages were analyzed by one-way ANOVA, and *p*-value  < 0.05 was considered statistically significant.

### Measurement of jadomycin B production

The *S. venezuelae* strains that were engineered for jadomycin B production were cultured in glucose-MOPS medium at 250 rpm and 28 °C for 48 h. Extraction and high-performance liquid chromatography analysis of jadomycin B were performed as described previously [48].

## Additional files

10.1186/s12934-015-0351-0 Overview of the transcriptome microarrays used in this work.
10.1186/s12934-015-0351-0 Genes with constant transcript levels in five time-series microarray datasets and their intersection.
10.1186/s12934-015-0351-0 Genes eliminated in each rational selection step.
10.1186/s12934-015-0351-0 Genes with highly stable expression profiles in different conditions generated by step-by-step analysis.
10.1186/s12934-015-0351-0 The 500-bp regions upstream of the 166 genes with constant expression profiles.
10.1186/s12934-015-0351-0 Growth curves of *S. coelicolor* M1146, *S. venezuelae* WVR2006, and *S. albus* J1074.
10.1186/s12934-015-0351-0 Primers used in this work.
