# Supplementary material for: Genome-wide identification and evaluation of constitutive promoters in streptomycetes
Source: Microb Cell Fact. 2015 Oct 29;14:172. doi: 10.1186/s12934-015-0351-0 (PMC4625935; doi:10.1186/s12934-015-0351-0)
Supplement: Supplementary file 1 — 10.1186/s12934-015-0351-0 Overview of the transcriptome microarrays used in this work. [file 12934_2015_351_MOESM1_ESM.pdf]

Table S1 Overview of all transcriptome microarray datasets of *Streptomyces coelicolor* used in the present work

Microarray	*S. coelicolor* strain	Culture medium (liquid)	Culture tempreature	Fermentation vessel[a]	Sampling number	Gene number	Source
GSE2983	M145	Modified R5 medium	28 ℃	NA	10	4960	Huang et al. [1]
GSE18489	M145	Fermentation medium	30 ℃	3-L Fermentor	32	8205	Nieselt et al. [2]
GSE30569	M145	SSBM-E	30 ℃	3-L Fermentor	30	8205	Waldvogel et al. [3]
GSE53562	M145	SMM	28 ℃	250 ml Flask	7	7729	Our lab
GSE44415	M145	AM media	37 ℃	NA	13	7715	Strakova et al. [4]
GSE30570	The *glnK* mutant (SCglnK-3) of M145	SSBM-E	30 ℃	3-L Fermentor	16	7,893	Waldvogel et al.[3]
GSE31068	The *phoP* mutant (INB201) of M145	The same as GSE18489	30 ℃	3-L Fermentor	36	7,893	Thomas et al.[5]
GSE53563	M145 and its *scbR*2 knock-out mutant	SMM +/- jadomycin B	28 ℃	250 ml Flask	1	7,729	Our lab

[a] NA indicates the fermentation vessel is unknown from the corresponding reference.

Reference

1. Huang J, Lih CJ, Pan KH, Cohen SN: **Global analysis of growth phase responsive gene expression and regulation of antibiotic biosynthetic pathways in *Streptomyces coelicolor* using DNA microarrays.** *Genes Dev* 2001, **15:**3183-3192.

2. Nieselt K, Battke F, Herbig A, Bruheim P, Wentzel A, Jakobsen O, Sletta H, Alam M, Merlo M, Moore J, et al: **The dynamic architecture of the metabolic switch in *Streptomyces coelicolor*.** *BMC Genomics* 2010, **11:**10.

3. Waldvogel E, Herbig A, Battke F, Amin R, Nentwich M, Nieselt K, Ellingsen TE, Wentzel A, Hodgson DA, Wohlleben W, Mast Y: **The PII protein GlnK is a pleiotropic regulator for morphological differentiation and secondary metabolism in *Streptomyces coelicolor*.** *Appl Microbiol Biotechnol* 2011, **92:**1219-1236.

4. Strakova E, Zikova A, Vohradsky J: **Inference of sigma factor controlled networks by using numerical modeling applied to microarray time series data of the germinating prokaryote.** *Nucleic Acids Re* 2014, **42:**748-763.

5. Thomas L, Hodgson DA, Wentzel A, Nieselt K, Ellingsen TE, Moore J, Morrissey ER, Legaie R, Consortium TS, Wohlleben W, et al: **Metabolic Switches and Adaptations Deduced from the Proteomes of *Streptomyces coelicolor* Wild type and *phoP* mutant grown in batch culture.** *Mol Cell Proteom* 2012, **11**.
