# Supplementary material for: Genome-wide identification and evaluation of constitutive promoters in streptomycetes
Source: Microb Cell Fact. 2015 Oct 29;14:172. doi: 10.1186/s12934-015-0351-0 (PMC4625935; doi:10.1186/s12934-015-0351-0)
Supplement: Supplementary file 5 — 10.1186/s12934-015-0351-0 The 500-bp regions upstream of the 166 genes with constant expression profiles. [file 12934_2015_351_MOESM5_ESM.pdf]

Table S2 The 500 bp upstream regions of the 166 genes with stable expression profiles

Genes	Sequences (5'-3')
SCO0068	GTTCCTGTCCTACGCGCACACCTCGCTCTACGACACCCAGACCGGTCTGGTGCTGATCTACACGGCGATCGCGGTCCCGT<br>TCGCGTTCTTCGTCATGCGCAACTACGCCCTGACGCTGCCGCGGGAGGTCGTGGAGGCCGCCCGCATGGACGGTGCCTC<br>GTGGTGGCGGGTGTTCTGGCAGATCCACGTTCCGCTGACCCGCTCCGCCATGATCGCCGTGTTCGTGTTCCAGTTCGTGG<br>CCGTCTGGAACGACCTGATGTTCGGCATCACCATGGTCACCAGCCGCAACATCCGCCCCGTCATGGCCGCGCTCGCCGAC<br>CTGCAGGGCAACTACTCCAACGTCGGGCCGCCCATCGTCCTGGCCGGTGCCCTCCTGGTGTCGCTGCCGACCGTGGTGC<br>TGTTCTTCTCCGCCCAGCGCTTCTTCGTCAGCAGCCTCAAGATCCACCGCTGAGCCCGCGTGCGCCCGCACCTCCCCCGT<br>CATCGTCGCGAAGGGAACACCC
SCO0069	ACCGCGGCGACGACGGCAGCACCAACGAGACGCTGTGGACCTACAACTCGGGCGCCATGATCGGCACCGCCACCGCGCT<br>CTACCGCGGCACGGGTGACGCCCGGTACCTGGAGAAGGCGGTGGCGGACGCCCGCGGCTCCCTGGCGTACTGGACCGG<br>GGGCACCCGCCTGCACGACCAGCCGGCCGTGTTCAACGCCTTCTACTTCAAGGACCTGCTCGACCTGGACGCCGTCCGCC<br>CGGACTCCGCCTATCTGTCGGCCATGAGCGCGTACGCCGACAACACCTACCGGTCCAACCGGGACGCCTCCAGCGGCCT<br>GTTCCGCTTCCAGCCCTCCAACGGCGGCGACTACGACCCCGCCGCGCCGGCCGCGACCCTCAACCAGTCGGCCATGGTC<br>CAGATCTTCGCCACCCTCGCCGACGCCACCCGCCGGGACCACCGGACTTCCTGACTCGGCGGACCGCGCCGCCGCTCCC<br>CTCGACGCAAAGGACCTTCACGCACC
SCO0119	GCAGAATCCCCTCTCCAGCTCCGGGTCCGACAGTGCCGTCGTGCGCTTCCTCCCGACGTACGGAGCGGTACAGGCCAGC<br>ATGTCCGCCGCCTTCTCCGACGCGTCGGGCGGCCGGGCGCTCCTGACCCAGTGCCTCTGGCTCACCGGCGCGTCGGCG<br>GCGGCATTCCTGGTCTTCCGCCGACGTACCCGGAACGCGCTCGTGCGGCACAGGAAGCCGCCGTTTCCGGCGCCCCGCG<br>GCGCTGGAGGAACCGAGGGCCCAGGCGTTCACCGGCCCCCGGCGGAGTCCGGCCCGGCGCCGGAAAGGGTGGGACCC<br>GCCTGACCGCCCGGCTGCCCCGGGGCCGCTCGGGGGCTCCGCCCATCGCACGGACGGCCATCGACCCCGTGTGCGCCC<br>CCTGTCCCGCCGATACGCCCGTGACCTGGGCAGGCGGTGATCGTTCAGCTGTCGGGCGCTGGATCGGTGCCCGGAACGA<br>CCCGTTCACCACTGCAAGGAGCACCGCCCC
SCO0123	CGGACGGAACACACCCATTGATCCGTCCTGGCCACCCGAACGGAGGATCTCGCTCGACCGTGGGTGCCGCCGGTGCACG<br>CCGCTCGGGCCGGTCCCTCTTTCGGTGGAACTCGTCACGGTGCACCGGGATCACACCCTCGGAGGCCGCGCCCCGGGAC<br>ACGGCCGCGCGCGCCAGGCGTCGGAGGCCCGCCGCGGCACGGGAAAACGAGGCCCCGAGGTGCGGAGAGCGGGTGGG<br>CGGCGACCGGCGCGGAACCGTCCGGTACCCGCCCGCGGAGCCGGGACGGGCCACCGCAGCCGGCCGGCGCAGCCCCG<br>GTCGCCGGTCATCCTGGTGAGCCGCCGGTCCCGGCAGCGCGACCGGCTCGGGTGGGTGGGGCGCCCGGCCATTGGATT<br>CCCCGCCCTTCGCGTGCTGCCGTACCTCTGTCCCACCACCGGTGGTCATCGCGACCGCCTCGGCCCGAACGGAGCCCG<br>CACCCGCATGCCCGATTCCGAGCAGTCCACC

SCO0265

ACTGGATCCCATCGGTCTGTGGTCCGCCTTCAAGCGCGCCCACGAGGTGGTCCGAACCCGCGTGATAGCCGACGCCGCG
GAGGCGGCGGGCCTCTCCGAGCCGGACCTGACCATCCTCGCCGACCTCAACAAGGCGGGAGGCGCCTTGCGCCAGAGC
GAACTCGCCGCGTCGCTCGGCTGGGACCGCACCCGCGTCTCCCACCAGCTCACCCGCATGAGCAAGCGCGAACTCGTCA
CCCGGCGGCGCGCCAACGGCGTGATCGTCGTTCTCACGGACGCCGGGCATCAGGCCATCACCGCCGTGTACCCCGGCCT
GGACGCCGCGGTACGCCGCCACTTCACCGACAAGCTCTCCACGCAGGAGGTCGAAACACTGGCCGCCGTCCTTGACCGG
CTGTAACCGTCCCGGCCCCGCCACCACACGGTGTGCCGGCGCGACGGGCGAGAGTCACCACGGAGTGTCACGCTGGATC
ACACAGGAACTGCGAAAGGGAACGTCC

SCO0267

GGCCGCGCGCCGCGTCGACGAAGGGCCAGTCGGCGGCGGGGACGATCCAGTGCGCTGCCGGGTCGGGGGAGGCGACG
AGTTCGCGGGTGGCGTGGGTGACGGGAGCGGTCGGCACGAACAGTACGAGGTGCCCCTCCACCTCGGCGACGTCGTCC
CACCGGTGCACCGCGCCGGGGGGAATCCACACCGCGGACCGGTCCTGGAGGGGGTGGAGGCGGAAGTCGACCGTGACG
GAGCCGCGTCCGGCGTCGACGACGGCAAGGACGTGGAAGTCGGCGCGCTGCGTGCCGCCGTCGTTCAGCTCGCGGAGG
CGGCCGAACGTCATCGTCTCGACCGAGGCGGCGCCGCGGCCGGGGGGCTGGTAGGTCATCTGCCGGATCGCGCTCACG
TGTCCAGTTTTCACCATCGCGCGACCGCTCACGCCGATGCCCGGTCCGGGTGCGAGGGGTTCGATGGACACACGGGGCA
GCCGCCCCGGGCATCGAACGAGAAGAGCCATC

SCO0285

AGAGCGTGGGCGAGCGCGGCGCCGGCGACGGCAACCCGATCACCATCGGCGGCACGACCTTCGCCAAGGGACTCGGCG
TCCACGCGTCCAGCAGCGTCGAGTACTACCTCGGCGGCACGTGCGGCAAGGTGACCGCGCAGGTCGGCGTCGACGACGA
GTCGGGCGACAAGGGCACCGTGGCCTTCGAGGTCTGGGCCGACGAGACCCGGGCCGCGTCGACGGGCACGCTGACCAA
CGCCGATCCCGCCCGCGCGGTCTCCGCCGACGTCTCCGGCGCGGACATCGTCCGACTGGTGGTCACGGACGCCGGAGA
CGGCAGCGGATACGACCACGCGGACTGGGCGGACCTGCGGGTCACCTGCACCTGAGTCACCTGGCGCGCCGCTCCGGT
CGCCCTCGCCCCGAGCCGGAAGTCGCACGGTCGGACCGTTCCGGTTGGTGCCACTCAGCGGTCTTCCGTGTCAGGTGGG
AGAGGGCTCCGGGCGAATGGGGCCCGAGCA

SCO0295

CCGCCCGCCGGCCCTGCAGGCCGCTGCTCGACAGCGCGACCGCCAGGTGCAGTACCGCCGGACCGTCGGGCCCGGTGA
TCTCGCGGGCCACTTGGGCCGCGTACGTACGCAGGTCGCCGGCGAGGCTCCCGGTGTCGGGCATCGGGGACCGCGCGT
TGAGGCGGGTGAGCGCCACGTCGGTGAGCAGGGTCTCGAGGCTGCCCCACCGGCGGTAGATGCTGCTGTCGGCCACGC
CCGCGCGGTCCGCGACGTCGCCGACGGTGAACCTGCCGTAGCCGCGCTCACTGATCAGATCGGTGACGGCCTGGTGTAC
CTGCGCTCCGACACGGGCACTGCGCCCGCCGGGCCGCCGGGCTCGCTGTTGCTCGTTCATGCCCCCACCTTAACGCAGT
CAGTACTTGCGTTTGCGGGGGAGCCTCCCTATAGTCTGCTAACGCAGTCACAGACTGCTTTAGGAGCGCTCGGATGGCCG
GGCGCAGCCAACGACGGAGGGGGATCCC

SCO0301	TGGATGCCGGGGTAACGGTTCCAGTACCAGGTGCCTCCGAAGTCGCCGCCCTTCTCGACGACGCGGACGCGCGCGACCC<br>CCTGCTGACGCAGCCGCGCCCCCGCGAGGACGCCGCCGAATCCGCCGCCGACGACCGCGACGTCCACCCGGTCGCGCA<br>CCGGTTCGCGCACGTCCGACTCCCCCGCCCAGGGGTCGGCGGCGTAGAAGCCGAACTCGGCGTCCGCGCCGAGGTACT<br>GCCGGGTGCCGTCGGGGCGTACGCGTCGCTCCCGCTCGCGCCGGTACCGCTCCCTGAGTCCGGCGAGCGCCTCGGGTG<br>TCAGCTCCTGTGCATGTGCCATGTGGGGGACCGTGTTCCTTCCGCTTCGCCGTCAGGAAGCCACTCGTCCGTGAATCGGA<br>CTGGAAACAACGGGACTTGAGCAGCCATGCACTACGGTAACAAGAACCGGACAAGGTTGTCCGGTTGGCTGCGGGTTGCT<br>TCCGCGTACTTTCGGACAGGAAGAGTG
SCO0304	CGCGCTGGAGCTGTTTCGCGAGTGGCGCGTTCTTGCTCTGGCTGTACGAGATGAAGGCGTCGCGCTGCTTCGCACCGAC<br>CGGCTCCACCGCTCGCGCCTCGGCCTCTTCCACGGCATCGCTCATGGCGTCCCCCGTGCCCCCTCCGCATGTACCTGTGG<br>CCCCCGACGTGCGGCACCAACTCCACTGCCTATCATGCCCGTTGTGCCCCACGGGCTCACCCGCACCACCGCGCCCCGA<br>CCACGACCACGACCGCGGCCGCGCCGACGGTCGCCGACCGCCCCGGGAACGCTGACCACCCCGCCCACCGCCCCTCTC<br>TCGGGACGACGAGCAGGCCACATCGTCCGCCGGAACGGCTTCACGCCCTTCGCCGTCTCCCTCGCCCACCGACCCGCAT<br>GCGGCGGGTGCGGCGTTGTCCGGGCCGGGCAGACGGTTTCGCCGATGAGTTCAGTCTCCTCGAACGGTCTCACCCAGCG<br>ACCCGACCGTTCCCGACAGGAGTGCC
SCO0309	CGGGCGGGCGCTGCGGGAGTTCACCGAACTCATCCGCTCGCATGCCGAGATGGCCTTCCGGGACCGGGCGCGGCAACT<br>CGGCGTGGAGCCCAGGATCCCGCTCGGCATCGTCGCCCGCGCGTGGACCGGAGAACTCATCTCGGTGCTGGGCTGGTG<br>GGTCGAGAACGACACGGGTTACACCGCCGAGCAGATCACCGCCCACCTGCGGGATCTCTCCGTCTACGGCAGGGTCTGG<br>GCCACCGGGCTGACCCCGGTGGACGCACCCGGAGCCGTCGACCTCCCCGGGGGGCGGGGCGCCGAACCCGTGTAGTCA<br>CGCCCCGGCGGCCGACCCCTTCCTCCGTTTCCGAACAGCGTGTACGGTATCGATCTCGCGATGAGCTGATTCCGAACGAG<br>CGGAGCGACAGGTGACCGGCACGGACTCACTGGCCTACCTCGTCTGGGCACGAGGATTCACGGACTCGGCCTGGGCACG<br>ACGACACACGACGGCAGGCCGGGAGATC
SCO0322	CGCAGGGATTCGCTGCGGCCGGACCGGCGCTCAGGGTGATGGCGCCGGCGGAGGCGAGTGCCAGGGCGAGGGAGGCG<br>AGGGCGCTGGTCACGATACGACGAAGGGGCAAGGGGAGCTCCAAACGGTGGGGGATCGCCAGGCGGGACTGCCGCTGT<br>CTGCCGATCCGTTGTCGATGCGACGCCCAGACTCACAGCATCGGCGCGGGCGCTCTGAGCAATGGCGGCGCCGTTCACC<br>CGGCGTAACGATTCACTGCTGCGCCGATCAGTTGATCTCCACCGGTTCACGGTGCACCGCGCTCGGGGGCGCACCGACG<br>AACAAGGCGAGACGTATCGTCTTGCCAATGGGTGCGGCGTGGCGTACCGTCCTTGGCATGGGTTCCCTGTGAGATCACCG<br>GCGCCGACGGCGTAGAGCACCGCTCCGCCGTCGCCGCGCCTCGCCGTGCCGTCCACCCACCCCGATCCGGCCCGGGCC<br>GATCCCGTGCGGCCAGGAGGGACCCGATC

SCO0329	CAAAGATCCACCTCGCCTGCGACGGTGAAGGCCGCCCCCTGGCCTTCACCCTCACCGCCGGCAACGTCAACGACTGCACC<br>CAGTTCGAGCAGGTCATGGCCCGCATCCGCATTCAGCGGTGCGGACCCGGCAGACCCAGAACCAGGCCCGAGCGGGTCG<br>CGGCGGACAAGGGCTACTCGTCCACGAAGATCCGCACCTACCTGCGACGTCGCGGCATCAAGGCGGCGATTCCCGAGCG<br>CATCGACCAGATCAACGGCCGCATCCGCAGGGGCGAGAGCCTCTGCCGACTCGACCGGGCGGCCTACCGGCGACGCAAC<br>GTCGTCGAGCGCTGCTTCAACAAGCTCAAGCACAACAAAGCCCTGGCCACCCGCTACGACAAACGCGCCCGTCACTACCA<br>GGCCCTGGTCACCCTGGCCTGCCTCAAACTCTGGCTACCCTGACTTTGCGGACACGACCTAGAGACCGCCACCGGTACGG<br>GCCCGGACGGAGGAGGCGACACG
SCO0341	TGGACAGCGGCGACATGGGCGCCTTCTACACCTACGGCGTCAACAACGTCGAGCCGTACCCGATGGACAACCACGTGCGC<br>CAGATGGTCGTCGGCGACGTGATCCCGGACGAGTCGATGCCCGACAGCGGCACCCGGGGCATTCACATGGACGCCGGCG<br>GCTGCGGCTTCACCTTCGAGGACATCGAGGTGGGCAGGACCACCGACCAGTCGTACCAGAGCTACCAGTGCAACGACGT<br>GAAGAACACCAACTGGGAGGACGGGTACGACGCCTCCAGGATGGAGTACGACAAGATCGGCGTCACCGCCGGCTTCCCC<br>TACCCGCTGCCCGACGGGGACACCCAGTGAGCGGTCGCTGACCGGTCGCCCCCCGGCGAACACCCCCGGGCAGCGTGC<br>CGCACGAGCCGCTGCCCGGGGTGCGGTGGGCGCGGGCTCCCGGACCCGCCCGCGCCCGGCACAGAGCATTCGCACCG<br>CCCTCGACACATGCCTGGAGGCTTTGCC
SCO0371	GGGCCCCACCCACGGCGCCCTCCTGGAGCTGGGTCGGGTCAGCGACACCTTCCGGGTGAGCGTCAACGGCCGTGACGC<br>GGAACCGGCCGACCGCCTCGACCCCGTCGTCGACGTGGGCCCGCTGCTGCGCCGCGGCACGAACACCATCGAGATCGA<br>GGTCGCGACGCCCCTCGTCAACCGGCTGAGGGTGAGCCGCCCGGAGGTCTTCGGAGGGCTCGCCCGCCAGGAGTACGG<br>GCTGGTGGGACCGGTGCGACTGGTTCCGTACGCGCAGAAGACGGTCTGACTCCACTCGAATTGAACGATTCAAAACAGCG<br>CGGACGGTGCCGAGGCCCGTCCGCGCCGGGTCATGCCCGTTCCGCGCCCGGTGACTCGCGGTTCGGAGGGACTTATGG<br>CGCGTCAGAAGGTTCTTGTGGGAACCCTTGACGCTCCTGGTGACGCCACTTAGCGTCTCCGGAGAATCGAGTAAAACGATT<br>CAATCGAAGGGAGCTGAGCCCCGCACC
SCO0373	GGCGAGACCGGTGAGCACCTGGCAGCCGAGCAGCAGCTGCACGGCCCGCCGGTCGGTCCGCCACGACAGCCGTGCGAT<br>CCGGCCCAGGACGGCCGGGATCTGTGCGCACATCCGTCGGAAGGAGACCTCGGCGAGCGGATTGACGGAATACCGTCCG<br>CTGTAGGTGATCTCCGCCGGAGGCGGTGGAGGCGGTGGAGGCGGTGGAGGAGGTGGAGGCGTGGGTTTCCGCGGTCCG<br>TTCGCGGCGGTCGAGGTCAATGGATCCCCCTTGACGGTCGTTGAGCGTGGTCGTGAAGCTCAACGACCGGACCGAGGTAT<br>CGAACACATGTGTTTCGGTCGCGGTCAGGGCCCCGAACCACGGCGCGTGTCGTTTCGTGGCGGATGCACGGACCCGTGC<br>GGGACAACAGGGGTGCGGAGCGGGTAGGTTGGCCGAATCGCCGACAACCGCCCGCCCGGCGGTGGCCGGACGCCGGG<br>AACGATGCGGCGGTCGAACCGCCAGGCCG

SCO0444	TACAACGGCGGACGCGACGAGTTCGGCCGGCAGGTCGCCAACCCGGCCGGCATCGACCTGGCCGACGGCACCTTCTACA<br>ACGTCGGCCTGAACGACAACGGCTGGGTGACCGTCACCTACCTGTGGACCGAGGGCGGCGGCGACACCACGTCCTTCCC<br>GACCTGGGGCACCGACGTGAGCGTGCGGCAGCGGGCGACGACCGACTCCACCCGCGTGGCGAGCCTGCCCGGGCCCAC<br>GACCGTACGGGTGCGGTGCCAGGTCCACGGCCAGCTCGTGGACTACGACGGCTACAGCAACGACGCCTGGTCGTACCTG<br>CCCGACTACGGTGGCTACGTCTCGAACATCTTCATCGACGTGGCCGACTCCTGGCTCCCGGGCGTGCCCACCTGCTGACC<br>GTCGGGCAGCGCGGACCCCGACGCCGCCACGTGCGGCGTCGACTCGTCCACCAGGGCCCGGGGGCCGTGCGCGGAAG<br>AGGGACCGCTGCCACGCACAATGGGCGC
SCO0453	ACCGACGAGCAGGTCGGGGACTTCCGGGTGGTGCCCTGCGCGGTGTGCGGCGGCGTCCTCAAACCGGACGTGGTGTTCT<br>TCGGCGAGAACGTACCGCCGCGCCGCGTCGAGCACTGCCGCGAGCTGGTGCGCGGTGCGTCCTCGCTGCTGGTCCTCG<br>GCTCCTCGCTGACGGTGATGTCCGGGCTCCGCTTCGTCCGCCAGGCGGCCGAGGCGGGCAAGCCGGTCCTGATCGTCAA<br>CCGCGACGCCACCCGCGGCGACCGCCTGGCCGTCACCAGGGTCGCGCTCCCGCTGGGACCGGCCCTGACCACCGTGGC<br>GGACCGGCTCGGCCTCCGTGTGGGGGACGCGGCGACGGCCTGACCAGGCAGCCCGCGTCGCGCACGGGACCGGAAATC<br>TCCCCGTGCCACCCCCTTGACCGTGGTCCCGCCACTCCATACAGTCCTCGCACCTCACGAGGTACATCGATTAACCAATCG<br>TTAACCGCTTGGTTGGAGACCACCCCT
SCO0468	CCTCGGCGTACGCGGTACTCTGCGCGCCCGCGTCGGCGGGATGCGTGGTGGCCTCCGCGAGGGCCAGCGCGGCGCGTT<br>CCCTGGGCGAGAACAGCTCGGTGTCGCGCCAGGCCGGCAGCACGCCGAGCCGCTGGGTGGTCTCGCCGCCGCGCAGCG<br>CGGCCCTGGTGTGCGCGTTGAGGCAGTAGGCACAGGCGTTGAGCTGGGAGACGCGGATGTTGACCAGTTCCACGAGGAT<br>CCGGTCGAGCCCGGCCGCCTCGGCGACGCCCCGGACCGCCTCCGCCGTGGCACGCAGTGCCGCGTAGGCCTCCGGGCT<br>CTGCTTGTCGACGAACACACGTCGTTCGCCGGTGCTGCCGGTGCTACCAGGGGCGCCTGCGTCGGCGCGTACAGGCGTC<br>TGTGTCATGTGGGGTGTCCTCCGTCGCGCGATACCGTTTCTCACGTCCTATCATCGAACCATAGAAGTTGAAACTGAAACTT<br>TCAGGGGCGAGTGGGAGGGCCGAGCG
SCO0481	CCTCTTCGCAGCGGAACACTTCCTCGGCGGGATCTCCGGCTACGAGGCCGCGGAGACCACCGACGAGAACCCCGCCGCC<br>CACGTCGCCGTGGTCCGACGGGTGCCCCTGGCCCACCTCCGCCACACCCTCGGCGTCGACAGCACCGACTGGACGGCG<br>GCGCGCAGGACCCTGGCCGGAACGGCCCATCCCCTCGGCCGACTCGAAGCCGAGTGAGCCCACTCCGCGAGACAACCCC<br>CGCAACCCCGCGCGACGGACACCGCCGTTGACACACTCTTGACACGGTCACGTCGACCGCTTGTATGGTCTAGTCCAACA<br>GAACTCGCCGCTCCGTTTACGGAGTTGAGCCCTGCCGGGGGTCCCACGCCCCGGCGGGGACGGTGAGTGCGCGTCACCT<br>TCGCGCCTCCTGGCCCCACCCACGAGAGGCCGTACTCGGCGTGCGCGCGGTCCCCCCACACACGGCCGCGCGTGCCCT<br>GGGACGGCCGGATGTGAAGGAGTTCCC

SCO0483 GCTGCGTCCGGACGACACCGACGGCATCCGGGCCCGGCTCCTCGCCGAGTACGCCGGCTGGTCACCCCGGATACTCCG
GATGATCACCGAGAACGACGGCCCGTACGTCGACCGTCCGCTCTTCGCCCTGCCGGTGCCGCACACCTGGCGGCCCACG
CCGGGCGTGACCCTGCTGGGCGACGCCGCGCACCTCATGCCCCCGCTCGGCGTCGGAGTCAACCTCGCCATGCTCGACG
GCGCGGAACTCGCGCTCGCCCTCGCGGCGTCCGCCACCGTCGACGATGCCGTGCGCACCTACGAGAAGACGATGCTTCC
GCGCTCGGCCGAGATCGCCGGCATGCTCGAGGGCGGTGCCGGCTTCCTGCTGGAGGAGCCGGACGCCGAAGACCTCGC
GCGGCTCGGTGCGCCCGGAGCGGACGGCGGACCCGGCGAGTGACGCGGGCCCGGACCGCTGTGCCTGCCCGGGGAGC
GGGGCCCGCCCGGCCGGTAGGGTCGAGCCC

SCO0511 CCTCGAAGCAGCGCCCCGTGACGCCCTCCACCAGTGGGGACGCGGCCGGCAGCACGGAGGTCGCGGCGTCCTGGAGGG
GCGTCTCGCAGGAGGGCCGCTCGATGACGTTCCCGTCGTCGTCCACCGCCCCAGGACCCGCATCGTCGCGGCGTCGAGG
TGCCGCTGGAGGCTGGTGCCGATCCAGCGGGGTTGAGCGAGTTGGCGGCGATGCGGTCGGCGACCCAGTGGCGGGCGT
CCACGGCGAACAGGACGTCGGCGGTCGTGGACTGGGCGCAGGCGGCCCAGCGGTCGGAGCCTGCCGCCCGCGGCTGC
CTTCACACGGGTCACCGGCACCTCTTCGCGCGCGTACCGCCACCGGCACCGTACGGGCTGACCGAACTCTTTTCTGGACT
TGCCAGTCCATTTTATTCGCCGCACAGTGGCATCCGCGCCCCGGCCGTCGGCCGGGGACGACGGAGAAGCACACGGCTG
AGACGGCAGGCGAGGAGCAGGACATGTCG

SCO0541 GACGTTCCTGGTGATGAAGAAGCTCGGCCTCATCGACACCCTGGGCGCGCTGATCGTGCCGTCGCTGGTCACGCCGTTCG
CGGTGTTCCTGCTGCGGCAGTTCTTCCTCTCGCTGCCCAGGGAGCTGGAGGAGGCCGCCTGGATCGACGGCTGCTCCCG
GCTGCGGGTGCTGTGGCGGATCGTGCTGCCGCTGTCCCGTCCGGCGCTGGCCACGGTGGCCGTGCTGACCTTCCTGACC
ACGTGGAACGACCTGACCTGGCCGCTGATCGCCATCAACCACGACACGCAGTACACGCTGCAACTGGGCCTGACGACGTT
CCAGGGGCAGCACCACACCCAGTGGGCGGCCGTGATGGCGGGCAACGTCATCACCGTGCTGCCGGTCCTGCTCGCCTTC
CTCGGCGCGCAGAAGACCTTCATCCAGTCGATCACCTCCAGCGGACTCAAGGGCTGACCACCCGCCCTCCGTCCGCCGCC
ACCCGCAACGGAAAGAACCCCGC

SCO0554 CTGGGCCCGTGAGGCACCGGTCAAGACCCAGTCCCGGATCGACACCCCGAGGCCCTTCGCCCGGCCGAAGGCGGGCAC
CGTGATGGTCGCCGGAGTCGCCTGGGCCCAGCACCGCGGCATCGACAAGGTGGAGGTCCGCGTCGACGACGGTCCCTG
GGAGGAGGCCGTCCTGGCCGCCGAGGACTCGCGCGACACCTGGCGACAGTGGTCGTACGCCTGGCGTGCCACCAAGGG
CGGCCACACCCTCACCGTGCGGGCCACCGACCGGACCGGCGAGGTGCAGACGGACAAGCGCACCCGCACCGTGCCCGA
CGGCGCCAGTGGACGGCACTCGGTGGTGGTGACCGTGGACTGACGCCCCACCGGGCGGCAAGCGTTTTCTGTCCACTGT
ATTGACGTGTACGCGCCGTAGTGAGTTTCATGGGGTAGACGTGGGAGCGCTCCCACACATGCGCCGTTCCCGGTTCCCCC
CATCCGCACTACTCAGCCCTGGAGTCGCC

SCO0562

TCGTCCGAGGCGGTCAGACAGGGCGCGTCGCCGACGGCGCAGTCGACGTCGGCGAGGGCGCCGCAGGACCGGCACAG
GACGTGGTGGTGGTTGTCACCGACCCGTCCCTCGAACCGCGCCGGGCTGCCGGGCGGTTCGATACGGCGCACGAGCCG
TGCCGCGGTGAGGGCGTGCAGGGCGTCGTACACGGCCTGGAGGGAGACGTGTCCCACGCGTCGGCGCACCCCGGAGGT
GAGGGCCTCGGCGTCGAGGTGGTCCCCCGCCCGGACGGTCTCGAGCAGCGCGACACGGGCGGCGGTCACCCGCAGTCC
CGCCCGGCGCAGCTCGTCGGCGGTGGCCGGTTTCCGGGAGTTCCGGGATGCCGTCATGGCGGCGAACCTACTCCCTGG
GACACGAACGGCTCAAGGAAACGAACGACTCCAGCGTGGTAAACAACGTGCGTGGCGGGCACTGGCAGGTGATGCCGCC
CCGTACGGCACGACGACAGGAATGAGTGGCCG

SCO0594

GTAGGTCCAGGCCTGGCCGGGAATGGTGTAGCCGATGTCCGCGAGGCCGCCGCCGATCTCGCTGATGACGTGGTGGGCA
CCGTCCTCGTTCCCGGTGACCAGGACACCGGCGACGCGGTTGTACGCGACCGGACGGCCCTCGTCGTCGGTCTCACCGA
GCATGGCGTCCATGCGTTCCAGGACCCGCTGGGCCACGGAGGAGGGGCGGCCGAGCCAGGTCGGCGACGCGATGACCA
GGATCTGCGAGGCCAGCAGCTTCTCGTGCACGCCCGGCCAGTCGTCCCCGTCGCCCATGTCGGTCTCGACCCCCGGTTT
CAGGTTCAGGTCGACGGCCCGTACGACGTCCACCTCCGCCCCGTGGCCCTTGAGCGCCGCGATGACCGTGGCGGCCAGC
GCCTCGGTGTTCGACGGCTGGGGGGAGGGTTTGAGAGTGCAGTTGATCACGAGTGCGCGCATGCCCACCCGGGTTCCCC
GGCGGGCCTGACGCTACCGTCGGCCGT

SCO0604

CAGATGGAGGCGATCCGGGTGCCGTTCCCGGCCGCCGTGCGCAGCGCCTCCCGGACGGCCGGGGAGAGCGGCTCGGC
GGCGGCCGTACCGGGCACGATGACGGTGTCCGCGTCCCGCAGTCCGTTCAGCCCCCAGGGGGCCCGCAGTGTGAACGC
GCCGGCGTCGATCTCGGGGTGCTCGGCGCACACCCGGATCCGGTAGCCGGGACGGCCGTCGGGCAGCCGGGTACGGGT
GAAGACCTCGATCGGGGTGGACAGGTCGAACGGGATCACCCGGTCCAGCGCGAGAACGGCGACGGTGTGCATGGCCGG
AAAGTACCTCCTCGCGGGCCGGTGATCGGCGGGCGTGGCGTTTTCCCGTCGAAAGCTGTCACTCCTGCCACAGGCCGTC
GACCGAACCACTGTCCACCACAATAAGCAATTTGCCTAAGCTCATTAGGTTAATGCACAATCAAAGAAGGAAGACGCTACGC
AGCGAGACGCACCACCGCCGGAGGCGAC

SCO0614

ACGGAGACCAGGCGGCTGTTGCCGTCCCCGCAGGCGAGCACGTACGCCTCGGGCCCGATCTGCGGCTGCCCGGAGCAG
TCGAGCAGCACCGGGTCCTGCGTCTGCTGGGTGCGTGCCGCGGGCTGCGCCGACGGCGCGGCGGAGGCCGTGCCCAG
CGGTACTGCGAGCGCGGCGGCGGCACAGAGTGTGACCGCTGCCCTCATCGGGTTCCTGCCCATGTCTGCTCCCGCCGTC
TCGGGGTCGCTTGTCTCCGCTTGCCCCTTTTCTCCGTTCCGACCGTACGACCGTGTTCGTGAACGCACCACTCGTACGGAG
CAGCGGCCGGGCCCCGAGGGCGCGGACCTGCGCGTCGGCCGGGCCGTCCGGTTCGTGCGACAAGACGCCGATTGCATC
CCCCGTGTGGTCCTCCCGGCGGTCCGCAACCGCCGTCCGCGCCCCGGTGTCTCATGCCGTCAAGAACGGCGACCCGACC
GGGCGCCCCTCACGTGGAGGACCGCGGT

SCO0621 GCGTCTTCGGTGCCGTGCTGCTCGCCGTGGCCGTGGCGATGGGCGTGTCGGCGGTACTGTGACCCGCCGACCCGCCGA
CCCGCCGACCCGCCGACGCCGGCCGCCCGGCGAGCCCGCCGGCGTCAGGCCAGGGAGAGGAAGAGCTTCTCCAGCCTG
GCCCGCATCTGTGCCGAGTCGCGCACCTCGGGGTCGTCACTGGTCATGCACTGCTGCAGTCCCGTGGCGATGATCGAGA
ACCCGGCCCGGTCCAGGGCGCGGGAGACGGCGGCGAGCTGGGTGACCACGTCCTCGCAGTCCCGGCCCTCCTCGATCA
TCCGGATGACTCCCGCGAGCTGCCCCTGGGCGCGCCGCAGCCGGTTCAGCGCCGACTTCAGTTCGTCGGCCGACATGTC
GAGCTCCACGCTTCTCCCTCTCCTCCACCCGATACCTCACCGAACCCCACATACCCCCGCGGGTATCCTACCGCGCGGGG
GGACGAACAACGAGAAAGGTCTCGCTCC

SCO0647 GAACCTGATCCTGGACGCGCTGACGACCGAGCTGGACGCCGCGCTGCCGGTCCCGGACACCGGGCGGGTCCGGGACGA
CCTGCTGGCGTTCTTCTCCGCCCTGGCCCGCCTGCTGGGCACCCCGCAGGGCCGGGCGCTGCTGCGGCTGAGCGTCGA
GCACGACGACACCCTGGAGGATCGGCGCGGCCCCTACTGGAACGAGCGTCTCGACCGCGCCGTGGTGATGGTGCGGCG
GGGCGTCGGGCGCGGCGAGCTGGCCGCGGACACGGACGCCGGGCTGCTGGTCGAGGCGGTCAGCGGTCCGCTGTTCG
TGCGGGTGCTGCTCAGCGGGGCGCCGTTGGACGACGCGCTCGTACGGGGGCTGGTGGACCTGGCCCTTGCCGGGGCCC
GTCCGCGGTGAGCGGTCCGCCCCGTGGTGGACCGTGCGGGTGCGACGGCCCGGATCGGGGCACCCGGCGGTCCTCAG
GGGGCGGCGCGGGTGCGCCGAGGACGGAGGAACC

SCO0675 CTCTGCGCGTGGTCGACGATCCGGTCGGCGGAGGTGAAGGTGAAGCTGCCGCGGCTGGGCTCGGTGGCGTCCCACTTCA
TCTCGTTCTCGGGCGTCACCGAGCTGAACTCCCGGTCCAGCGTGGCGGCGTAGTCGCTCTCGCCGAGGTGGCTCGCCGC
GACGGCGGTGCCGAAGTAGCGGCCCTGTCCGGCGGCCGCGGACCCGAGCGTGTCGGCGGCTTCGGCGGAGTGGGCCG
CGGCCGTCAGCGCGGCGGCGGCCACGAGACCGGCGGTGGCCGCTCTCGACAGGCGTCTGACAATCATGCTCATGTCATC
CCGTTCCTTGAATCGGTGTGCGCAGGCCGTTCGGAAGGGCGGAAGCCGCCGGACTTCACCCGGGAAGGTCGAAAGTTTC
GGTGCGATTTCCGAATGTCGCGGAGGAGATTACGGACTCCGGTCCGCCGGGTCAACGCCCCGTCACCGCCGGATCAACG
CCCCGTCACCGCCGGGTCACGCTCACCC

SCO0677 CAGGTGGCCGAGTGGGACGTCCCGAACGATCCGGCGGCGGTCGCCGAGATCCGTTCCCAGGTCACCCGGCGGCTGGCC
GAGTGGGGGCTGGACGAGCTGGCGTTCACCACCGAGCTCATCCTGAGCGAGCTGGTCACCAACGCGATCCGGTACGGCG
GCGAGACCGTCCACGTCCGGGTGGTGCGCGACCGCAGCCTGATCTGCGAGGTGTTCGACAGCAGCAGCACCTCCCCGCA
CCTGCGCTACGCGGCGATGACGGACGAGGGCGGGCGCGGACTGTTCCTGGTCGCGCAGCTCGCCGAGCGCTGGGGCAC
CCGCTACACACCGGCCGGCAAGGTCATCTGGGCGGAGCAGCCGGTGCCCTGACGGACGCGGCCCCGGTCCCCCGCTCC
GTACCAGTTCGTGCACGACGGGTGCCCGGCACGCCCCTCTTCTGGTAGGAAACTTTCCTATCAGTGCCCGGCACTCCCCT
CCCCCACTCCCCCACACCTGGAGATTCC

SCO0686	GACGCCGAGCGCCTCGGACGTCGTCTCGTGCGTGGTCCAGGTGGTCGTCACCCGGGTCATCCGAGCACCAGCCGGTCCG<br>CCATCTCGGCCAGCCGGATGCGGGCCAGGGCGAGGTTCCCGTCGTTGCGGTCCAGCCACAGATGGAGGAAGACGGTGCT<br>GTCGAACGGGGTGCTGACGAACCTGAGCAGATGGTAGGCGTCGCCGGTGGTGGTGATCAGGTCCTCCACCGGGGGTTCC<br>TTGCCGGGCTCCACGGTGCCGCCCGCCGTACCGGCCGCCGCCAGGGAGCCGCTCTCCGCGACGTGCCGGCCCAGCTCG<br>GCCGTCTCGGCGGCCGTCGCCTCCCAGTCGCCGCCCGGCGCCTCACCGACGGCGCCGAGAGCGAGCCCGCTGATCCAG<br>TCGACCAGAGACGCTCCGCGCGCGCCTGGCAGTCGCATGGCTTCCAGCAGACTCTCGTCGATTCCGGGCACGCCGGATC<br>CCCTCCCCTGCACGGCTGTACCGCGAAC
SCO0723	GCGCAGTACGTCCCGGGTGTTGGCGCTGGAGGAGACGATCGCGGTGGCGAGGCCCGCGGCGCGGACCGCGTCGATGTA<br>GCGCAGCGTGCCCTCGTAGGGTTCCACGCCGTCGGTGCGGATCTTCTCCAGGAGGAGTTCGTTCTTGCGGTTGCCGACG<br>CCGTTGACGGTCCGCGCGTCCGGCGGATCGTCGGGGGTGCCCTCGGGCAGTTCGACGCCGCGGGAGGCGAGGAAGGAG<br>CGGACGCCGTCGGCGCGGGGGCGGCCGTCGACGTACTGGTCGTAGTCGGAGTCGGTGAACGGCCGGAAGTCCGCGCCG<br>TCCCGCTCGCGCAGGAAGGTGTCGAAGGTCGCCTTCCAGGCGGCGGCGTGCACCACCGCCGTCCTGGTGACCACCCCGT<br>CGAGGTCGAAGAGGCAGGCCTGGATGTCGTCGGGAAGTCCGAGCTGCGTCGTCATACCCCCACAGTTCCCCGCCGGGAC<br>GGGTCCGATCAGGTGACACACTGTTCGGT
SCO0763	CACGGTCAGCACCAGGGCCGAGGGGGCGTAGAGCGACGCGGGGGCGGTGGCCGGGGAGGCGAGGGAGGCCCCGGCG<br>AGGGGCCCGCAGACGGCGGTGGCCGTCAGGCCCAGAGTCGCTGCCCAGCGCGCGGTGTTCCGCATTGTGTGCATCCTTC<br>CGCTCGTTTCGAGGGTGGTGCCGGTCCGACCCGGTCGGTGCCGGAGCGGCGAGCGCGAGTCTGCCGAGTCCCGCACCG<br>AAACTCATCTCGACCCCACGGGTTTCGGTAACCTTGCGTATTGAATCAGTGGCGTGAAGTCACAGAATTAGAACGTTCCAA<br>GTCCGGAACTGGGCGTTTCTTGATCGATTATTGAGAACAGAACTGGCCGAATGGCGTGATCCGCACCTTCGCCAATAGGCC<br>TGAAACACCCCCGGAACAGGTCCGTCGGCCCCGACCGCCACCCCCGGCATACTCGCGGGTATGGTGCCGGGCAGCGCGA<br>GCTCGACGACGAGAGGCGGACCGGGG
SCO0766	GTGGCGCAGGTCCAGGGTGCCGCTCCACTGTGCGTCGTCGCTTCCCGGAGGTGCGCCGTCCAGGTCGCCCGCGAGCAG<br>GTCGGCGGCGGGGCGCCCCACGACCTCGGCGGCCGGGTGGCCGAGCAGCAGGCGGGCGCCCTCGTTCCACTCGACCA<br>GGGTGCCGGAGCCGTCGACGACGGCGCGGGCCGTGGCGGCATCGTCGAACGGGTTGACCGGACTCATCGTCGCCACTC<br>CATTGCGCACACTCACGGTGATCAGCGCGTCACCTTGTTTCCAGCCTAGTGGGTACGGGCGCCCTGCGGACCCGTACTCA<br>CCGGGCGCTCGGTGCCGGACCGCCCCAGGGGTCACCGGTCCGGTCCGGGCGGCGCAGGGCAGAAAGTGCTTTCCGTGG<br>CCTTGACGGGCGCGTATGGCCCGACGTCAGAATCTGTTTGTTCGCGATCGCTTGTGAGTACTTCTGAGTTCTGATGAGCCT<br>CGATGAGTTCTGATGAGGGAGATCCGTC

SCO0791	CTCGGCGGGCGGGTCGGAGCTGGGCGCCCTGCGACGCGCGATCGACGCGGACGACGCGATCGAGGCCACCGCGAGCG<br>CCGACGACGTGGACGCGGGCAAGCCCGCCCCTGAGCCGGTCGAGCACGCCCTGGAACTGGCCGGGGTACCGGCCGAGC<br>GGGCCGTCTTCGTCGGCGACACCGTCTGGGACATGCGGGCGGGCAGCCGCGCCGGAGTGCGCTGCGTGGGCGTACTGT<br>GCGGCGGTCTCCCCCGCGCCGACCTGGAGGAGGCGGGAGCGGAGGCGGTCTACGCCGACCCGGCCGACCTGCTGGCCT<br>CGCTGCGTGGGAGCCCCCTGGCATGAGGCACCGGTTCCTGCCCCGCACGCCCCGGCCGCGGTTCCCTCGCCCGCCCCG<br>CCGCCCGGGCACCGAGACGGGTCCGCCGCTCGCGCGGACGTCCGGCACCGTATGAGCGCTGTGACACACACCCTCCGG<br>GAGGCACTCCGGGGACGGCGGCCCGCGGCCGCG
SCO0828	CGTAGACGGCCGATCCCGTGGCCCGGGGGCCGGACGGTTCGTCCGGCCCCTACGGGCGCGGGTCGACCCGCCGCCAG<br>GCGGGCCGCGAGTCCGCGCCCCGGCGGCCGGCCGCCGCGGCGCAGCGCGGGCAGACCCGGCGGACGGCGTCGGCGG<br>CGCCCCCGGACTCGGGCAGCACGTCGGTCCAGGCGACCGCCGGGAACCGGGAGAGCCGCGAGCGGTGGAGGGACAGG<br>CCGCACACGGTCTCGTTCCTGCCCGGCTCCCGGGCGTGCACCTCGCCGGCCGGCAGCCAGCGGCGGTCGTCCTCGTCC<br>CACCAGCGGCCCGAGGCCGCCACCTCGTAGCGCCGGGTCCTGGCCATGGCTCCCGGGTGCCCCGGAGGGACCGCGCCC<br>ACACGCGCGTACCTCCTCCTGTGTGATCACTCCGCAAGCCCTGCCCGTTCAGGGCGTTTTGTCTGCTTAACATGCGGGGTC<br>CGCGCATCGCCCCGCTGTACGAGAGGACACCAG
SCO0829	TCAAGGAGGACTTCGACGACCCGGCGTCCGCCACGGTCGGCACCGAGGTCACCTGCACCTCGGTGGCCAGCGGCCGGG<br>ACGGCGTGGAGAAGCCCGCCAACTGGGACCTGTTCATGCAGGCCAACCCGCACATGAAGTTCTACAACGGCAAGCGCGG<br>CTACGTCCGGGCCGAACTCGGCCGGCAGGCCACCCGCCTCGACTTCAAGACGGTCTCCGCCATCAGCACGCCCGGCGCC<br>GCGGTGACCACGGCCGCGTCCTTCGTCACCGAGGCGGGCGAGCCGGGCCTGAAGCCCGCCTGATCCGGGCCCGCCGCG<br>CCGGGAGGGCGTCCGCGTACTGCACATGGCACCCCCGGCGTCCCCCATCGCGCGAAACAGTCCGGGGTCGGGCTTCGC<br>CGCGGATCTTTGCGGCAAGGCCTGTGACTTCGCCGCAGGCGCACCGTGATCCACGGTCGCCCACCGAACCCCCGCTGTC<br>TCGCACAGCGCCCGAAGGAGGCACATCGC
SCO0900	GATGGCGCAGCCGCCGGGGCTGCCGGAGCAGCTGATCGGCGCCGCGCCGGACGCGTTCTTCGGTCACTTCCTCGACGT<br>CTGGACCCGGGACCCGGCCGCCCTGCCCGCCGACGTCCGCGCCGCCTACCTGAAGGCGTCCCGCGAGGCGGTGCCGTC<br>CATCGTGGCCGACTACCGGGCCTCGGCCGGCGTCGACGTGGACCACGACCGGGCGGACCGGGAGAACGGCAACCGGCT<br>CCGGATGCCGGTGACCGTGCTCCAGCAGGACTGGGGCGCCGCTCTCGGCTACGACGCCGCCGCGCTGTGGCGGGCGTG<br>GGCCCCGGACCTGCGGCACGAGACCGTCGGGTGCGGCCACTTCATGGCCGAGGAGGCGCCGGACGAGGTCGCCGGGG<br>CACTGCGCGAGCTGCTCACCCGGTAGCACCCGCCTCGGGGCGTGTGCACAACGGTCGGTTGTACGTGCCACCCGCCCGG<br>TTGTTTGATCCGCCCGGCCCCGGCTCGCTTGG

SCO0916	TCACGCCCCCGGTGCCGACGCGGATACGGCTCGTGGCCCCGGCCACGGCGGCCGCGAGCACGGTCGGCGCCGAGCCG<br>GCCACTCCGGGCACGCCGTGGTGCTCCGCCACCCAGAACCGGTGGAACCCGAGCCGCTCCGCCTCCCGGGCCAGCCGC<br>ACGGTGTCCCGCAGTGCCTCCCCGGCACTGCGGCCCGCCCGGATGCGGGAGCGGTCGAGGACGGAGAAGCGGGTGTCG<br>GCGAGTGCAGAGGTCACACCGTCTTCAACGCCCGCACGCCGCACAGATTCCCGGCGGCGCGCCCTGGCTAGGGGGTGTC<br>GTTTGGATCTTGCCGGGGGTAGCGGGGTCCGGCACGCGCATCTGCGGCGTTGTCGTCGGTTGCCGGGGCTTCGCCCTGT<br>CGCCCTCCTCCGCCTTGCAGCTGCACGCACCAGACCCGGCTCGGGCCGGCCACAAGGCACCGTCGGCCGCGGCCGGCC<br>TGATCCGAACGACACCCCCTAGGGTGGGCGG
SCO0945	GCGCGGCGACGACGACGTCGCGCTGCTGGTGCTGCGCCGGCACGGCCTGGGCGCCCCGCGGACCTTCGGCCGGGTCCA<br>GCAGCACGTCTCCCCGGGCGACCCTGAAGGCCTCACCGAGGCCCGGCACATGATCCGCGCCGCCGTCCGCTCCTGGGGA<br>GCCAAGGGGCAGAGCGACGAGATCGAACTGGTCGCCGACGAGCTGATGACCAACGCCCTTATGCACACCGAGGGCTCCG<br>CCGTCGTCACCCTGCGGCTGCTCACCGGAACCGACCGGCGGCTGCGGGTCGAGGTCGAGGACTCCTCCAGCGCCCTGCC<br>GCGCCGCCGGGAGGCCGGGGACGACGGGGTGTCCGGACGGGGGCTGCTCCTGGTCGACACGCTCGCCGACGCGTGGG<br>GAGTGGAGGCGCGCGGCGGAGGCAAGGTCGTCTGGGCGGAGTTCGTGGTGGCGCACGGGGCGGGCTGAACCCGCTGG<br>CGCCGCCGGTGGCCGGTGGCACCCTGGACGT
SCO0964	CACGGCCTTCCCCGACCTGCCCGCCGCACCGCTCGCCGGGAAGACGGTCATCGACACGTGCAACTACGGCCCCGAGCGT<br>GACGGGCACATCCCCGAGCTCGACGGCACGTCGCTCACCTCGAGCGAGCTGCTGCTGCGATACGTCCCGGGCGCCATGC<br>TCGTGAAGGGTTTCAACAACATCTACTTCAAGCACCTGCTGTCGCTCGCCCGCCCGGCGGGGGCGACCGACCGCTCCTAC<br>CTGCCGATCGCCGGGGACCTGGCGCCGGCGAAGGCGGCAGCCACCGAGTTCATCGATTCCATCGGGTACGGCGTGGTG<br>GACGCGGGGCTCCTGGCCGACAGCTGGCGGCAGGCGACGGGCACACCGGTGTGGGGAACCCCGTACGGGCCGCCCTC<br>GAACGAGATGGGCCGGCCGACCGGCCCGGGCGCCATCCGCGCGGCACTGGCCACCGCAACGCGATAGTCGCAACTCGG<br>CGCGGGCCGCAGTACGGCCAGTCGCGATC
SCO1171	GTGCCCGGGGGACGGCCTGGCAGCAGACGGTACGGCGGCTGTCCGGGCGCCCGGTCCAGATCCCGGAGGCCAGGGAG<br>CTGGTCGCGCTCGGTGCCGCGGCGCAGGCGGCCGGTCTGCTGACCGGCGAGGACGCGGCAGCGGTCGCCCGACGCTG<br>GAACACGGCCGCCGGGCCGGTACTCGACGCCGTGGAACGGGACGAGGCGACGCTGAACCGGATCACCGGGGTACTCTC<br>CGACGCGGCACCGCTGCTGGAACGGGACGCGGCCTCCCGCTGAGGGCGCGCCCGCACGGGCGGGCCACCGGCGGACC<br>CTCCGCCCTCCGCTCCCCGCGGAACCGCCGCGGACGGGTCGCCCAGCAGACCGGGACGTGACGAGGGGCATCTCAGGC<br>ATCTCCCGTCGTCTCCGGCTTCGGACGACCCGCACGTGGCCCGACCGCGCCCCGTGGCGGCCGGCCGGGACCGGACCG<br>GACCGAGCGACGAGAGACCAAGGACTGACGGAGGC

SCO1446	GCTGGTGGCGGAGTCGGGGATCGGGTGGACGGTGCTGCGGGCCACCCAGTTCCACGACCTGGTGGTGCAGATGCTCCG<br>GGCCCTGGCCAAGCTGCCGGTCGTGGTGCTGCCCGCCCACGCCTCGGACCAGCCGGTGGAGGTGGCGGAGGTCGCAGA<br>CCGGCTCGCCGAACTGGCCCAGGGGGCGCCCGCCGGACGGGTGCCGGACATGGGCGGTCCCGAGGTGCGCACCTTCGC<br>GTCGCTGGCCCGCGCCTATCTGGGCGCGACGGGACGCCGGCGGGCCGTGGTGAACGTGCCGCTCGCGGGGAGGACTTA<br>CCGCGCGTTCCGCGCGGGCGGGCATCTCGCGCCCGAACACGCGGTCGGCGAGGGCACGTTCGAGGAGTACCTGGCGGG<br>GCGGTTCGGCCACAGGGAGGGCGGGCGGCGGCGCGGATGAGCTGACGGGCGGGGCCGGGGGCGGGGGCGGGGCCG<br>GAGCTGCTCGCGGTCGCCCCGGTGGTGCTGGGGCT
SCO1602	TCAGTAGCCGTTCGCGACACCAGCGCATTTCAGGGGCCTCGATCTGTCTATAGAGCTCTGGGGCTCGCGATGAACAAGAG<br>GTCGACCCTGGAATGGGCTCGCGCCGTGACGACAACGTCTGGGCGGCCATTGTGAGTTTCGTGGTGGTCACGCAGGCAC<br>CGGGCCGTGCCGCTCTTGACAGAGGCCTGGAACGGCCGGGGGAAACGGAGGCATCAGCCCGGCGCCCGCGTCACCCCA<br>CGAAACTCACGAGTACGCGCATGTTCGCGGTGGTGCGCAGGCTCCGGGCTGTCGCTCTATCGGGAGGCCTCACGGCCA<br>TGGTCGGATCAGACCTTGCCCGGCACCCACGCACCACCGGTCATCGGTGGGCAGCGGCGCCGGTCTCAGAGAGAGCCTC<br>GGGGACTGGAGAGGGCCGAGGCGTCCGCTGCCCGTGCAAGCGTCTCTTCGCATCCTTGCGGGGTCCGCGACGAGCTGCT<br>TACACCCTGGATTAGGGTCGGGGACC
SCO1609	CCCCGACACCGGCGGCCCGGCGGCATCCGGTCGGGGGGTCCCGTCCGCGCCCGGCGTGGGCGCGGACGGCGGTCCGG<br>GGTCCGTGCGCGGCGGCGCCTCGGGACGGGGCGGGATCTCGGGGTGGTGGGGCGGGGTCTGCGGGCGGGGCGGCGT<br>TCGCAGGTGCGGCGGAAGGGAGGCGCGGCGCGCTTCCGTGCTCATGCACCCCCCGTTTCCCCGGGCCCGGCCCGTTTCT<br>CCGACACGGGCCGGAGACGGTGGTCCTGACCGTGGTCTCCTGCCCGGCCCGGCGCGGAACCGATCCGTCCCGGGCACG<br>CATACCCGCTCGGGAGGACCGCCATCCCGGCGCGGGCTGCCGGGGCCCTGCCGCCGGGGCCGGATCCGCCCGTGCGT<br>GCGCGTCACTCTACGGCGTGACCCCGCCCGACGGGGAACCAGTCCGCGCGCCCGCGGCATTGGCCCGGATCGTCCCCCT<br>ACCCTGCGGTAATCCTGTCTGGCAGGCTGCCGTC
SCO1694	CCGAACCCGGCCCGGGAGCAGAGATGGTCCCAGGTCGCCCTGTGCCCATAAGGGGCGAATCGCTCAAGGCACTTGCGCA<br>GCGAATAGCGCCGCAGCGCCAGGTCGCACCGTGGATCGCGGACCTGTCTCGCCAGACTTCGGAAACCGGCCATCGCCCT<br>GCACCTCCGTCACACCTTCACCTGTACTCGGGTCACTTCGGCGTCGCCGTACGGACGTCGCCGAATAGACGTATCGACAA<br>CCGATTCGGCTCCATCCGATTTCCGATGCCCCCCATAAGCCGATCCACCCGCTCCCAAAAAGTGACGCATGTTCACCTTCC<br>CACTCGGGGATACCGGCGGTAACGTCCCGCCACACCCCCGTCGGGAGGAGCTGCCATGCCACGGCGCACCCCACGCAAC<br>GCCCTCGACAGACTGAGAACTCCCCGCGGCTTCCACGGGTTCCTGAAGACCGCATCCGTATGCGCCCTCGTTGCCGGACT<br>TTTGTCACCGCTTTCCCCGGCG

SCO1704

CGAGGCCGCCGCCCGGCGTCCGCGCACCCTGCGGGCGTCGAGCGCCGGTTCGGCGATGGCCCGGGGGCGCACCTGCT
ACGACCATCTCGCCGGACGGCTCGGCATCGCGGTCACCGACGCGCTGACCGGACGCGGGCTGCTGCGCCAGGACACCG
GCTTCGCACTCACGGACGCGGGGCTCGGCTGGTTCGACTCCGCCGGGATCCCGCTGCGGCCGACCGGGCGCCGGCCAC
TGGCCCGGGCCTGCCTCGACTGGACGGAACGCCGTCCCCATCTCGCGGGCGTGGCGGGCGCCGCCCTGTGCCGGCACG
CCCTGGACACCGGCTGGTGCGTGCGCATCGGCTCGGAGCGGGCGGTGAGGGTGACACCGGCGGGCGAGGACGCGCTGT
CCCAGCTGCTGGACATCGACCCGGCGGCGCTGCGCTGAGCGCCGCCCCCGGCACGCTCACCGTCCGGAATCCGCCAGC
ACGCACCCGTTCTCGCCACCTAGCCTCTGGAGC

SCO1737

CGCCCGCACCCCGTCCTCACCGAGCACGTCGGCCCACGCGCCCACGGCCTCCGCGGCCGCCGCCTCGGCCGCCCGCGT
GCAGGCCCACCCCGCCTCCGTCAGCACGACGAGCCGGGCGCGCGCGTCGACGGGATGCGGCCGGCGCTCGGCGTACCC
CTTGCGGACCAGCTCGTCGACGAGCTGACTGGCGGCCTGCTTGGTCACTCCGAGGTGCACGGCCAGGTCGGTGACCGTC
GCACCGTCCGGGGCGAGCCGGGCGAAGGCGAACCCGTGCGCGGGCCGCACACCCTCGAAGCCGCGGGCCACGACCCCT
TCGTTGATGCGCTGCGTGAGTCCGCCGGCCGCGGCGAGCAGGGCGGCGGTCAGGGCCATGGCGTCGGAGTTCTGCACG
GGGGCATTGAAACACTCTTGACACTTTGGTCAAGCAGCTTGACCATAAGAGCCACAGTATTGGTCAAGCTGCTTGACCATG
TCGGCCATATGTCCAGAGGAGCCCCGTC

SCO1779

GCGCGCAGGTCGTGGTCGTCACGCACCTGCCGCAGGTGGCCGCGTTCGCCGACCGGCAGCTACTGGTGGAGAAGACGA
ACGACGGCTCGGTGACCCGCTCCGGCGTGAAGGTCCTGGAGGGCGAGGAACGCGTCCGGGAACTCTCCCGGATGCTCG
CCGGCCAGGAGGACTCGGAGACGGCCCGCGCCCACGCGGAGGAACTGCTGGAAACGGCCCGGGCCGACCGGTAGGGAC
TCCGGGGGCCGGGCCGACCGGCAGGGACTCCGGGGGCCGGGCGGGCGTGCTTCGGCGGTCTCCCGGACGCACGCCCC
GGGTGGCCGGTGCGACGGCGTGACGTTCACTCGTACGAGTGAGGTGGTTCCGTGTCGTTGTCCCGCCGGTCGTCCGGTC
GTGGCGGTCGTCGTTCCCGGAACACCCGTGCGGCCTGGCATCCTTGACGGAGCGGCGCACGGGAAACCGCCCGGCGCA
CCCCGTCCGCACCATCCTTCTGTACGTTCTTC

SCO1854

CTGCGCGACCTCCGGGATGGTCAGCTCGCGCTGCGCGAGCGCGGCCACGGCCGAGAACGCGGAGACGCCGGGCACCAC
CTCGGTCGCGACACCGATCTCCGCGCACCGGTCGAGCTGCTCCTGCGTGCCGCCCCACAGCGCCGGGTCGCCGGAGTG
GATGCGAGCCACCCGCAGCCCCTCCGCCCGGGCCCGCCGGTACACGGCGACGACGTCCTCCAGCGACATCGTCGCCGA
GTCCAGCACCTCGGCGCCCTCGCGCGCGTGCTGGAGCACCTCCGCCTGGACGAGGCTCGCCGCCCAGATCACCACGTCC
GCCTCGGCGATCGCCCGCGCCGCACGGAACGTCAGCAGGTCGGCGGCGCCGGGACCGGCACCGACGAAGGTCACCTTG
GCCTCGGAATCCATCGTTTTCGGTCCTCTCCTGTGCATCCTGCGCATATTTACTGCGGTTGCTGCTGTCGTACGCGCGTGA
ACGCGGGGTGATCGGATACCAAGGGCCC

SCO1892 GCACCGGCGACCTCGCCGGGCAGGTCGATGCCGCCGGCCCGGGTCATGCCGGTCCACGCCTCGGGGTCGCGCCACGCC
TCGGCGAGTTCGCCGAGTGCGGCGGCGAGCTCCTCGCGCCAGCCGGGCCCGACGTCCGGCACGGACGCCTCGGGGCTG
GTGTCCGTGGTGGGCCCGAGATCCTTGCGCGCGGCGTCGCGGAAGGCGACGGCGAGACCGGTCAGGTGGCCGAGCAGG
TTGCGCACCGCCAGACCGGGGCAGGGCGTCGGGTCCGCGAGCCGGTCCTCGCGCACGCCGTCGGCGAGGCGGGTGAG
GACCCGCGTCTGCGGTCCCAGGTCGAGGGTGGTGGTCTCGGCGGTCATCGGCTGCTCCTCGGGATGTGCGTGGCCGGTT
CTCTCGGAAGGTTGACCGGTCGCACCCGCGAAACTCATCGGCCGCCGCGGCGAGTACTGGGGCACCTGCCCGATGCCCG
CACCCGGGCCTTAGGCCCAGGGTGACCGGGT

SCO1896 CCCGCGCCCGCCAGGACGGGCACCCGGCCCGCGCTCTCCTCGACGGCCGCGCGGACGCAGGCCGCGAACTCCTCGGGC
GTGAGGGCGTGGAACTCGCCGGTGCCGCAGCAGGCGAAGACCGCGGCGGCACCGGCCTCGACCCCGCGGCGCACATGG
GTGCGGTAGACGTCGAGGTCGAGTCCGCCGTCGGGTCCGTACGCCGTGACGGGGAAGAACAGCGGGCCGCTGGGCACG
GTGAGCCGGGCGGCGAGAGGGGCTGAGGTCACGGGCGCTCCCCATTCCAGGTGCAGGGTCGATTCACGGACGTCATCAT
ATTCATGACCTGCGTCTACATTTCTGAACGCACCACGCTAAGCCGCCCCGCGAACACCGTCAAGCAGGCGCCCTCTTGACG
CCTGACCACCGGCTCCTTAGCGTGTCCACGCATCTGAACGTGAGGCACACACCGGCACGCACGCGTACGTCTGCCGCTTC
CACAGACCGACCTAGGAGTCCCGAGG

SCO1954 GGTCCTGGAAGTAGTTCGCCAGGCGCTGGCGCAGGCTCTTCGCCTTTCCGACGTAGATCACCCGGCGGTGCTCGTCACG
GAACCTGTACACCCCCGGGGAGTCCGGGATCTCTCCCGGCCTGGGGCGGTAGCTGGAGGGGTCGGCCATGACTCACACC
CTACTGGCGCGGACCGACACTCCGGCGGGCCTGTGGACGACGGGCCGGACCCCTGTGGACAGCCCGCGGGAGAGGCCG
GGGAAGACGGTACGGGGCCGGCCGGTCACGGCGGGTGTCCTCCGGGACCGCGGGCCCGTGCGTTCTCGGCCAGGAGC
GGACGGCGGTCGGGCGCCAGGTGTTGTCGGGACTAGGTCGACACGCTTCAATGCGGGGGGACTCGGGGCCCTGAAGCG
CGGCGTACGGATGTGAAGACCTCTGCGTCGCGAGCGGGGGCGGGCGGCCCCGGTCGATCCGTGTCGACGGCCTGACCA
GCCGCCGTGAACTTCACAGAAGGGCCCCTGC

SCO1990 GGCGACCGGAGCACCTACGCGCTTGCGGATCAGCCGCTTCACGACGACGACCCGTTCCCGGCCCTCCTGTCGCAGGCGC
ACCTCGTCGCCGACGCGGAGCGAGTACGCGGGCTTCACCCGCTCGCCGTTCACCCGGACATGGCCGCCGCGGCAGGCG
GTCGCCCCCAGGGAACGGGTCTTGATCAGGCGGACGGCCCAGATCCAGCTGTCCACGCGGACGCTCTCCCCGTGCTGCG
GCCCGGCCGCCTCGGCGGCGGCGATCGCCGCCGCCGTCTTCGGGTCCGGTGTCGCCGTCTTCGGGTCCGGTGTCCCGG
CCGCGGCAGCGGCGCCGTCCCGCGGTGCGGCCACCGGGCCGCTCCCGCCCTCGTCATGCTCGGAAGCCATGGTCCGAC
CCTAACCGCCCGGCACGGCGCGGCGGGCCGCAATTTCGGCGACCGACGACGGCCACCACGAACAGGGGCACGAGAGGG
TTGGGCACGAGAGGGTCTTACGGTGGAGGC

SCO2000	TGCCCTTCGCGGACGGCTCGTTCGACCTGGCGTGCTCGGCGTACGGGGCGCTGCCCTTCGTCGCCGACCCGCGGCTGGT<br>GCTGCGGGAGGTGCGCCGCGTGCTGCGGCCCGGCGGCCGCTTCGTCTTCTCGGTCACGCACCCGCTGCGCTGGGCGTT<br>CCCGGACGAGCCGGGCCCCGAGGGGCTGTCGGTCTCCGGCTCGTACTTCGACCGGACGCCGTACGTCGAGCAGGACGA<br>CGAGGGCCTCGCGGTGTACGTCGAGCACCACAGGACGCTCGGTGACCGGGTGCGGGACGTGGTGACCTCCGGGTTCCG<br>GCTGGTGGACCTGGTGGAGCCGGAGTGGCCGGACTGGAACACCTCGGAGTGGGGCGGCTGGTCGCCGCTGCGCGGGCA<br>TCTGATCCCGGGGACGGCGATCTTCGTCTGCGAGCGCGACTGAGCCGTACCGGGAGGCGGGTCCACGCGCGCGTGGAG<br>GGCGCTTTCGCGGACGTACGACACTGGGGGC
SCO2040	GACGGCGCCCGGGCCCGCCTCCCGCCCCCGCGTCCCGACCCGCGTCCACCCACTGGAGCACCCCCGCTCACGGCGCGT<br>GCCCCCGTGCGGGACAATGGGTGCGAGCGTCCTGCTCAGACATGGCACACAGCAACGAGGAGCAAGCAATGGCGGGCAG<br>CAGCCACGGTCACACCCCGGCCGCCTGGACCGGTGTCATCATCGCCTTCATCGGTTTCTGCATCGCGGGCATGTTCATGG<br>TGGCGGCCCAGCCGTGGGGCTTCTGGGCCGGCATGGCGGTCGTGCTCCTCGGTGGTGTCGTCGGCTACATCATGAAGAT<br>GATGGGCCTCGGCCAGCCGAAGCAGAACCACCCCGTGCACCAGGTCGCGGGCGAGCGCGCCGAGGCCGGCGCCAAGGG<br>CTGACCGGGCGCGGCGGTCACGGACCTCCGAAGGGGCGGACCGGCACCACCGTGCCGGGACCGCCCCTCACGTGCGTG<br>CGGGGGCCGCCGGAGGCAGAATGCGGGA
SCO2042	TCCGTCGAGGTCGTCGACTTCATGTCCGTCGAGCGGTACTCGCACGTCATGCACATCGTCTCGACGGTCACCGGCCGGGT<br>CGCCCCGGGCCGCACCGCCTTCGACGTCCTCACCGCCTGCTTCCCGGCCGGCACCCTCTCCGGCGCGCCCAAGCCGCGC<br>GCCCTGCAGATCATCGACGAACTCGAGCCCTCCCGGCGCGGCCTGTACGGCGGCTGCGTCGGCTACCTCGACTTCGCCG<br>GCGACTCCGACACCGCCATCGCCATCCGCACCGCCCTGCTGCGCGACGGCACCGCCTACGTCCAGGCGGGCGCGGGCG<br>TCGTCGCCGACTCCGACCCGGTCGCCGAGGACACCGAGTGCCGCAACAAGGCGGCGGCCGTGCTGAGGGCCGTACACA<br>CGGCCAACCGGCTGGCGCAATAGGGCCAACCGGTCGGACCGGTGGGGCGATTCTCACGCCCTCAATGGGAACCCCGGGT<br>GACGGTTCGCCCGGGGTTCGGGTGATA
SCO2062	GACCTGAAGGTGCTGCTCGGGCCGTCCTGCCTGGCCGGCTGAGCGGCATCCCGGACCACGACGAGCGAGGGCGCACCC<br>GGTTGCCGGGTGCGCCCTTCGTCGTGGCTGTCAGTTGTGGCCGAAGCGCTTGTTCCGCTTGCGGGCGGCCGCGTCGCCG<br>GGCGTCGTCAGCTGCGGGGAGTGCTGCTCCTCGCGGTCCAGTACGGCCGACTCGGCGGCCTGCGGGGAGCGCTCGGCC<br>TGCGGGTGCTTACGATCACGGTTGTTCTTGTTCTTGGCCATGGTGATCTACCTCCTGAGGGGGATCTAGGGGCCAGGGCC<br>GGAACCAGATTCACATAGGCCAACAAAGAACGCATATCGGGCAATTACCGTCCGTGACGCGGTCCGAGCGGACACGGGGT<br>GCCGAAACGCCACGCCGAAGATCGAGTTCGGGCCGTTAACCTCCGCGTGGTCGGGCAGACTCGAAGGAAGCCCGAAGCA<br>AACCTCCCGGAAAGAGGGTGGACCGC

SCO2074	GCTGTTCCCAAGGCCCGCGGCACCGCCGCGGTCCCCGGCGACCTCGCGGTACGCCCCGGCGAGGAACCCTGGACCCCG<br>CAGGAGGTCGAGGAGGCGCGCGGCGAGCTTCAGTCCGAGGCCGACCGGCTGCGCACCGAGATCGACACCTCCGAGCGG<br>TCGCTGCAGGGCATGATGCGGGACTCCGGCGACGGCGCGGGCGACGACGAGGCGGACACAGGCAGCAAGAACATCACG<br>CGCGAGCACGAACTGGCGCTGGCCGCCACCGCGCGCGAGGTGCTCAGCCAGACCGAGCGTGCCCTGGACCGCCTGGAC<br>GCGGGCACCTACGGCCTGTGCGAGAACTGCGGCAACCCGATCGGCAAGGCGCGGATGCAGGCGTTCCCGCGGGCCACC<br>CTCTGCGTCGAGTGCAAGCAGAAGCAGGAGCGCCGGTACTGAGCCGCAGCCGGGACGGGAAGTGTGCCGTACCCTCGTG<br>CTCAGTCAAGGACCTAGGCTGAGGGACTCAC
SCO2106	GCATCCCGGCCGCCACCTGGGCCGCCCCGCCGCCCGGCTCGGGGTCCACGAGGGCGGTGACCAGCCCGCGCCGCGCG<br>GCCCGCCAGGCGGTGACGAGCCCGATGATCCCGCCGCCGACGACGAGCACGTCGGGCGTACGGGACGTACGAGGTGTG<br>TGCGGAGGCGTGTGTGATGGAGACGACATGGGCGTCCAGCCCCTCCCTTCGCCGGCATGACCCGGATCAGGTTCGTACG<br>GTCGGAGGCCGCCAGCCTCCCTCTCAGCCCGGTGCGTCCGGGCTCCCGCGAGTGCCTTGCTCCGGCCACCCTAGCCCGC<br>CGGGCGGCGCCCCTGTAAGGGAGCCTCCCGGGGCCGGGGGCGTCGCCGGGCAGCCCGCGGGGTGCGGGCACCGACCG<br>GCGAAACGCCGGGTGTCTATGGTGATCGGGTGAGCGAGCAGACGCGGGAACGGGAACAGCGGGACCGACGCGAACGGG<br>GACGGCGGCCGGCGGCGGACGCGGAGCGCCGT
SCO2173	GCAGTGACGGGGGCGGGGCCGCCCCCGCCCCCGGGCCTCAGGCCTCGGTCCCCGCCTCGGCCTCGGCATCGGCCTCC<br>GCCTCCTCGGCGTGCAGGCTCATCGGGCCGTAGATCTCGGTGGAGTCCTCGAAGAGCCGCACCTGGTCGGTGCCGCCCG<br>CCCGGAGGTCCTTCCACTGCTCCCCGATCCAGGACTCGGCGTCCCCTTGCGTGGTGAACTCCTCGGGCTGCACCGCGGG<br>CTGGACCTCCGTCCCGTCGGCCGTCTCGAACCGCCACGTCCATGCCGCCATGTACGCCTCCAAGGTGTGAGCACATGCAG<br>AGCGGAAGCCGGACCTTGATCCGTCCTCCGCCCGCAGCCTATGCGCTGAACCGGGCGCACCGTAGAAGGCCCGGGGAGA<br>AGGCCCCGCAGGGGGTCCCGGGGGCGGGAACGGTCGCGGCATCCGCCCCTCGGGGGGACTCGGGCGCGCAGGTCGCC<br>GGGTGACCGGCGAAAATCGGTGCGTGGAC
SCO2177	TCCTGGGACATCCCGTCGACACCGAACGGCGTGAACCCCGCCAGCACCAGCCCGACCAGCGCCGCGGTGAGCACCACGG<br>CCTCCACGCTCTTGCGCCCCGCCTCGCTCCAGTACACGTCGTCGAGGTGCAGGATCAGCGCGAACTCGTCCAGGACCAGC<br>CCCGCCCCGATACCGAAGACCACCGCCGCGAGAGCCGACCAGACCCCGTGCCGGCCCCCCGCGACCGCGCCGAAGCCG<br>CCGACGACGGTGAGCACGACCCCCGGGACGACGTGGTGGATGTGCACCCCGCCCGCCTTGACGTTCCCGAAGGGCCCCT<br>TGCCCGCCCGGATCAGACGGGTGATCACCCGGGTGACCGCGAACGTCAGCACGAACGCCGCCAGCGCGAGGAGCAGCG<br>GCAGCTTGCCCGGCTCCATGATGTTGCGCTGCCACCAATCTCCCATATGCGCACTTTATCCCCAGCACCCGCCCGCACCCC<br>GCCGACCGCCGGGTAGTCTGCGCCG

SCO2190	TCCACGACGAAGCCCTCCGACTCGTAGAGGCCCCGGGCGGCCGTGTTGTGGCCGAGGACCCGCAGGGTGATCCGGCGG<br>AAGCCCTCGTGCCGGGCCTCCTCGACGGCGGCCCGGACCAGTGCCCGCCCCACGCCGTGGCCGCGGGCCGCGCCGGC<br>GACGGCGAGGCCGCGGATCTGCCGGACGTGGGTGTTGGAGGCCAGCGGAGTGGGAAACCCGAGGCGGACGTAGCCGAC<br>GACGGCACCGTCGAGTTCGGCGACCAGGTGGGCGTCGGGGCCGCAGGTGTCCCGGAAGAAGGGGTCGTCCGGTCCCGG<br>TGGGGGCGAGACGGCGTGCAGGTAGGACCAGGTCTCGAGGTCGATCCGGCGCAGCGGGTGCTCGTCCTCGGACCTGGC<br>GCGGCGTATGTGCGGGGCTGGCATGGCGATCACTGTACGACGCGGTGTCGGAGACCGGTCCGGGGTTTTTCCGGGGCCA<br>CCCGGCAGGATGGCCCCCTGGAGGACGGCCAC
SCO2255	CGTCGAAGACGGAGGCGGTCATCGCGTCGTACGCGGTGAGCATGGGCCACTTCTCGCCGCGCTCCTTGGCGGCGGTGAT<br>GTCCCGGACGGTGATACGGCGGTTGATCTTGCCCCCGTACAGCGCCCTGTTCCCGTCGGCGGGCTTGGGCTGCGGCGTC<br>TGGGCAGCCGAAAGCTGCGTCATGGCAACGGCTCCTTCATGTCATCTCGAGGCGCCCTGACGGCGTCCCCGGATCCCTTC<br>CATGGTGGCACTTGTCGCGCCCCGGCGGCTAGGGGACCCCCGTCACGTACCCCGTCCGGGGTCAGAGCATGGCTCATGC<br>GGCCAAATGTGCCCGTTGTGTCACAGAGCCGCCCGTCCCGGCACAGTGCGGAACTCCGCGGGACGGCCCGTACGTCCTC<br>GTCCCCGTACGGCCGTCCAGGGCGGCGGGATCGGAACCGATCATCCCCTATGGTGCTGAGTCACAGGGGGCGACGGTGT<br>GCACAGCAGGCAGTGAGGCAACGGT
SCO2264	GGGAGAACGCGGTGCCGCCGCCCCGGGGCCGCCGCCGCTTCCGCGACCGCCTCTCCCGCCTCGCCGACCACCTGACGG<br>CACCACCACCGTCCGGCTGGTGACGACTGCCGACCGCTCCTGTTCAGCCGGCAACGCCACACCCCGGCCGAATCCCCCG<br>TCACCGTCCAGCCATCGGCCGCGCCCCCGACGACCAACCGCCCCGGCCGCCCGGCGCCCTCCGTACGGACCGCGCCCG<br>GACGCCTGCCACCGCCGTCCGGCCGCGACCGACCGCGCCCCGGCACCCGGCACCCGGCCGCCGCAAGCCCGGCCCATG<br>ACCGCCGCAGTCCCGCCCGCGGCTGCCGCCTCCCTGCCAACCGCGGCCCGGCCGGCCGGGACCGGCGTGGGCCGGTC<br>GTCCGTCGCCGCCGGGAAAACCGGTTGCCCGTGCTCGTCCCGCCGCCGGACGATGACCCCTCGTGACCGACACCTCCTC<br>CGCCGACGCTCCCGTCGGCAAGGGCGCCGAC
SCO2376	GGTCCCCGGTCTCCCTCTTCTGCCACAGCTACGGCTCGGTGGTGTGCGGCCTCGCCGCCGACATGCTCCCCTCGCGGGT<br>GACCGACATAGCGGTGGCCGGCAGTCCCGGCATGCGCGCCGAGAACGCCGCCCGCCTGGACACCTCCGCCCGCGTGTG<br>GGCGATGCGGGACGCCGACGACTGGATCCAGGACGTGCCGTACCTGGAGGTCGGCGGCCTCGGGCACGGCGCCGATCC<br>GGTGTCCGCCGGGTTCGGGGCGCGAGTGCTGTCCGCGCGGGACGCCCAGGGCCACAGCGGCTACTTCGTGCCGGGCAC<br>GGACAGCCTGCGGAACTTCGCGGGGATCGGTGTTGGCGCATACCGCACGGTCGCCTGCGCCGGCGAAGACGACACATGC<br>CGGGCGGGTTTGTCCGCGGCGACGGCGGCCGGACGCGCGTAGAGGCGCAGAAACTCCGGTTTGCACGGGGAGGGGAC<br>GGGAAGGCTCGTGCCGCATACGATGAGCCGC

SCO2385

GGTCGCGAAGGCGATCTCCCGGGCGTACACGAGCAGCGCCTCGCGCAGCACCGACTCGTCGCCGGGAGCCGCCACCTC
GTCGATCGCGGACTCCATCACCTCGATGGTGGTCCGCACCATCTCCACGGTCTGCCGCAGCGTGATCGCCCGGGTCAGCT
CGCGCGGGGCGGTCCCGAAGACGTCGGTGGAGATGGCCTGGGGCGCGTCCGGCTGCCTAAACCACTCGGTGAACGCGG
CGATGCCGGCCTGGGCGACCAGCCCGATCCAGGAACGGTTCTCCGGTGGCATGGCCCGGTACCACGGCAGCGTCTCGTC
CATGCGCGCGATGGCCTGCGCGGCGAGCGAACCGGAGGACTGCTCCAGCCGCTTCAGGGTCGCGGTATGCGGGTGGGC
GGGCTTGGCGGGGGCGTCGGGCTTACCGGTTTCGGGTTCGGGCACGGGGACAAGACTGCCTTATCGGGCCGGGCGGGT
GTGCCGCCGGGGCTACCGTGAACGACGTG

SCO2409

CAGCGAGATCAAGGGCCAGGTGGCCAAGGCCGTCGACCGCCTCTTCTCCTACGACCACGCCGACCCCGGCGCCCTCGAC
AAGGCGGCGCGGGACCTCCTGACGGGCAAGGCCGCCGACCAGCACCGCGACCTGCTCGCCGACGTCCGCGCACGGGCC
GACGAGCAGAAGGCGGTGATCACCACGACGGTCACCGAGAGCGCCGTCGAACGCCTCGACGGCGACCGGGCCCGGGTC
CTCGTCTACGCCGACCAGAGCAGTGTCGCCACCACCGGGAAGAAGGCCGCGACGGACAAGGAGAGCAAGACGGACGGG
GCGGCCGCGCAGGACGAGGGCGTGTACGCGGCGGCCATGCTCGCCGTCGACGTCGTGCACCGCGACGGACGCTGGCTG
GTCTCCGCCCTCGACACCTTCGGCCGCTGAGCCGCACGACCGACCGACCGCCCTGGTGGGCCACCTCCCCGGCCCCGCA
TCTCCGACGGATCCGACAGGAGTGACGACC

SCO2410

CTGGGCCGGGCAGGACTGGTACCGGGCGGCCCACGACGAGCAGGCCGCGTACGCCGCACAGCGGGACGCGGCACTCTC
CGCCGGCGAGCAGGCCGTGCAGAACCTCAACACGCTCGACCACCGTGAGCTGAAGCGCGGCCTGGACCTGTGGGAGTCG
TCCACCACCGGCGAACTGCACGAACAACTCGCCTCCGGGCGGGAGGAGTTCACCGCTCAGATGAAGGAGGCGAAGACGG
TCAGCACGGCCCGGGTGCTGTCCGGCGCCGTCACCGAACTCGACGACCGCGCCGGACGCGCCCGGGTGCTGGTCGCCC
TGCGCGTCACCGTCGAGGCGGCCGACGGAGCGAAGAGCGACAAGGACAGCCGGATGCTCGGCGAACTCACCCGGACCG
ACGGCCGGTGGAAGCTGAGCGCCCTGGGCCAGGCGCCCGTCGGCGGGACGTCGACCGGCTGAGCCGGTCCGTACCGCG
AGAGCCGGCCGCAGCGAGGAGGACAGCACC

SCO2441

CGCACCGCGTGCGTGGAGTCGACCTTGGCGATGTAGATGACGTCGGTGTCCTCCAGGATCGCCACGTGCACCGTCTCGTC
GCAGGTCTCGGCGACGGTCCGGGCGACCTGTTGGCCCTCCGCGGCGAGGTCGAGGTACTCGGCGTAGCGGGCGCCGAG
CTGGTACGGGCGCACGCCGAGCCGGTACCGTCCGGACTGCCCGGCGACCGGGACGATGTACTTGCGGGCGGCGAGCGT
GGTGACCAGCTCGTGCACGGTGGTGCGCGGCAGCTGGAGCCTGCGCACGATGTCGGGGGCGGAGAGCGTGCCGTCCCC
GTCGAGGAAGAGCTCCAGTATGTCGAGAGCCCGGGTCACGGCCGGCACGAGGCGTCCCATGTCCAGCCCCCTCCCTAGA
TATGTTCGAGATTTCAACAAGCGATCGGAATAACGAACACAGGCTACTCATACCGCGTTTGCCTGGGCAATGGGCGTGCGG
CCGCCCCATGGTCCACTGCGTCCGCG

SCO2468

CGCGCGCACGGCGGCGGCATCGTCGTCGACGACCACCTGCGCACCTCCGACCCCGACATCTTCGCGGCCGGCGACGTG
GCCTCCTTCCACCACGCCCTCTTCGACACCAGCCTGCGCGTGGAGCACTGGGCCAACGCCCTGAACGGCGGTCCGGCCG
CCGCCCGCGCGATGCTCGGCAGGGGCCTCGCCCACGACCGCGTGCCCTACTTCTTCACCGACCAGTACGACCTGGGCAT
GGAGTACTCCGGCTGGGCGCCGGCCGGCTCGTACGACCAGGTGGTGATCCGCGGGGACGCGGCGAAGCGCGAGTTCAT
CGCCTTCTGGGTGAAGGAGGGCCGGGTGCTGGCCGGGATGAACGTCAACGTGTGGGACGTCACGGAGCCGATCCAGCA
GCTGATCCGCTCGAAGACCCGGGTGGACACGGAGGACCTGGCGAACCCGCACGTATCCCTCGAAAGCCTCGTCGCATAG
TTGTCGGTCCGCCCCCGTAGACTTCACGC

SCO2536

CCGGATACGGCATCCACGTGTCGATCAGGCGCCGCAGCCCCTCGCACATGTCCCGCTTCGACGGCTCCACCTCGGCGGC
GGGCGGGTTCAGCCCCGCCAGCACGTACAGATGGCGGCGCTCGGTGTTGCTGAGCCGCAGCACCCGGCCGACCGCGTC
CAGCACCTGCGGGGAGACGGAGATGTCGCGGCCCTGCTCCAGCCACTGGTACCAGGACGCGCCCACCCCGGCGAGCAC
GGCCACCTCCTCGCGGCGCAGCCCCGGCGTGCGGCGCCGCGCCCCGCCGTCCGGCAGGCCGACCTCGGCCGGGGACA
CCCGCGCCCGGCGGCTCATCAAGAACTCCCGCAGCTCACTGCGGCGCCGGTCCCGGCCCGCGGTCCCGGCCCTCGTGAT
CACGTCCGGCACGTACGCACTCCCCCCGTAGCCAACCCGCCGGTGCCTGGTGGTGGCACCACCAGAACAATCGGCGTCT
CCCCACAACTATTCCGGGGGCCCGAGGCTC

SCO2551

TCGCAGTTGCCCAGCCCCTTCACCCGGAACCACTCCTTGCCGTCCACCTTCGCGGTCAGGTTCCCGCGCACGCAGGCGCC
GTTGACGACCTTGGTGACCTTGCCGTCGACGGTGATGTCCTGGCCGGCGTCCGTCCTGCCCTCGCAGTGCACGGAGGCG
ACCGCGTTCTCGCTCGCCGAGGCCGTGGTGCCCTTGTCGCCGTAGGAGGCGGTGCAGGTGAGCCAGCGCACCTCCGCCT
TCTGCCGCTGGAGTTCCCGGGTCACGGTCTGGTCGGTCGTGTAGGCGACCGTCGCGGAGCCGAGTCCGCCCGGCTCGCA
CGCGACGAGCCCGCCGACGGCGACGGCCGAGCCGACGACCACCGGAGCCATGCGCCGAATCCGCCTCAATGCCCCCAT
GGAGGGCAGCCTGCCACCGCCCGGGGGCCGGCGGTAGGGCGCATACGGCCACTCCTCACTACCGTGGGCGCCATGGGC
AACGACCGCAGGCAGCGCAGGCTCGTC

SCO2564

AGTACCTCGGACTCGGCAACGGCATGGAGGGCGAGACGCGGGCGGCCTGCCGCGCCACCACCGAGCTGCACCGCGACG
ACTTCACGGTGAGCTGGCAGACGATGCTGACCCGCGGCATCGCCGTGGTGGGCCCCAGCATCCGCATCGACCTCGACGT
GCAGACGGTGCCCAAGGGCTGACCGTGCCCGGGGCTGACCGACAATGGCATCCGTGAGCGACGTGAGACATGTACTGGT
GCTGCCCGACCGCGACGCCGCGGAGCAGGCCGCCCAGGAACTCGGCGAGCGGTTCGGGACCGACGAGGAACCGCGGAT
CCTGAGGGACGCCCTGGCCGGCGAGGACGACGCCGAGGACGCCCAGTGGCTCGTCCTCCTGCACGACGAGCGGGAAGC
GCTGGACCCCGGTGAACTGGACGCGTTCGCGGGGGAGTGGGAGGGCTGGCGCGAGGAGCCCTAAGCCGCGGGGCCCG
TCCGGCAGCGGCCCGTCCGGCGTTGTCAGTC

SCO2602 CAGGATCAGACACATCCAACCGAAGTGACACCCCCAGCGGGCCAGGCGATCATCCGCGTCAGCGGATCACTTGGATGCTG
GGTAGCGGGTGCTCCCACTCGCCAGTCAACTCGATGAGGACCTGGCGTGCGAGAGGCCGGGCCTGGCTTCACCCGGCGT
CGTCGGCCAGCGCATCCCGTGTCCATCGGTTCGCCTGCTCGTAACCACTCATATCGGCGAGCAGCACATCGATTTCTCGG
GGCCCGGCAGCGGCGTTCCCGCTGATCTGGTGGCGCTCCACCAACCTGTCGGCCATGCGAAAGGCGTGATCGAAGTCAG
CGCGAGCCGAACGCCCGCCGCTAGCGGGACATCCTGGTCACCGCGGTGCAAGCGGCCGCGCACGAGGCAGCAGACATG
CCCGGACGCTGTCAGGGAGGGGTACGGTCTGGTCGTGCCCCGCGCGTGACGGGTGGTCAGCCGGGGACGTGTGCCGTT
CGACGCGCGCGACTGGGGAGCGGGGT

SCO2728 GGCAGACGCTGTTCGCGCTGCTCACGGGCCGGGACCGGGGACGGTCGGGTCGCGGTCTCGGCCATGACTCCATCCCAG
CAGCCGGGCGCCGCGGGCGCGCCCGACACTGTGTCTGCGGTTTGGGTGTCCGCGTAGACACGTGGGGCAGTGTGGGCG
GCCCGCGTCGGGACGCGAGGTGGGAACCTCCGGCGCGGAGCCGGTGCGAGCGGGGAGTTGGCGCTCGTGCCTCGCTC
CTGCCAGGGCGAGGCCGCCTTCCCGCGCCGGAGGCGTTCGGTGGGGGAACCGGCGCCCCGGCCGTGTGCCGTCGGGT
CGTGCGCCATCGCGTTCCCTCCTTGCCCGCGCCCTTGCGTCACCCCGGTTTCTAGCCCCGTGCGTGCGGCCTGTCAATGT
TTTGTCCGGACATTCGGACGACGTAGTCCGAGTGGCGGTGGAGGGGCGTACGGCGGAACACCTCCGTCCGCCGCAGTCA
ACGCGCCGCGCCTGAACGACGGCGTACGCCT

SCO2835 CTCGACCGTCACGGACGTATCAGTGGTGGGTCACTGTCACCACGGCATGCACACCTTATATAGACATGGAATGGTGACCCC
CGGTTGCATGATGCCCCCCTCGGCATCCCCTCGGCACCCGGCCGCCCAACCGGACCAGGGTTCTACGGCGTTCGTCATG
ATCGCAAGACGTCCCGGTCCCGGACACGCCGAAGGCCCCCGTGTGATACGGAGGGCCTTCGACCGTCGGTGCGCCGCCA
GGGACTCGAACCCCGGACCCGCTGATTAAGAGTCAGCTGCTCTAACCAACTGAGCTAGCGGCGCCTGCTGACCTGCACAA
CTCTACCGGACGCCGACCGGTGCTCCGGACCATGCCCGCGGCACCCGCGGGGCCGCCCCCTCGGCCGGGTACATCATTC
GGACCGTCAACATGCGCGTGCTGACGACAGTTGCCAGACTGACCGAGATGTACCGAACCGTACCGCCGCGGACCTGCCG
ACCGGGAGTGTGAGGGGAATCGC

SCO2854 TGCCGTGGGGACTGAGCTCGGTGCCGCAGTCGGCGTGCCAGAAGGTGCGCATCGGCTCGCCGCCGAGCTGCTCGCGCC
CCCAGATGCCGAGCGCGCGCAGGGCGGGCCACAGGGCGATGCCGCGTTCGGTCAGGACGTACTCGTCCCGGGTCGGCG
ACTCCTGGTACCTGCGCTTCTCCAGGATTCCCTCGGCGGTGAGCGCCTGGAGGCGGTTGGCCAGGACGGCGCGCGGGAT
GCCGAGGTGGACGAGGAAGTCGTTGTAGCGCCGGACGCCGTAGAGCGCGTCGCGGATCACCAGCAGCGTCCACCGCTC
GCCGACGACCTCCAGTGCGCGGGCGATCGAGCACTCCTGCGTCGCGTAGTCCTTGCCCAGTGCCATGCCGTCCACTGTA
GCCACTTTCCGCTCCTCGGTTCATTCACCGAACCAGCGGTGCTAGGTTGGGCCGCCCGACTCAGTTCATTGACCGAACCAA
CGGACCAAGCCAGAGCGGACGGCAGCC

SCO2923	ACGGTGCCGCGGGCGACCCCCGCGCCGGGGCCGCGGGCTCCGGCACGGCGGGCACGGCGGGCACGGCGGCGGGCTC<br>AGGGGGCTCGGCGGGCGGTGACCGTCCCGCCCAGGACGCCGCGGCCGCCTCCGGCACGGCGCGTCCGGACGCCCCGG<br>CGGGTGCCGCGCCCGGCGGGGCGGAGGCCGAGGCCGGCAACACCTTCGTCTTCCGCCGGCCCACGGCGGGACGGCAG<br>GACGCCCCCGCGCCCGCCGCCGCGCAGGACGAGGGCACCATGACCTTCCGCGCGGTCACCCCGCGCACGGGCCCGCGC<br>CCAGGCGGCCCCGGTTCTTCCGGTCCTGCTTCGTCCGGTCCCGGTCCTCTTCCCGGCTCGGGTTCCGGTTCCGGTTCCG<br>GTCCTGTGCCCGGCGCCGGTGCCGGTGCCGGTTCCGGACCCGGCTTCGGTGCGGGCAAGGCGGCGGCCGCGCGGGCC<br>GCCGCCGCACAGGCTCCGACGCCCGCCGCCCCCGCG
SCO2938	CTCAGCGCCCGGTAGGCGCGGCTCAGCACGGTCTCGGCGGGGGCGGCGGGGGCGAGGGCGGGCTCGACGGGGGCCG<br>CGACATCGGTGTCGCGGGGCTCAGGGACGGTCATGGTCGCCAGAGTAAGGGCCGCGACCTGCTTTTACCCCTGTCGGGA<br>GGCGGTACGGGACCGGGACCTTGGTCGTACGACCCGGCGGCCCGGTCGGGCGTCCGTGAACGGCGTACGGCAGTCGTG<br>CTGCGGCCCGTCCGGAACCCTTGAGCGCCTCCCCCGCCCGGTCATGGCCGCCGAGGCACCGAGTCACGGACCCGGCCG<br>TGTGCCCGAGTGGCCCAGGGGCTCGCCTGCGCGGCGCCGGTCAGCGAGCTCCGCGCGCGGGGAGTCACGCGGGTTCGA<br>ATCCCGCCCCGGCCTCCACCAGCCCGACCGGCGCGCGCATCCTCCACGGCTCCGCACCCCGGACCGGGGCGTGTTGCG<br>CTGGAGCGCACTTCAACTCGTAGGTTCCGAGGC
SCO3168	CCGGGTTCTGGTGCAGCGCCATGGCGACGCCCCGCATCAGCGCGGAGTTCTGTTCCATGGTGCAGTCGTCCTCACGCAC<br>CATCAGGGCGTGCAGGTCGCCGCGGAGCGACCCGGTGTCGACGTCGCCGATCTCGCCGGGCTTGTTGTGCCGGATGGC<br>CTTGACCACCAGCTCCGGCTTGCCGCCCCACTGGCGGTAGAGCGTCGCCTTGCTGGACCGGGTGCGGGCGGCCACGGC<br>GTCCATGGTGAGGGCGTCGTAGCCGACTTCGCGGAGCAGGTCGAGCACGGCCTCGTACAGCTCGGCCTCGCGCTCGGGA<br>GTGATCCGGCTGCGGCGTGCGGTGGCGGTCTCCGTCATCTCGCTCACTCCTCTCCGTCCGGTCGGCCGGTCCACTCGAC<br>CGGAACGACACCGTTTCGTACATGACGAATGTACCCCAGCGCCTAGCGAAACGAAACGGTTTCGTTCGTGGTCTGGGTCA<br>CGGTTGTCGGCACCGCATAAGTTGCCG
SCO3183	TCGCCGAGCAGGACCGCGAAGGGCTCGTGGCCGACGTGCGGGGCGGCGCACAGCACGGCGTGGCCGAGGCCCTTGGG<br>GTCGCCCTGGCGGACGTAGTGCATCATCGCGAGGTCGCTGGATTCCTGGACCTTGGCCAGGCGGCTCGCGTCGCCCTTC<br>TTCTGGAGGGCGGACTCCAGTTCGTAATTGCGGTCGAAGTGGTCCTCGAGGGGGCGCTTGTTGCGGCCGGTGACCATGA<br>GGACGTCGCCAAGGCCGGCGGTGACGGCCTCTTCGACCACGTACTGGATCGCCGGCTTGTCGACGACCGGCAGCATCTC<br>CTTGGGAGTGGCCTTGGTGGCCGGCAGGAACCGGGTACCGAGACCGGCTGCTGGGATGACAGCCTTGCTGATCCTGGGG<br>TGGGACTGAGTCATGCGCGCCACCATATCCGGTGCCTATTGGGAGAATCTGTGTCGCCTGAGCCTCTACGCTCATATGAGC<br>AGATCAGAGTGGTACGGGAGTGATCA

SCO3256 GCCTCGCCCAGCTCGGCCGCGCCGCCGGCGTCTACCTGGAGATCTGCGGGCAGCGCTTCGGCTCCGAACTCGGCAAGG
GCATCACCATGCTCCGCGCCCAGCTCACCGGCCGCACCGCCCACCGCGTCAACGACGAGACGTCCGCGAACATGGCCTT
CGGCGACATCGCCCCGGACGCCGTCCTCGCCGCCATCCAGATCCCCGCCGAGACACGCGGAATCGCCATCGCCGGGGAC
TCCACCGGCGGCTGGCACCGCATCCGCGCCCCGCACACCACGCTGCGCCAGGCCGTGAACCTCTGCAACAAGCACGCCG
ACCGCACCCCCGAACTGCCCGCCCTCGCGCCCTTCCGGCCCGCGGTCGCTCCGCTGCCCTCGGCCCGCGTGCCGCTGTC
CAAGACAGCCCCCGCCACCGCCTGACTCCTCCCCCTCCCACGGTCGGCGCGACCGTCATTCGCGCCAGGTCCCTACCCC
CGCCATGCCTGACAGGAAGGAGCCGTG

SCO3284 GTCCCCCTCGACCGGACGCCAGCCGGGGCGGCGCAGCACGAGCAGCGGGACCCCGGCGGTGGCGGCGGCATGTGCCG
CGTTGCGGGTGATGGTGCCGGCGAAGGGGTGGGTGGCGTCGACGAGCAGGCCGACGGCGTGCTCGCGGAGCCAGGCG
GCCAGTCCTTCGGCGCCGCCGAAACCACCGACGCGCACCTCGCCCGGTGGCGTCCTGGGCGCGGAGACCCGTCCTGCC
AGGGAGGTCGTCACGCGCGGCCCCGCCGGCTGCCCGGCCGCCAGCAGTTCGGCCAGGCGCCGGGCCTCCGTGGTGCC
GCCGAGGATCAGCACGTGCACGGCAGTCCGTCTCCCTCCTCCCGGTCCCCGCGGTTCCCCCTTCAGCGGGTTCCCGCGG
TTCCCCCTTCATCGGTTCCCGCTTCCTCCGGTCCCCGCTCTCCGTCGGCGTCGAGCCAAGCACATCCTCCGGCCGAGTTG
CCGGACGGGGCCGATGCGAGAAGGTGAGGAAG

SCO3292 TACCGGCAGGCGGTGCTCGACCAGTACAAGCTCTGCGTGGAGATGGCCGACCGGGTCAGTGCCCGGCGGAATCTGACCA
ACACGTTCTTCCTGTCGCTCAACAGCGCGGTGGTCGCCGTGGTGGCCGCGGTGTCGGGCGGCGCACTGGCCGACGCGTC
CGTCCCGCTGCTGCTGGCCGGGCTGGTCATCCTGCTGGTCCAGTGCGCCGCCTGGTACGTCATGGTGCGGTCGTACCGA
CAGCTCAACCGCGCCAAGTACGCGGTGATCGGCGCCTTCGAGGAGAGGCTGCCGGCGTTCGCGTACAGCAGGGGGGAG
TGGGGCGCGCTGGGCGAGGGCCGGGACTGGCGCCGGTATCTGCCCCTGACCTACGTCGAGCAGTGGGTCCCGGTCGTC
TTCGCCGGCTCCTACGTGACGGGCTTCTTCGCCCTCGTCACCCGGTGAGCCACGGGCCGCGATAGGATCCGGGGCCTGC
CACCAGGCCATCGACAGAGGGGGAGAGC

SCO3308 GCCGGAGCCGGAGCCCGATGCCTTGTCGTTGCCGGCGGAGCCGTTCGAGGCGGTGGATCCGTTCGAGCCGGTGGAGCC
GGTGGATTCGTTCGAGCCCGTCGATCCCTCGACACCCTTCGCGCTGCCCGCCGGCTGGACGCTGTTCCCGTCCGCCGAC
GCGGAGGCGGTGGAGTCGGGTGCCGGGGAGCCCGTGAGTTCGTCGCCCTCGCAGGCCGTGAGGGAGAGCGCGGCCAG
CGCGACCGTGGTGGCGGCGAACAGGCGGGTACGGGTGATGCGGCGTGCGGACATGGAAGTTCTCCTTCGTAAGTGGGG
AGTTGAGGCGAGCCGGTCGACCGTTCTCCGGGAGCGGCTGCTTTGATGACCACAGCTTGCGGCCGGGTTCGTCCCAGCC
GCCAGCGGTGGGCGATCATGCGGGACAATGGGACGCCAGGAACGCCCTGACCTGCGGGAACGACGTCCCTCTGGGATGC
GGTACGGGGATGCGAGGAGAGGGAGGAACG

SCO3314	CACCGTCTCGAAGCCGCGGTCGGTGACCAGCGTGGTGCACACCTTCCGCAGGAGCGTGTACAGGCCGTGTCCCGGGTCG<br>GGGTCGGCCAGGGCCTCGTCGAACGCGGCCGCGCACACGGTGAGCTGGTCGTCGAAGGCCGCGGCCACGAGGGACGAG<br>CGGGTGGGGAAGCGGCGGTAGAGGGTGGCCACGCCGACGCCCGCCCGGCGGGCGACCGCGCTCATCGGGGCGTCGAC<br>GCCCCGGGCGGAGAAGACCTCGCGCGCGGCCTCCAGGACGCGTTCGCGGTTGCGGCGGGCATCGGCCCGCAGCGCGTC<br>CGGCGCCGGAGCCTCCCCGGTGGTGTGCTCCGCGGGGTTGTGAGAGGGCTGGTCAGGCATGTTTCTCACTTCCGGTTAA<br>GTGGAGGGCATCGTCCACTTACGTGTCTAGCGTGAGATTAGCAGCGGATGCGGGCTTCGCCTCCGCACGTCCCGAACGC<br>GACGTGGGTCACGAGACATGGGTGCGAGAC
SCO3321	ATGTGCTCCGGGTCCTCGAACCCGGCCAGCCGGAACCAGGCCTGCTGCCAGGCGAAGCGGGCGAGGTCGCGGGTCTCG<br>AAGAACTTCCGCTTGTACAGGCCCCGGCCGAAGTGGAAGATCGCGGCGCCCTGCATGACGGTGTTGTCGAGGTCGAAGAA<br>CGCGGCCGCGCGGTCGTCGCCGTGGACCGGGAACTCCGGTTCGGCGACTCCGGAGCCGGGGGTGGTTTCCTGGGAGGT<br>CCCGGCGGTCTCGGCGGCTTCCGCGGACTTGCGGGCTGCCTCCGCCGAGGCCTCGCCTGCCAACACGCTCCGCGCCGT<br>GGCGGAGCGCCTACGGGGAGTGAGCCATCGAAGAGCGGCCATGCCAGTGAGCATAGCCAGCTTGTTCGGATGCTTCGGA<br>GCCGACGGGTTCGCAGGCTGTGAACTCCGGGCGGCGGTGCCGTTAAGCGGCCGTGCTGCGAGGGCTCGCCCTCGGTCC<br>CACCCGCGCTCGCGCCGGCGCGCGCGAGA
SCO3391	ACCAGCGCAGCTGAGCCAGGGGCGCCGCGCACAGGCCGGTGGCCAGCGCGGTCCGCGAGGCCCAGCGGGCGCCGGTC<br>GCGGCCACGGTGTAGATGATCACCAGGAAGGCGAAGTCGGCGGGCATCGTCGAGACGTCGAAGGCCAACTGCCCCAGGC<br>CGAGCACGACGGCGAGCAGCAGCATCCGCTCCGGCGCCCGCTGGCGCAGCGCGATCACCACGCTGAGCAGCACCACGA<br>AGGGGACGATCAGCGCCACGTTGTCTGTCCCGTCCGCCCTGGTCCCCGGGGTCGCCGCGGAGACCAGGGAGACTCCGA<br>GCACGACGAAGGCCCAGAACGAGTCGACACCGGTCGGGTGTCGGCGGAGGAAGTCGTAGAGGCGCTGCACGTAACCCAG<br>CGTAGGGAAGCGGAATAGGTGCAGGGGTCAACCGGAGGGCCGATCCGCATCGCCCGCGCATACTCCGCAAGGTGGAGG<br>GACCGCTCTCCTGTACCCCTAGCCTGGGCTT
SCO3410	CGCCTGGACCGCCGCCTGTTCACCTCGACCGCCTACCCGACCGACTACGGCTTCGTCGAGAACACCCTCGGCGAGGACG<br>GCGACCCGCTGGACGCGCTGGTCATCCTGGACGAGCCGACCTTCCCGGGCTGCCTCATCCGCTGCCGCGCGATCGGCAT<br>GTTCCGGATGACGGACGAGGCCGGCGGCGACGACAAGCTGCTGTGCGTGCCGTCGACCGACCCGCGCGTGGAGCACCT<br>GCGTGACATCCACCACGTGTCGGAGTTCGACCGCCTGGAGATCCAGCACTTCTTCGAGGTCTACAAGGACCTGGAGCCCG<br>GCAAGTCCGTCGAGGGCGCCGACTGGGTGGGCCGCACCGAGGCCGAGGCCGAGATCGAGCGGTCCTACAAGCGCTTCA<br>AGGACCAGGGCGGCCACTGATCAGGCGCCTCTCCCGTAACGGGCCGCACGCACACGCGTGCGGCCCGTTCGTGTTTGAG<br>TGACCATACTGAGGTCAACAGGAGGGT

SCO3436	GCGAGGAGGTACTGCTGGCCGTACCGGTCGGGCATCGACTGGCCGGCCGGGAGCGGGTCGCGGTGGCCGAGCTGGCC<br>GACGAGGACTTCGTCAGCACCCGCCCCGGCCACTGGCAACGGGCCCTGCTGGAGCGGCTCTTCGCCCGTGAAGGGCTGG<br>CACCGCGCGTGGTCTGCGAAGGCGACGAGGCGGCGGCCACTCCCGAACTCGTCGGGGCCGGTGTGGGCATCGGCCTGA<br>TGCCGGCCGTCGCACGCCGCGTCCTCGGCGAGGACGGCCCGGTCGGCTGGCTGCGGCTGGACGCCCCGGACTGCCACC<br>GGACGCTGACCCTGGTGTGGCGCCGGGACGCCTATCTCTCCCCCGCGGCCCTGGACTTCCGCCGGCTGGCCGGGGAGC<br>GGCTCATGCCCGACGTGTCATGAGTACATGGCGAAGCCGGTCGTGGACGGCATGAGAAACCCGCCCCTAGCGTCGAAGG<br>CAGCCACGGACGCGACCAGGGAAGGCGGACG
SCO3541	GCGGCGGACGAGACGGCGGTGCTTCCGGCGGTGCAGCCGCGGGACGCCGACGAGACGGCGGTCCTGCCGCCCGTGCG<br>CGGGGACGACCCCGCCGACCGGGTCCCGCCGGGCTACTTCCGGGAGGAGAGCCCCGCCGAGGAGGCCCAGGACCGCA<br>CGCGGGAGCTGCCCCAGATCGACCCCGACCAGGCACCGCCGTCCCGCAGGCGCCGCTCCGACTGGGCCGAGGAGACCC<br>CGCTGGACGACCTGCCGACGCTGGCGGACGAGCTGCTCGGGCCGCACGACGAGGACGAGGGCGGCCGGGACGACGAG<br>GGCCGCGGCGGCCGGGGCAGGGGCCGGGGACGACGCTGAGAGCACCGAGGCCGCCGCGTTGTCAGTGCCGCCCCGCA<br>CAATGGAGGGCGCGCCGCGGATCAGCGCGGCGGCTCGGTGTCACGGCTCGGTGTCACGGCTCGGTGTGACGGCTCTGT<br>GTGAGGGCTCGGTGTGAGGAAAGGACGGCGTGACCC
SCO3616	GCTCCCCGGCGGTCAGCAGCATGTCGAGTTCACGCCCGGCAGGGATCGGGGAAACCTGCTCGGCGAGATCGATCAGCTC<br>GTCCGTCGTGTCGCCCATCGCGGAAACGACGGCGACCACCTGGTTGCCGTTCTTCTTCGCTTCCACGATCCGCTTGGCGA<br>CGCGCTTGATGCCCTCGGCATCGGCTACGGAGGAGCCTCCGTACTTCTGCACGACAAGGCCCACGTGCGCTCCTCGCTCA<br>ATCCGTCTCTTTCCGCACGTACGTATATGTACTGCGGTCGGCTCATTTTACCGAGCGTCCGGGTTTCACCCCTGCGGTATC<br>GCATGGTGAGACTCCGGACCGCCCGCTGTTCGGGGTGGCTCATGCCCAGGGGGACGCGGGTCACTCGGGAAAGTGCGG<br>TGTGTCACACGTTCACAAGTGGCTTCCCGCCACGTGGAAGACCGGGTTGGTGGAACGCACAACCCTTGCGGCCCCTGGTG<br>AGTCCGACCCCCGGGGCGTGCG
SCO3624	TCGCTGGCCGCCGACCTGCCGGGCGGACCGGACCCGGCCGATCCGCGCATCGCCGCCGCCCTCGCCGGGGTCCGCCG<br>GCGCGGCGAGGAGGCCCAGCGCGCCGCCGCGGCCGGGGACCTGACCGTCCTGATCGGCTACGACCTGCGCTACTGGCG<br>CGAGCTGGCCACCCTCTTCGGCAACCCCTACCTCTGCGACTTCCTGCACCGGCTGCGCGTGCAGAGCTGGGTGTGCACC<br>GTGCAGCACCTGCGCCGGCTCAGCGAGCTGCGCGGCGCGTTGTGGTCCGGGCACACCGCGCTGGTCGACGCCCTGGCC<br>CGGCGCGACGTCCCGGGCGCGCGTGCGCTCGTCGACGCGTACAACAGCCACTCGCTCGCCCTGATCGAGGGGCTCGCC<br>GGCGAATGACCAACGCCGGACGTGTCGTGGCCCGGCCCCAGATGGGTATGGGTTCTGCACGCGACGCGCCCCGGCGCA<br>CACCGACTACGCTGCCCTGACCGCCGTGCTGT

SCO3636	CGCGTCGCCGCCCGCTCCTACGGGCCCGTGCCCGTGCCAGCCGCTCCTCACGCTGCCGCTGTGCGCGCTCGCGCTGCCT<br>CGCTTCGCGCACCGCGTCGTTGTTCCACCGCGCGCACCACGGGACGAGCGCCGCGGACGACACGAGAGCGAGCGCCAC<br>GATCCAGTGACCGAGGGAGGTCCCCATGGCCAGGGCGACCACGGCTCCCGCGACCGCGCCGGCCGAGTAGCCCAGAGC<br>CGGACGCCAGTCCCTGCGGTGGAAGCCGGCCTTGAACGGGACGACGAGCGGGACGGGCGCGATCATCAGCGCGGCGAA<br>GTACTTGCCGCCGCCCTCCGGGACACCGCAGCCTCCCCTCGGATGCGGCGCGCATCCCACCGTCACCGACCCGGCCAGT<br>CCCCACCCGAACCACAGTGCCAGTCCCGCCGCCAAGGCGGCCCCCACCCATTTGGCATGCATCAGCGGAGTGTATGCACC<br>GGCCGGGCCGGCGATCTATCGTCCGTCC
SCO3637	GCCGCTCCACGGTGCGCGGCGTGAACGCCTTCGACACCAGGCGCCGGATACGGGTGTGGTCCGGCGGCTCCAGGTCGA<br>GCATGCCGTGGTCGTTGAGCGTGTGGAAGGGCTCCTGCTCCGGCGGGGGCGGCGTGCGGCCGAAGTCCTCGTGCGTGA<br>AACGGTGCTGGTAGGTCCGGCCGAGGCGGCGGTCGCGCAGCAGCGCGGAGACGTCCGCGTGGTGCGGGACCAGCCACT<br>GGTCGCTCGGCTCGTAGTAGAGCACGCGCCCCCGGGCCCGCAGCTCGGCGAAGGCGGGGTAGGGGTCGGCGACGAAG<br>GCGGGGTCCCACGGGTCGAACGCGGGACCGGACACATGGGGACCATCGGTTGCAGCCGTCATGGACGGACGATAGATC<br>GCCGGCCCGGCCGGTGCATACACTCCGCTGATGCATGCCAAATGGGTGGGGGCCGCCTTGGCGGCGGGACTGGCACTG<br>TGGTTCGGGTGGGGACTGGCCGGGTCGGTGACG
SCO3697	CTCTACGGCCTCCTCTTCACGATCGTCATCCTCTTCGCCCTCCAGGGCAAGACCATCACCTCGCAGCCCTTCGACGTCGCC<br>CGCATCGCACTGCCGCTGCTGGTGTACTTCGCCGTGATGTGGGCCGGGACCTTCCTGCTGGGCAAGGCGATCGGCCTCG<br>CCTACGACCGCACGGCCACCCTCGCCTTCACCGCCGCCGGCAACAACTTCGAGCTGGCCATCGCCGTCGCCATCGCCACC<br>TTCGGCGTCACCTCCGGCCAGGCCCTGTCCGGCGTCGTGGGACCGCTCATCGAGGTGCCCGTCCTGATCGGCCTGGTCT<br>ACGTGTCCCTGGCCTGGCGCAGGAAGTTCACCCCGTCCGCGCCGGCCACTGAGGGCAGGGGCCACTGATGCACCCGCAC<br>GGTCCGGCGCATCACGGGCACGGCGGCGGCGTGGGAGGTGCGGGCGGCGCGGGAGGTGCGGGCGGCCCCGGAGGTG<br>CAGGGGGCAGGCGTGGCAGGGGTGGC
SCO3927	CATCGGTGTCGACGAGCGGATCGGTGTACGGGCCCGAGGTGTCGTAGAGCGTGACCGACTGCCCGTTGGTGAGGTGCAC<br>CTGACGGACCGGCACCCGCAGATCGGGGCGCGAGCCCTCGACGTACGCCTTGTGCCAGCCGATGGACTTCCCGGCCTCG<br>GTGTCCTCCGCGGTGTCCGTGTTCTCCGCGGTGTCCGCCTGGGCGGTGTTCTGCGTGGAGGCAGGCGTGCGTGCGTCCT<br>TGATGGTCATGAGACCTACTCCCTACGCCGGCATTACCCGGTAACAGGTTCGGCGGTCGACGCAGCGGCTTCCGTCCGTC<br>GACGCTTCGTGGGATACGTCACTCGACTTCGGTGACGTTTCCCGTGAAACATCGCTGGGACGGAGGTCAGCGCCCTCTCA<br>GCCCGGTGCTCCGAGCTCCCGCGTGTACAAAGGTGCCTCCACGCTAGCGCCCATTCGGGCGCGGTGAACAGAGGGCCCC<br>CCTCGTTCTTGCGATGATCGGGCG

SCO4082	GCCCCCGGGCCCAAGGAGGGCGACCTCTGGGCCCTGGAAGGTCTGAAGGAGCAGTTGGCGGAACTGAAGGCCGCGGCC<br>GGGGGTGTCCTGTTCACCGGCTCCGTCCGGCTGCCGACCGGGGAGCTGTACGCCTGGCAGCACGGAGCGCTGACCCAA<br>GGGCTGCTGGACGCCGACTGGGCCGAGTCCGCCGAGGTGTTCGCCGCGCTGGGGCATCCCGTGCGGCTGCGGCTGCTG<br>CGGGAGGTGCTCGGAGGGCGGCGCACCGCCGCCGAGCTGGCGGAGCTGGACGGGATGGGCACGACCGGCCAGATCTA<br>CCACCACCTGCGCCAGCTCACCGGCGCCGGCTGGCTGCACACCACCGGACGGGGCCGCCACGAGGTGCCGCCGGGGCG<br>GGTCGTACCGCTGCTGGTGGCGCTGTCGACCGCACGGAACTGAACGGGACCGCCAAGACGGTCAGGACCGTCAGGAGCG<br>TCAGGACCGTCACGAGCAAGGGGGAACGAACG
SCO4093	CGCGTCCGGCGAGGCGGACTTGACCCGAGCGCGTCACCCGTACCCGAGGGAGTGCAGCCGCGCGTCGTCGATCCCGAA<br>GTGGTGGGCGATCTCGTGAACGACCGTCACCTCGGTCTCGGCGACCACGTCTTCACGCGTCGCGCAGAACCGCAGCGTC<br>GGCCCCCGGTAGATGGTGATCCGGTCCGGCAGCACGCCCGCGTACCACTCCCCGCGCTCCGTCAGCGGCGTCCCCTCGT<br>ACAGCCCGAGCAGCTCGGGATCGTCCGCCGGCGGTTCGTCCTCGACGAACACCGCGACGTTGTCCATCAGCCGCGTCAG<br>CTCCGGCGGGATCCGGTCCAGGGCTTCGGCGACCAGTTCCTCGAACTCCTCGCGCGTCATCTCCAGCACAGGACCATTGT<br>CGGGTACGACCGTGGAGTACGACCCGCATATCCGCCCCGGGCCACGGGCATACGGGACCAATGGCCCGCGTCCCCGCC<br>GCAGTCCCGCACGCCCTGGCCGAGGTG
SCO4095	GCCCGGGGCGGATATGCGGGTCGTACTCCACGGTCGTACCCGACAATGGTCCTGTGCTGGAGATGACGCGCGAGGAGTT<br>CGAGGAACTGGTCGCCGAAGCCCTGGACCGGATCCCGCCGGAGCTGACGCGGCTGATGGACAACGTCGCGGTGTTCGTC<br>GAGGACGAACCGCCGGCGGACGATCCCGAGCTGCTCGGGCTGTACGAGGGGACGCCGCTGACGGAGCGCGGGGAGTG<br>GTACGCGGGCGTGCTGCCGGACCGGATCACCATCTACCGGGGGCCGACGCTGCGGTTCTGCGCGACGCGTGAAGACGT<br>GGTCGCCGAGACCGAGGTGACGGTCGTTCACGAGATCGCCCACCACTTCGGGATCGACGACGCGCGGCTGCACTCCCTC<br>GGGTACGGGTGACGCGCTCGGGTCAAGTCCGCCTCGCCGGACGCGTGTCCTCTTGTGGGTGACGGGAGTTGGGCACGTC<br>GACTCCTTGACCCCCGGAGGTGGCCGCCC
SCO4105	GGCCCGCACCGCGGTCAGCGCCCCGTTCAGCCCGAACGCGAACGTGCCGGTCAGGTCGAGGGCGAGCAGCGGAGCGGG<br>TTCGGTGCTCATGGGCGCCGGGTACCCCGCCGCCGGGTTCGTCAGCCGGTGGGCTCGTCATCTCCGGCGGCGGGCGGC<br>CGCTCCACGACGAACGACCCCTTGCCGCGCACGGTGACGATGAGCCCCCGTTCGCGGAGCTCCTGCGCGGCCACCGGAA<br>CCCACCGTGCAGGCGCCCCACGCCACCGAGCCCGGAGCACCCCCAGCCCCACCGCCCCCTGCCCCCGTCCGCCTCCCG<br>CAACACCACTCCCCGTACTGCCGTCACCTCCCCCTCGACGGCGCCGCCTCCCGCCTCGTCCGCCCCTGCGTCGCCGCCC<br>ACGAGCAGGAACCGGACCGACAGTGGCACCGCCGCCTCGCCCTCGTGCTCGCCGCCGACTTCGGCATCGACCTGACCAG<br>CACGCCGTCGGTGCGCGGAGGGCGGCCGC

SCO4106	CCGCCCCTGCGTCGCCGCCCACGAGCAGGAACCGGACCGACAGTGGCACCGCCGCCTCGCCCTCGTGCTCGCCGCCGA<br>CTTCGGCATCGACCTGACCAGCACGCCGTCGGTGCGCGGAGGGCGGCCGCGTGACGGCACCGCACACCGCGCTACGGC<br>TGCTGCCCTGGCCGTCGGCCGAGGGCAAGCCCTGCTTCCCCGCTCCCGGCGACGGCAGCGGATTCGTGTCCCGGCTCGC<br>CGACGAGACGGAGGTCCGGCAGCTCGCCGACGGGCGGGACGTACTCCGCTCGGCGCGCGGCGTCCTGGACGACCCGGT<br>GTCCCCGGACGCGGAGGTCCGCTGTACCGCGATCCGGCTGACGGGTGCCTCGCGGTCGCGCCGCGGTGGCGGAGTCG<br>AGGGGGCGGCGCCTGTCCACGGGCCACGGTGAACCGTAGCGGCAGCAGCGGCGCCTAGTGTGTGCCCGCCGGAACCCG<br>CCGCCCGTCTGCCTCCCGGGAGTGCCCACCC
SCO4501	AGAGCGATCTTGTTGGCGGCGGCGATCACGGCCTGGAGGCCGGTCCCGCAGGCCTGCTGGATGTCGTAGGCGGGGGTG<br>CGCGGGTCGAGGGCCGAACCGAGGACCGTCTCGCGGGCGAGGTTGAAGTCCCGGCTGTGCTTGAGGACCGCGCCGGCC<br>ACGAACTCGCCGACGGCGCCCGGCTCCTGGAGGCCGTACCGCTCGACCAGGCCGTCGAGGGCGGCGGTGAGCATCTCC<br>TGGTTGGCGGCGGTGGCGTACGGCCCGTCGGAGCGGGCGAAGGGGACGCGCGTGCCGCCGATGACCGCGACGCGGCG<br>TGGGGGCTGCGGTGCCAGAGGGCTCATCTCGACCTGCTCCTCACCCGTGACATAGGCTTACCTCCGGTAACCTTACTCCA<br>GAGTAAGCAGTAGTGGGCGGCACGAGCCCGACCGCCGGCCTCTGGACACTCCCCCACGCTCGAACACGCTCGCGCGGG<br>GGGACGCCCATCCGCCACCTGGGAGACAGAC
SCO4502	GCCGGGTCGAGCGGGGCGCCGAGCACCACCACCCGGCCGCTCGCGGCGACGGACCGTACGACGGGGTGCAGCGCGGC<br>GTGCACCTCGGCCAGCGCGTCGACGTCCCGTACGCCGGTGGCGTCGAGGACGACGGCGGCGGGGCGGCCGTCCTCGTC<br>CCGGGGCAGGCCGGTGCGGGCCAGTACGGGCGCGAGGTCGAGGTCGGTCTTTCCGGCCGTGAGGTGCAGCAGACCGCC<br>GTCGAGCGCGTCCCGCCGCAGGGCCGCGGGTTGCGGCAGTCCGAGCCGGCGGGTGAGGAAACGTCCGGGTGCGGTGC<br>CGGTGAAGCTGAGATAGCGGTCGGCCATGTCTGTCTCCCAGGTGGCGGATGGGCGTCCCCCCGCGCGAGCGTGTTCGAG<br>CGTGGGGGAGTGTCCAGAGGCCGGCGGTCGGGCTCGTGCCGCCCACTACTGCTTACTCTGGAGTAAGGTTACCGGAGGT<br>AAGCCTATGTCACGGGTGAGGAGCAGGTCGAG
SCO4503	CCTCGCCGAGCGGGAGGGGCCGGGGCGCGGCCTGATCTCGATCTGCGCGGCGGGCGGCCAGGGCGTCACCGCCATACT<br>GGAACGAACGTGATTGGGTCTGCCCGGCGGATCAGGTCGCAGGAAAGTCGCGGTGCCTCATCGACGCCGGTGAGCGGG<br>GGCTGGTGCGTGCAGCTGCAAGGCGGAGGAGGGAGTCGACGCGGAGCGTCGGCGAGTGACGACAACGCCGCAGATGTG<br>CGCACCAGCCCCCGCGTCTGCGGCATGATCCGCCGGGCAGGCCCAAACCCTCAGGTAACCCCCAACACTGCACACGTCC<br>GGCGCGGGAAGATCGTGGAACGCGCACCAAGTCCCCACCTGGGCGCCGTACGATCCCGGGACTGACCGTCGGTAACCCA<br>TGTCCGCGGAACCGGCGATGAACGCCTCGGCGCGCGAGGCACGTACGTCATGCAGCAGAGCAGCGACAGCAGAAGCAGT<br>GTCTTTGCTGCACGTCCCAGGAGCCGCCC

SCO4508	AGGCCGTCGAGGGTGTCCTTGGCGCCGCGGGTGAACTCGTCGAAGTTGTCGTCGAAGGCCTTGGAGGACGACTTCGTGA<br>CGTAGCCCGACTCGACCAGGTTCGCGATGTACTTGCGGAGGCCGTCGAGCTTCTCCTGGAGCTTTTCCTTCTCCTTCACGA<br>CATGCTTGGCGGCATCCCGCATGTCCTGATATGTGATGTCAAGGTCTTTGGGCATGAAGCCGCTTCCTCTCACAACGCCGC<br>AGGGCCAACCCCCGTGGCTCCTCGTGTGCGGATCGAACCGGCGCACCGGTCACGCCGGTCGTATGACACGACCGGTGTG<br>ATCGTCCGCTCTGAGGCAGCAGCTTACGGGGGAGGCCAGTGGTGTCACATCCCTGCGTTGCCAACGGGAACGTCCGGTC<br>ACCGCCCTGTTGCCGGACCGCGACACCCAGCGGATATCGTCGCCTGGACTGTGAGCCGTGGTGTGGAAGCGGCCGCAGG<br>GGCTCGCCTGGGGAGGACAAGC
SCO4523	ACGTCGTAGTGACGGCGGGTGCCGGTCGAAGTCCATCCGCCGTCGATGCGGCTGTTGATCTGCGTCTCGGCGTAGTGCA<br>GCCCGTCGCCGGTGGCGTATGCGGCCACGGCGGCGTCCTTGGCCGTGCGCTTGTCGACGCCCCGATAGATCGCGCGGA<br>TGTAGTTCGCCGCATCCTCCATCGCGGCCGCTTCGTTCTCGTCCTTCGGCTTGTCCCAGTCGAAGACCAGGTTCATGTCCT<br>TGGGGAGAGACACGTCAAGGCCACTGACGCTATCGACGGGGTTCCTGGTTTCGCCAGTGTGAACCGCGTCAGGAATCGTT<br>ACGGTGCTCGGGACGAGTGGGGAACGGGTGAGGGGCGAAGGGGCACGTGACTGACGTCATAGGCGGGAGCAGGGCTG<br>GATCCGTCGTACTGCGTCGGCCGGAGTTGAGCCTGCTGTGCGCCGGGGGCTCGCCGGGACTGTCCTGGCCTGCGCCCG<br>TGCGCCGGAGCAGGTGGGACCGGGCAT
SCO4650	CCGTGGTCTCATGCCGAACCCCAAGACCGGCACCGTGACCCCGGACGTTGCCAAGGCTGTCAACGACATCAAGGGCGGC<br>AAGATCGAGTTCCGCGTCGACAAGCACTCGAACCTGCACTTCATCATCGGCAAGACGTCGTTCGACGACACCAAGCTGGTG<br>GAGAACTACGGCGCGGCGCTGGAGGAGATCCTCCGTCTGAAGCCGTCCGCCGCCAAGGGCCGCTACATCAAGAAGGCCG<br>CCCTCAGCACCACGATGGGCCCGGGCATCCCGCTCGACTCCAACCGCACCCGCAACCTCCTCGTCGAGGAGGACCCGGC<br>CGCGGTCTGAGCGAGCAGCTCGCCCCACCGGTTCACGGGCCCCGCACCTTCCGAGGTGCGGGGCCCGTCCCCTTTGGCC<br>GTCCCTTTTGGCCCGTCCCCTTTTCCGGCGCCCTGGGTTAGCGTGCGGGCAACGGGTCCGCGCATGGGGCGCAGGGGCC<br>GTACGGGGCGCAAGGGGTGGGCGG
SCO4656	GACCGAGAACCGTCGTGTGGTCCAGGAGGGCGGTCACCCGGCCTCCGGTCGTCCGCAGCTGATGGGTATCACCAAGGCC<br>TCGCTGGCGACGGAATCCTGGCTGTCGGCCGCCTCCTTCCAGGAGACGACCCGAGTCCTGACGGACGCGGCGATCAACG<br>CCAAGTCCGACAGCCTCATCGGCCTCAAGGAGAACGTCATCATCGGTAAGCTCATCCCGGCCGGTACGGGTCTGTCCCGC<br>TACCGCAACATCCGGGTGGAGCCGACCGAGGAGGCCAAGGCCGCGATGTACTCGGCCGTCGGCTACGACGACATCGACT<br>ACTCGCCGTTCGGCACGGGCTCCGGCCAGGCCGTTCCGCTGGAGGACTACGACTACGGTCCGTACAACCAGTAAGCGGG<br>CAGCTTGATACGACCGAAGGGCGGTCACCCCGAGCGGGTGGCCGCCCTTCGGCGTGCCGCCCCCCGCCGTCGGTTGCTA<br>GGGTGCCGAGCACCTGGGGGGGGAA

SCO4658 GCGCCGGGTAGAGCGGGAAGAACGCGACCGAGTTCTGCTGGACCGTGAACAGGCCGTCGGAATCCAGCCGGACCAGGG
CGGGGCTGTAGCCGTGCTCGGCGACCTGGAGGTACCACCAGCCGTCCCAGGTGGCCAGCACGTCCCACCAGTGGGCGC
CGCCGCCGAAGCGCGGGTTCTTCCCCCGGAAGTCCCCGGCGTGGGTGAGGAGGGAGGCGAAGACCCCGAGGCCGACGA
GTTTCGTGACGCCGTAGAGCGCCAGCGGGGCGAGGTAGGGCCGGGCCCCGGGCGGGAGGGCGGGGAGCCGGCTCCGC
CAGGAGCGTGCCGCCCCCTCGCCGCCGGCCGGCCGTTCGCCGGTGCCGTCCGGCCCGTCCGTCACCGTGTCGTCGGCA
GCCGTGTCCATGGCTGTCATCCCCCTCGGGCCACCCTCAAGCGTCAATTGCTGCCATAAGATCGCACAAGAGTCGCACATC
TGTACTCAAGGTCATGAGGAGGGAGGCGCG

SCO4733 GTAGACCGTCTCGCCCATGGTCAGCTGGGTCCCGGGGAAGAGCAGGTCCGGGCTGAGCGGGGCCTTGTCCTTCTTGACG
CTGGAGACGAAGTCCTTCGGGTCCAGCGGCGGCGGGGCGCTGGTCGGGGCGAAGGACGGCTCGGTGGCCGTCGCGCT
GGGTATCTCCGCGGTCGTGGGGAGCTGGGAGGAACCGCCGTCGGACGCCTGGTCGGAGCCGTTCGCCGAGACCACGGC
CACCGCGACGGCACCGGCTATCGCCACGGTGGCCAGCGCGCCGCCGCCGATGAAGAACAGCCGGCGCCGCTTGGCACG
GGCCTCGGAGGCCTCGGCGAGGGCAGCCCAGTCCGGCGTGTCGTCACTGTTCCCGCTGTTCCAGGGCTGCTGCGACGG
CGGCTGCTGCGATTGCGGTGTCCAGGGATCCCACTGCGACTGAGGTCCCCCCTGCCCAAAGCTCATGGGCGGCATCTTAG
ACGGGCCTGTGGGCGTGCTGGTGCCGGGAC

SCO4742 AGAGCACGTAGAGCCACTCGTGGCCCTCGTGCGACCCCTGTTCCGGGCGCGGTGTCCACTCGGTGCGCGGGGTGGCCG
GGGCGGGGAGGATCTGCTTGTAGGCGTGCAGGCCGTCGTTGGGGTTCCGGGTGAGCGGGACCCAGGTCTGTCCGTGCC
GGGTGAAGGGGCGGGGATGGACGCGGGGGTCGCCGGTGCGCGAGCCGACGAGTTCGTCCAGGGGCAGCCGGTGCGCG
CGGGCCAGCGGCAGCAGGTGCCGCAGCCCCGGCTCGCGCTGTCCCGACTCCAGCCGGGACAGGGTGCTCACGGAGATG
CCGGTGGCCGCGGCGAGCTGGGCCAGCGTGAGCCCGCGGGCCTGCCGGATGGCGCGCAGGCGGGCCCCGAGCGAGGT
CATCACCTGTGCCGTCTCGTCGCCGCCGCTCATGGGACCACTGTCCCCCATCGCGGATTCCGGGGAGCGGCGGGCCGTG
CGTGTTCCCGGCGTACGGGGAAGACTCGACGGC

SCO4743 CCTTCCGCAGCCCGCCGCTGCCGCCGATCCCGACGATGCCGTCGAGGACCAGATCGGCCCGCGCGATCAACTCCTCGGC
GGAACCGGCGGGCCCGCCCTCCGCGACCCGTCCGCCCGCGCGCCGCAGCGCCGTCAGACCGCCGCCGTGCGCCCGGT
CCGGCGCGAGCAGCACCGCCGTCACGCCCGCGCCCCGCCGGGCCAGCCGCGCGCCCGCGTACAGGGCGTCGCCGCCG
TTGTCGCCGCTGCCGACCAGCAGCACCACCCGGCTGCCGTAGACCCGCCCGGCCACCCGGGCCAGCACCTGGGCGCAG
GCGGCGGCCAGCCCGGCCGCGGCCCGTTGCATCAGCGCCCCCTCCGGCAGCCGTGCCATCAGTTCCCGCTCGGCGTTC
CTGACCGTTTCCACGCTGTACGCAGTCCGCATGCCGTCGAGTCTTCCCCGTACGCCGGGAACACGCACGGCCCGCCGCTC
CCCGGAATCCGCGATGGGGGACAGTGGTCCC

SCO4746	CGGCCACGGCGTCAGCTACGGGCACCACTACACCACCCCCGGCGAGACCACCCTCGGCCTCGTCCCGCTCGGCTACGCG<br>GACGGCATCCCCCGGCACGCCTCCTCCAGCGGCCCCGTGCTGGTCGACGGCAAGTGGCGCACGGTCGCGGGGCGGATC<br>GCGATGGACCAGTTCGTCGTCGACCTGGGCGGGGACCGGCCCGAGCCGGGCGCCGAGGCGGTGCTGTTCGGACCGGGC<br>GACCGCGGCGAGCCCACCGCCGAGGACTGGGCCCAGGCGGCGGGCACCATCGCGTACGAGATCGTCACCCGGATCGGA<br>AGCCGGGTCCCGCGCGTCTACGTCAACGAATGACGCTCAGCGAATGACGGCCGGCGAACGGACGGATGACGGTCGGCGC<br>ATGGCGGCCGACGAGTGACACCCCCGTCGGCGGCGTGACGCGCGGGGGCCGCGCGTTCAGGTGAATCAGTGGCCGGCG<br>GGCGTCCGCACGGCCCGGCGAAGAGGAGCG
SCO4757	TGTCGATCCGCGGCCGGCCCGCTCGACGCAGGGGTGAGAGGCCGGGAGAAGCCCGGCAGTACGACCACCCGAGGAGCC<br>ACCATGCCCCGCTACCTGTCCCTCATCCGCATCGAGGAGTCCGACGCCGTGCCCGGCGGCCCCAGCCCCGAGCTGATCC<br>AGCGGATGGAGAAGCTGATGGAGGAGATGACCAAGGCCGGCGTCCTGCTGGACACGGCCGGCCTGACCCCGACCAGCCA<br>GGGCACCCGCGTCCACTACGAGGGCGGACAGATCTCCGTCACCGACGGGCCCTTCACCGAGACCAAGGAGGTCATCGGC<br>GGCTACTCCCTCCTCCAGGCCAAGGACCGGGCCGAGGCGGTCGAGTGGACCAAGCGGTTCCTGAAGGTGCACGAGGAGT<br>ACTGGACGGTGACCTGCGAGGTGCGCGAGCTGATGGAGGGCTGAGCCGGCGCGGGGCACCGGTTCCCGCTTTGTCCCG<br>GCGCCCCGAGGGTGTTGCATGGTGGGCT
SCO4758	CGGGCCTGCATCTTCTTGAAGTAGCCGTAGTCGTCCTTGATCTCCTCGTCGGTGAGGAAGACGGTGTACGGGATCTTCAGT<br>TCGCTCATCATCCTCAGGAACGCCGGGTCCTTCTCGGCGCCGTCGTCGATGGTGAGGAAGACGACCTTCTCCTTGGTGGG<br>GACCGTGGTGAAGACCGGCGGGAGGCCGTCCTCCTCCTGGCCGTCCACCTCGAAGCCCTCGCGGGCCTCGATCTCCGGC<br>TTCTTCGCGGGGGGCGCGGGGGCCGTCAGCGGGACCTTGTCGAGTTTCCAGCGCTTGGCCGCGGCGGCCAGGGCCGCC<br>CGGGCCTCGCGAGCCTTCGCCGCGCCGTCGACCGCGCGGGCCGGGGGTGCCTGCTGACCGGGGGCGCCCCGCACGCT<br>TTGGTCCTCGCCCTGCGCACAGCCGGCGGCGAGGGCGGCGACGGCGAGGGCGGTGGTGGCACGAGAGGCCCGGGACA<br>CGCGACGCGATGCCCGACTTTTGTCATT
SCO4787	GGTGGAACGGCTCCGGCGGTTCCACCTCTCGCACGTATCCGGCCTTGGTCCGCGGGTCCTCGACCTCGACCGCCCGGCC<br>GGCGATCCGCACGTCCCCGCCGCCCATGCCGGTGCCGTCCCCCGGGTTCGCCTGGAGGGCGAAGCGCGGATCGCGGCG<br>CAGGTCGAGCGCCTTCAGCGAGTCCGGCATCATGCCGAGCCACAGCTCGCCGGTGAGGAACCGCACCTCCAGTCCGGCG<br>GTGCGGGGCGAACCGTCCTTGCGGAGGGTGGCGAGCACGTGGTGCGGGTACGCGCCGAAGCGTGCCTCGACGGTGCCG<br>GCCAGGCCCGGTTCGGCGGCGGCGAAACCGGCCCAGTTCACAGGGGAGCCTGAAGTCATACGCCGAGTGTGAACGCCAA<br>AACCGACATCCTCTGTCGCGTATTGGCCCCCGACCCTGTCCTCCCGGCCGCACCGCCGGTAACTTCCGGCACGTAGGGCA<br>CATCCGGCGCCGAAGGACAGAGAACAC

SCO5141	TGCTGCTCCTCCCGGTCCGGCTTGTCCATGACGACGTGGCGGTGGCCGCCGCGGCCCGGGCGCCCGCCAGCGCGGAC<br>TCCAGGTCGGCGATCCGGGCCTCGCGCTCGGCCAGCTCGGCGCCGAGGCGGCCGAGGGCGTCGTCGACGTCGGCCATG<br>CGGTAGCCGCGGACCACGAGCGGGAAGCGCAGCCGCTCCACGTCGCCGCGGCCCACCGGACGGTCCGGGGGCAGCGC<br>GTCCCTGACCCGCTCGGGTGCGGCGTCGGGCAGCGGCCCGCTCTCGCCGCCGCCCACCACGGCGAGCGTCACCGCGGC<br>CACCACGACGGCCAGCGCGATGACGAGGAACAGGAACATGACCATCGCTGGGCCCCCATGGTCGGGTCGGCGTCGGTTG<br>TGCCGGAGTCGGTTGTGTCGGGTCCGATCGTGCCATGCGAGTCTGACAGTCGGGGCCGCGGTGGTCGAAGGTCCGCCCG<br>CCCGGGGTCGAGAGCGGCACGAGAGAACGGT
SCO5321	CCAGCATCAGGACGACCCGCACGCGGGAACCGTCGAACGCGGATATCGACACGCCACCCGCGTCCGTCGCCACGGCGG<br>GCGACGTCGCCGACGTCGCTGTGGTCCGCGTCGCGTCCGTGCTCGGGGCATCCGTCGCTACGACGGGAGAGACCGTCAT<br>CCGGCCCACCTCTTCTCCGGTCATCCTCGTGGACTGGGGCGATCGTCGGCGGCGCCCGCCAGGCCGGCGGGGTGCGAC<br>GGTCGCGTAAGCACGATCGTGGGCCGTGCCCGCGGGGACCGCGAACCAGGAGGGTTAAGCGGGTGAAGCCTGCGCCGT<br>GGCGGGCCCCGACGGGCCGTACGGGGCATGCATGAAGGCCCCGGCGCACCTCGGTCGGACCCCTCTCGGACCCCTGAC<br>GCACTCCTGACGCATCCCTGACGCGCATCTGAACGCGCGTCCGACGCACTCCCGACGCACTTCCGACGCAACCCCTGACG<br>GACACCTGTCGTACAACGGAGGACGAATC
SCO5401	CGCGAGGTCGAGACCATCGCCGTCACCACGCTGCGCGAGCGCATCGGTGACCTGCACGGGGACCGGCGGCTGGGCGCG<br>CTGGCGGAGCGGATCGTCGCCGGGGAACTGGATCCGTACCGGGCGGCGGACGAGCTGGTGGCGGGGCTGACGCGGGG<br>CTGACGCGGGGGCTGAGCGGTGCGGGGCGGTCTTCCGGCGCGGGGTTTCTTCGCCCCCGCCGCCCCTTCCCTTCCCGA<br>CCCTGGGGGCGCCACCCCCAGACCCCCGCTTTCGGCCTGAACGGCCTCGTCCTCAAACGCCGGACGGGCTGGGGGCTCA<br>ACGGCCTCGTCCTCAAGTGCCGGACGGGCTGGGGGGGCTCAACGGTCTCGTCCTCGAGTGCCGGACGGGCTAAAGTGAC<br>CCGCGTGTTCCTCCTCTTGGCCTAGGACCCGCCCCGCACGCGACCGCCGCATCCGGCCGTCGCGCGCATCGGTGTCCCC<br>TAGTTGCCTTCCTGCTGAGGAACCCTTCGC
SCO5462	GCGGTGTCGATGCCGGCGAAGACCTCGGCCATGAAGTCCTCGATGATCCGGCGGCGTTCGGTGGTGGACAGTCGTGCCA<br>GCTTGTTCATCAGCGTCGTCTCCTCGGCGGTCGAACCGCGTCGCGCCACGGTCGACAGCACCGCCCGGGTCACCCTCAG<br>CGCGGCGATCTGCGCGTCCAGCGCGCTCACGTGCGCCGCCGCGACCTGGGCGACGGTGGTCTCGCCGGCCAGCACACG<br>GCGTACGGCGTCCAGGCCGAGGCCCAGTTCGCGCAGGGTGCGGATCAGCTCCAGGCGGGCGACGGACGCGGCGTCGTA<br>GAGCCGGTAGCCGCCCGCGGAGCGGGCGTGCGGGGGCAGGGCGCCCTCGTCGGACCAGTAGCGGATGGTGCGCACCG<br>TGAGGCCGGTGGTGCGGGCCAGTTCGCCGATGGTCATGAGGCCGGTGCCGTCGTCGGTCATGTCTGGGAGTGTGGGCCT<br>TCCAGTGGGTGGAGACTCAAGGAGCGCGGT

SCO5551	GCGGCCACCGCCACCACCAGGGAGCGGTGCACGGGGGAGAGCGGTGCCAACTCCCGGGAGGTCTCCCAGCCCAGCAGT<br>TCGAGGAGCCGGTCCACCTCGACGCGCGCGGCGGCCACCGCCGGGTCCGCCTCGTCCGGCTCGGCGTCCTGCGGCAGC<br>GCCCACAGGGCCGCCCCCAGCCGGACGGTGCGGCCCAGTGAGTCGTCGTCGACGTGCCCCAGCACCTCCCGGGCCGTC<br>GCCACCGGCACCTTCCCGACCTGGATCAGCGCCCGCACCAGCCGCAGCCGGCGCAGATGGTCCTCGTCGTACTCGGCGG<br>TGGTCGCGTTGACCTGCCGGCCCGGCGGCAACAGCCCTTCCCGCAGGTAGTACTTGATCGTCGCGGTGGACACCCCGCT<br>GCGCTTGCTCAGGTCGGCCAGTCGCATCCCTTGCGCCCTTCCTCGGATAGCGCCACTATCTAAGCAGCATCACTTGGACA<br>GTGCAGCTCGCCAACAGGGGGAGGCGTC
SCO5565	GCTCCCTACAGCGGACCAGGCAGTGGCCGCGCGCTCAGCTGAGACGCGTCAGCGCGAGACCTTGCCGGCCTTGATGCAC<br>GAGGTGCAAGCGTTCACGCGCTTCGGCGTCCCGCTCACCACGGTACGCACACGCTGGATGTTCGGGTTCCAGCGACGGG<br>ACGTACGGCGGTGCGAGTGCGAGATGTTGTTGCCGAAGCCCGGCCCCTTGCCACAGACGTCGCAGTTGGCAGCCACGGG<br>TCACTCCAAAGACTTCAGATGCACTTACGGTTGATCCCGGCATGCCGGGATCAAGGTCTCGGAATCGCGAACGGTCCGGG<br>ATCTGAGTGGCGGTGCCAGGGGGATGGCCCGATCGGGATCGGGCAACCGGAGCAGCATACAACGGCTGCTCCCGTACAA<br>CGAAACTACCACGGCAGCTCAGGGCCCCGTCCCGGCCCTCTTCCGGTGGGCGGCCCCCCCAAGGTCTACGCTGCCTGCC<br>ACGTCCGCAGCTCAGGGAGGCGCAG
SCO5577	CGTCGGCGGCTCCGACCCTCGACTCCTCGCCGAGATGCGACGGCTCCAGCAGCGCGTCCAGGACCTGGAATCCGAACTC<br>GGACGAATCCAGGCGGAGAACGACGCGCTGGCGGCTGCCGCTTCTCACGACAGGATCATGGAGAGCATGGAGAGCGTTG<br>ACGCACACCAGGCGGAGCCTGCGCTCACCTGATCACTGCATCGCTCCACCACGGCACGGCAGTGGCCGGGCCCGCCCGT<br>ACCACCGCTCAGGTGCCAGAGTCTGCAAGGGACGCTTCGGCGTCCCTTCTTTCTTTCCCGCCCCCCGTCTCGCCGGTCCA<br>GCCGTTTCGTACCGTCTCGGTGCCCGCCCCCGTCCGGTCCGTGCCCTCGGCCCGCCCCGTCCCTGCCCCGCGCAACCTT<br>TCACCCTCTTCAACGTCTGATGTGCCCTGCACGTTCACCGGCGAAACCGCGTCGGTTCACCGGTTCGTGGAGTGAGACAG<br>ACCCGAAAGGTAGAGTCCGACGGC
SCO5613	CGAAGGGGCCACCGTCGCCAGCGACGTGGTCTGGGGCCGGTACTCGAAATGGATTGAGGCGGGCGGCGTGGACTTCGT<br>GAAGAAGGCCGACGACCGGGCTGGTAAGAGCCTGCTGGTCCGGATGCTCAAGACCTGGAGCATCGAGAACGGCACGCCT<br>ATCGAGTCGTTCAAGTCGAGCAATACCCGCAAGCTGCGAGGGGTCGCCTTGAACGACTGACGCGAGCAGCCACGGGCGC<br>GGGTGGAGCGGTCGGCCACCCGCGCCCCGGATGCATGTTCGGGTACTTGCGTCTCGCGCTAGGTACATAGGTACGCACT<br>AGGTACGCTCCGGAGCCGCTTAGTGCCAAAAGGGAAGCTAGGGCGATTCGGTCTCGGGTGTACCTAGCCCGTACCTATGT<br>ACCTTGTCTGGCGCGCAAGTACCCGCAGCGCGGGGTGGCTTGGCGGGTCTGAGGGGCGGGGCGACATCCACTCGGCCG<br>GACCACCGACAGGGGAGAGGCAGAGCA

SCO5675 GTGACCCGAGGGGTAGTAGCCGACCGCGGGCGGCACCGCCGGGGTGCCCGGGCGGTCGGTCCACTCCTTCAACGGGGC
GACCAGCGCCGGCACCAGCACCATCGCCAGGGCCCCGGCCAGCGGCGGCAGCCACCACAGGCCCACACCCGCGACCCG
CCCGTGCCACGCCGCGTACGCCAGCGCCACGACCAGGACCGGCACCGCCACCTGCACGTTGCCGAGGTCGGACAGCAG
CTCGGAGAACCGGTCGGGGTGGACGAGCGCCCCGCTCAGCCGCTCGTCCACCCCGACCAGGGGGCCGTCGGCGACGAC
CTGCCAGGTGATCAGCGCGAAGAGCAGCACGGGAAGCCCGACGAGGGCGGACAGGGCGCGAGGCGGGGCGGAGACCG
GCATGACGCCCAGCTTCGCAGGCCAGGCCTCGCGTAGTCTCACCGCCCGTGACGACCATACGAACGGCACGGGACAGCC
AGTGGGGAGAGACCTTCGCCCGGCTGAAGGCG

SCO5748 TCATGGCCGACCGGCTCGTGCCGCTGCTGCGGCAGCCCACCCGTCGGCCCGGGATCCGGGGGCTGCTCCCCCGGGATC
AGTCCGGTGGTCATTCCGGCCGCCCCTCCGATGCCCGCTCGTCTCCGTGCCACCGCCCAGACCGGACGGACCGGTTTGG
CTGGACAGCCGTATGCAAGGTTACTTACCTTCGCCGTCCATCCGGATGCCGGTCTGCAGTGTTTCCGCCCAGAGGGTGTG
CGGACGATGTGCGAAGCTGCCGAACTGTTATGGCCTGGTTCGGCCGGGGTGAAACACTGGGCAAGCTGCTGATGAAGGT
ACGGGCAGGTGAATGCGCGCCGAGTCGGGTGGGCAGCAGCCCTGGAAGCGGTCCGGCGCGCCGGCCCGGATCACCCG
GCAGTACGGAAGTACCGGCAGTACGGGAAGTAAGCGGCAGGAACCGGTCCGCCGAGTGTGGGCGGCCGGGCACAGCGG
TATCGGTCGACCCCTGCGGGAGGGACACA

SCO5765 AGGACGTTGATGAGCAGCCCGGTGAGCTGGTGGAAGGCCCAGCTGCCCAGCTGGAAGGCGGCGATGAGCGCGAGGGCC
AGCACCATGGCGCGCGGCAGCCAGCGCGGCATGCGGGCGCCCGGCCCCGCCGCGGAACCGTGGCCGGGGGGCCGGC
GGGCGGCGCGGTGCCCGCCGGGGGTGCGTGCCGGGCGGCCTGCCCGGTCTCGTCAGTGGATGCCACGGAGCAAGTCTC
GCCCACGCCACCGGCATGAGAGCCCGGTACGGCGATCTTCGTGAACCGTCACCGCTGTTCCCGCGGCACGTTCATGACG
GTGCACACCACGCGCCACACGTCCTTCGCGTCCCAGCCGGCGTCCAGCGCCTCGTGCACCGTACGCCCGCCCAGCTCCG
TCATCACGTGATCCCGCGCGAAGGTGTCGGCGTACCCCGGACCGAAGTGATCCGTCATCCGCTCCCAGAAGACCGTCAAC
CGCATGCCCCCAGTATCCCGCCCCTGAGA

SCO5766 AAGGGCGCGGCCGGTGCCCCGTCCCGGTCCGCACGGGTCACCGGCAGGGCACCGTCCGGGGTGGACACGGCGGTCAG
ATAGCGGCAGACCCGTTCGGCCCGCTGTCCGCCGCAGCGGCCGACGGCGTCCAGGACGCGCAGCGCGTGCGCGGTGTG
CAGCGGCTGACTGGCCGGGCCGCGCAGATCGGGCTCCAGGGCGTGGCCGTACCCGCCGTCGTCGTTGCGGTAGGCCTC
CAGCGCGGTCTCCACCGGGCCGGGGTCCCCGCCGCGGAAGTCGTGCGCGAACAGCCGCTGCTCCAGTACGCGCGCGGT
GAGCCAGACGAAGTGCTCCGCGCGCGCGAGCGGTGAGCGCGGGCCCTGGGCGCGGGGGACGCGGGCGGCGGGATC
GCGGGGAGTGGGGACGCTCCGGTTTCGGCCATGGGACAGACCGTAGGCCGAAAGGCGGTTCCGGCAGGTGGTCCCGGC
TCGGGGGCCACTCTCAGGGGCGGGATACTGGGGGC

SCO5767 GGGGACGCGGGCGGCGGGATCGCGGGGAGTGGGGACGCTCCGGTTTCGGCCATGGGACAGACCGTAGGCCGAAAGGC
GGTTCCGGCAGGTGGTCCCGGCTCGGGGGCCACTCTCAGGGGCGGGATACTGGGGGCATGCGGTTGACGGTCTTCTGG
GAGCGGATGACGGATCACTTCGGTCCGGGGTACGCCGACACCTTCGCGCGGGATCACGTGATGACGGAGCTGGGCGGG
CGTACGGTGCACGAGGCGCTGGACGCCGGCTGGGACGCGAAGGACGTGTGGCGCGTGGTGTGCACCGTCATGAACGTG
CCGCGGGAACAGCGGTGACGGTTCACGAAGATCGCCGTACCGGGCTCTCATGCCGGTGGCGTGGGCGAGACTTGCTCCG
TGGCATCCACTGACGAGACCGGGCAGGCCGCCCGGCACGCACCCCCGGCGGGCACCGCGCCGCCCGCCGGCCCCCCG
GCCCACGGTTCCGCGGCGGGGCCGGGCGCCCGC

SCO5768 CCCGAGGACTCGGGGCCGTAGACCTCCACGACACGGCCACGCGGGATGCCTCCGACGCCGAGGGCCACGTCGAGCGCG
GTAGACCCGGTCGGGATGACCTCGATGGGCTCGTTGGTCCGGTCACCCATGCGCATGACCGCGCCCTTGCCGAATTGCC
GTTCAATCTGTGCGAGCGCGGCGTCCAGGGCCTTCTCGCGGTCGGTTCCTGCCATGGGTTCCACCCGGTTTGCTTGATTC
GATCGCTTCACGTCAAAGACGCTAATGCCTGCCACTGACAATGGGCCCCGATGCGGGTCCGGCCTGTGGATAACTCGGGT
GCATCTCACCCCGAACCCACTCGAATCACCCGTCACGAGCCCCGCCGGAGCCTCCATAAGAATGGATGTTCGATTTTCGTG
TCAAGCGCACCACGCGGCACTTCCGAGCGTACGTCCCCCGGGCGGCGAGCCAGGTGGTTGGATGCGGATGCGACACCG
GGAGAGGTCGGAGAGGGGACGCAC

SCO5781 CCACCCGCTTGCCACGCAGGTCCCGCACCGACTGGATGTCCGAGTCGCTCGGCACGACGAGCTGCACGTAGTCGTCGTA
GAGCCGGGTGACCCCGCGCAGCCCTTCGGCGCCCGGGCTGCCGGCCAGCTTGTACGTCTCGACCGCGTCGGCCGCGGC
GATCGTGAAGTCGGCCTCCCCGGACGCCACCCGCTCGACGTTCTCCTGCGAGCCCGCGCTGGTCATCAGCCGCACGTCC
AGGCCAGGCATGTCCTTGCGCAGCTCGGTGCGCAGCAGCTCGCCGTACTTCTGGTAGACGCCCGCGCGGGTTCCGGTGC
TGAACGTGATCGTCCCGCTCGGCGGCTTCTCGCCCAGCGGCAGCAGCCACCACAGCAGCAGCCCGAGCACGACGGCACC
GGCGACCGTGCCCTGGACGGCCCGGCGCCTGCCGATACGGGAGAGCAGCGTGGACATGGGGGCGATCCTGCCAGCCCG
CACCCGCACTGACCAGGGCCCACTGTCA

SCO5786 CCCAGGGGGCCCGCTTCACGATGGTGTCGCGGTCCTTCTGTGCCAGGCAGCCTCCGACGGCGATCTGCATGCCGGGGCG
GCTCGCCTTCTTCGGGGCGAGGTGGCCGAGGTTGCCGTACAGCTTGTTGTCGGCGTTCTCCCGTACGGCGCAGGTGTTG
AAGACGACGACGTCGGCGTCGCCGTCGGCACCCTCGGGGGCGCGGACGTAACCCGCGTCCTCCAGCAGACCGGACAATC
GCTCGGAGTCGTGGACGTTCATCTGGCACCCGTAGGTGCGTACTTCGTAGGTGCGCGTGCCGCCCGCTGACTGGCTCCG
GTCGATGCTGCTCATGGCACTAAGGGTAGGCGGTCACGATCGGGGGGAACGCCCGCACGCATCTTGACAGTACACCGCC
AATCTCGATGTGATGCATGGGAGCGCTCCCATGCACCGTGCATGCCCCGTCCCGGCCCCATGCATGTGCTGACACACATC
CCTTCTCCCGCACTGGAGGCACCCT

SCO5788	CGCCGAGATGGAGAACCAGATCCCCAAGGCGGTCGTCCAGGAGCGCTACGAGCGCCTCGTCGCCCTCCAGGAGGAGATC<br>TCCTGGGACGAGAACAAGAAGCAGGTCGGCCGCACCCTGGAGCTGATGGTCGCCGAGGGCGAGGGCCGCAAGGACGGC<br>GCCACCCACCGTCTCTCCGGCCGCGCCCCCGACAACCGCCTGGTGCACTTCACCAAGCCCGACCAGGAGGTCCGCCCCG<br>GCGACGTGGTGACCGTGGAGATCACCTACGCCGCCCCGCACCACCTCCTCGCCGAGGGCGCCGTGCTCGACGTGCGCC<br>GCACGCGCGCGGGCGACGCCTGGGAGAAGCGCAACACCGCCGAGCAGGCCAAGCCCGCGGGCGTGCTGCTGGGACTG<br>CCGAAGATCGGCGTTCCCGAGCCCCAGCCGGTCGTGGCGAGCGGCTGCGGCTGCGACTGACCGCGGGGGTACCGCCGG<br>GGGGCGGCGACACGGGGATACGCTGCCGATC
SCO5949	CACGCCGAGGGCTTCGCCGCCTACGAGCACGCCACCCGTGACTTCGTGACCCTGAACCAGGACCTGGTCGGCGAGGGCG<br>GCGCCACCCTCTTCCCGACCACCGTCCAGGCCCTGGAACAGCGCAACGCCCGACTGCGCGCCCTCAGCGCCATGCCCGC<br>CCCCGAACCCCGGCCGGCCCACTCGGCCCTGACGCTGCCGTCCTTCGGCGTCCGCTGACTCCGCTCGGTTCCACCAGCG<br>CCGTTGCCCGAGCGGAAGGGCCCCGGTCACCCCACCAGGGTGGCCGGGGCCGCGCCGACGGACCCGGCCCGCGGTGA<br>TGCCGTCGAAAACCGCTGGAACAGCGCCGCCGTGTGTCACTACCGTGCCGCCGGAACAAGTCGCCCAGGCAAGGACGTG<br>CCGTTGTACACCCTGTGAGACCTCCCGAGTGCTGATCTGTCGCGCGTCGCACATGTGCCACGCGCTCCTTTCCGCTGCGG<br>TCTTCTCACTGGAACCGGTGTACCTCT
SCO6075	GCGCCAGCAGGTGCATGGCCCAGGCGTTGTCGCTGATGCCGAAGCGTCCGGATCCGTCGAGGAGGGCGGCGGTGGCCG<br>CGTAGGGGTCCTTGCCGTCGGTCCAGTCGCGCAGGGTCAGGGCGGGGGCGCCGGCCGCCTTCGCCGCGTCGGGGGCC<br>TCGAGCGTCGGGACGACGAGCACGGGGTCGCGGTCCGGGGCGAGGACGAGCAGCGTGAGCCGTTCGGTGTCCGCCGTG<br>GGCGCGTAGCCGGTGAGCCACACCAGGTCCGGGCCCGGGGCCACCAGCAGCCCGGCGAGGCCGGCGTCGGCGGCAGC<br>CCGGGCGGCGCGCTCCATACGGGCCCGGTAGTCGTCGGCGGTGAAGGAAGCGGCGGGGCTGCTGCCGGTCATCCGGGC<br>CTCCCGGGGCGGAAGAGTGGGCGGAGGAGTGAAGGGACCACGGGCAGCATCCTGCCCGCAGGACGGGGGCGACGCGA<br>GCCGATTGGGATTGATCGACCCTGACGACTAATGG
SCO6137	CGGCCATGAACCCGACCAGCCCGATCGCACCCTGGACCGAGCCGAGTCCGTCGACCTCGTAGTACCCGGAGGCGAAGAG<br>GTTCGTGATGACGGCCCCGAACAGTCCGAAGCCGATCAGCGTCCCCCAGCCCGCCTTGGCCGCGGCCCCGTCCAGGGCC<br>ACGTCCAGGGCCTCCCGGCGCTCGGTCCGCCGCACCACCCAGGCGTACACGAACACCACCAGCGCCACCGACGTGAGAC<br>CGACCACCAGCGTGAGCCAGTCGTTGTCCTTCACCGCCTGGACGGCGATGCCGCCGATGGCGTAGGCCACCAGCACGGC<br>CAGGAACTGCCACACGAACCTCACGGGAACTCCTTCGTCGGGCCCGTGGCTCGAGTGCCGCGGGCTGCACCGGACGCTA<br>CGGATCCGGCGGCCGGAGATCGTCACCTCACGGTGGACACCTGTCTGTAGCTCGCTCGGGGGACAAGGGCCCGTGGCCC<br>CGCCGGACAGGTCGTAGCCTCGTGCC

SCO6149	CTTCATGTCGCACGGCATCACGCCCCGCTTCACCACCTGGTGCCCCGAGCCCACCACCCCGCTCGGCAAGACCAACCCCG<br>ACGGCGCTCCGCTGGAGTACCACATCCGCCTGCTCCAGGCCTACCGCCAGACCATGGAGGACTACGGTCTCTCCTCGCCC<br>CCCGGCTACGGCCCGCCCGGCGCAGGCAACGCGGTCTTCTCGGTCAGCTCCTTCATGGACAGCCTGCCGGAGGACGCGC<br>CCGTGGAGGTGTGAGCGGACGGTTCCGGGGTTTATTCGGTGGCGCGGCCTTCGGGCGCGCGACTAGGGTGACCGATGT<br>CCGAGCGGCGGCCCAAGCGGCCGCCGACAGGGGTCTTTTCCGAGGAGGTGTCGACCAATGGCTGTCACTGTGACGGGC<br>GCTGCCCGCATCCAGGAGTCCATCGAGTCCACCTCCGCGGCCACCGGCTGATCTCAGCCGCTCCCTGAGTTCTCGCGCC<br>GGGGCCGCACCCCGAAGGGTCACCCC
SCO6242	TCGCTCCGGCAAGCGCGGCTACGCCTTCCTCCGGATCGCGACCGCCGAAGCCCCGTCATCTGGCTCCGTGCATGATCGG<br>CCGGGCACGTCATCGTTACCGAAACGGTCATGAGGTCTGCGGCTGCCGTCCGCGGAGCTGTTCGGTCAGCCGCTCGACC<br>GGCCCGGGCGAGGACACGGTCCGCGAGCGCTACCTCGCGGCCTTCGCGCGCGTCCTGCCCGGCCTTTCCCCGGAGGAG<br>CTGCGGTTCCGGATGCGGGGGATCCTCGCCGTGACGGCCGTCGACCGCCTCGAGGTCCACCAGCAGCCCTCCCCGGGCT<br>GCGCGTCCCCGGCAGCGGACGAGGCGGCCCAGCGATGGGCGATCACGTTCCTGACGGCAGCGATGAGTGCGCCACCGA<br>CCCGGACCTGACGACCCAGGACAGACGCCGGCGGCGGGGGAGCGAGTGGGCCCGGGACGGCGCGTGCGCGTTCCGCG<br>CCCGGGCGCGCGCGTCCATATGCTCGTCCC
SCO6250	CCTCGGTCATGCGGGCCGCGGCCGCCTGCGAGGCCAGGAAGACGCCGCGGACGTTCACCGCCAGCAGCCGGTCGACGT<br>CGGCGGGGGAGAGGCTCTCCAGCGGACCGAGCAGGCCCAGGCCCACGTTGTTGACCAGCACGTCCAGGCCGCCGCCGA<br>GCGCCGTGGCGGCCCGCTCGACCGCCCCCGCCGCCTCGCCCGCGTCGGCGGAGTCGGCCCGCAGGGCGACCGCCCGG<br>CGCCCCAGCGCCCCGACGGCCCGTACGACGTCCTCGGCCGCGTCCTCGCCGTTCACGTAGGTGAGGGCCACGTCCGCG<br>CCCTCCCGGGCCAGCCGCAGGACGGTCGCCGCGCCGATGCCCCGGCTGCCACCGGTCACGAGGGCGGTACGGCCGGCC<br>AGGGGAGCGGTGGTGGGGGCGAGTGGGTTGGTGTGCGTGTTCGTGTTCATGGGTCCATCCCAGCCGGGCCGCCGTACC<br>CGTGCTGGCGGTGAACGGACGTCGACCTGAGCG
SCO6333	GACTGGCCCGCATGATGTCCACGGAGGAGATCGCCGCTGACCTGTACGTGTCGATCAACACGGTGAAGACCCATCTCAAG<br>AGCGCCTACCGGAAGCTGGCGGTCAACCGGCGCGGCGACGCGGTACGCCGGGCGCGCGACCTCGGTCTCCTGTGACCT<br>CCCCCGCGAGGGGTGAGGCACGCGGGGCCACCGCGGGGGTGCGATGGGAGGAACGTGATCCGGCGAGGTGAGACGAG<br>GTGAAGCACCGGCGGGCCTTGGCAGTCCTCGGTACGGCCCAAATCCTGATGGTCTTGGACACCTCCACGATGAACGTGTC<br>GATCAGCCAGTTGGTCGAGGACTTCGACACCCGCGTGTCTTGCGACCGTCGCGCTTGCGAGTTTCCTGGCCACCGCGCGT<br>CTCCCGACCGCCCGGGCAGGCCAACCGCCCGAACCCGAACCTGCGTGCCGGGACCCGGAAAACCGGGCCGACCGGGCC<br>GACGGGCTGAGCACGAAGGAGGCACAG

SCO6344	TGTGACCCGCCAGCCCTTGCCGTCGTCGTCCAGGTCGGCCCACCGGGCGTCTCCGCCACTGCAGTTGCGCAGCCCCTTC<br>GGGCCCTCGACGCTCGCCGGCTCCCACTTGATCTGACCGCACTGCACCACGCGGTTGGCGCACTGGTCCTGTCGGCTCC<br>CCGGCGAGACCACCCAGCCGTGGGCGCTCGCGGTGGGCGCCGGCAGACTCAGGGCCACCACGGGAGCGACCAGCGCTC<br>CCACTGCGGCGGCCACTCGTCTTTTCATGCGCATGACATCACATTTCCCTTCGGTCCACGTCGGATCAGCCTGCCCCGGCT<br>TACGACGGTGCCGATGAGGCAACCCCGCACCGCAGGTCTAGACCTACGTGTGCAGGTCTAGACCTTAGCGAAGGAGTGG<br>GGGGCGTCAAGGGGCCGCACACCATCCCCGGCCCCGCCGTGCGCGACAACTGCCGTGACCGTGGGGCCTCTTGTGCCG<br>GACGGCCCCGTGTAGTGGGGTCGGCC
SCO6369	GGAGGTGACGGTCGGGGCGGTGGAGGCGTGGGCCGTGGCGATGGTGACGCTGTTCAGCTTGTCCGTGAAGACGGTCGT<br>ACCGCCCTTGTCGGTGCGGGCCCAGCCCTCCGGTACCTTCACCGTGAACCCGGTGAAGCCGCCGGTGGGACGGTAGGCC<br>ACGAACACCTGGTTGTCGGGGATGTCACCGGGCGGGTTCGACTCGGTGGGGGCCGGGGTCGCCTGTGCCGGGGCCGTC<br>GAACCCGAGCCGGAATGCGCCCCGGACGATGATCCGGAGCACCCTGCGATCACCGTCGCGACCACCGTCACTGCGACGA<br>GTCCGGCGGAAGTACGTTTGTACGACATGCCATTGCCCTCCCGAGGAACTGCCGGGAACTGTTCGCCCGCCATGCCACCG<br>ACGCTAGAGAGCCGTCGGTTAACGCCCCGCACGCCGACGGTTAGACGACGGCAAAAGCGCGTGGCGGACCGCCCGCGG<br>ACCGGTCGGGGGCCCGCAGGATGGGGGC
SCO6387	ACTCGCCTTGCCTCATGCACTGGTCGAGTGGGTTACCACGCTCATCGTCACCCGAGGGTGACTGGCGCTGCAAGCTCCCG<br>CCGCACCAACGCGCGCTCGTTGCTCTGGTGTACCTACGCAAGCACGACACCCTGGCGCCGGTCGCCGCCGGCTTCGGCA<br>TCTCGGTCGGCACCGCCCACGCCTACACCACCGCCGTCATCGACCTCCTGGTCAGCGATGTTGCCGCGGCCCACCGGGG<br>ATGCTTGTCTGCTGCGGTCCCCGAGCCCGAAGCCGCATCACCGCCAACGGGCCCGGCGGAGCCGTCCGACGAGGCCGAC<br>ACCCCGCCCGCCGCCACGCGAAGCGGGTGTTCGAAGCGGTGTACGCGGTCACCGACACTGGCTGCTCGCTCAACTCGGT<br>AGCCCACGAGTCGGGCCTGAACCGCCGCACCGTGGGAAAGTACGCCCGGGCAGCCACCTGGCAGGAAGGCGTACGCCG<br>GGTTCCGCCTCGTCGGCCTACGAGCC
SCO6390	TCCCCTCGGCTCCCGCGAAGATCACCTCTTCGGACTACCCGACGAAAACCTGGGTCAAGGGCGTGAGTCGGGCGGGCAC<br>CTTCACGCTGACCCGCCCGCATCCCACCACAACTGGCTGGAGTGGTCGCTGGACGGGGTGTCGTGTACCAGGTTGCTACC<br>GGCGGCTCCGCTACGGACCTGAGTGAGCCTCTATCGTCCTCCGCGTTGCTCTGCCGCCGCCGCGGCTTCGCGGCCGCCT<br>CGGCAGGGCCAGCTGAGCACCCCAACGGGATGTCGGCTCTTCCATGGCCGATACCCCTGATGCCAAGCCCGTGCGGGCC<br>ACCCAGCCTGCGGTCTGGCGACACAGCGCCTCCCGGTGTCGGCATTGCGCCCCAGCAGCAGGGGCACCCTCGCCGATTC<br>CGGAGAGTTCTGGGTTCGGAGACACGCGCTTGGACCGGTCCTCTGTAAAGCACAGAGTGGCCGTCCGAACGGCGGCAGA<br>GTAGGAGATCTACGAAAATCACGGC

SCO6460	GCACGTGCTCTTCAACAACTGCTGCGCGGACGCCGCCGTTGGAGCCGCCGAGAGCATGCGGCGGCTGCTCACTCGGCCG<br>TGTCGCTGATCCCCAGCCGCAGGTGCTCCACGTGGTACACGGCCTGGTCGAGCAGCCCGGCCACATGGTCGTCGTGCAG<br>CGCGTACACCACCGAACGGCCACGTCGCTCACCCACGACGAGGCCCAGGTTGCGCAGCAGTCGCAGCTGGTGGGAGCAG<br>GCGGACTGCTCCATGCCGACCTCCGCCGCCAACTCGGTCGCGGGCAGCGGGCCCTCGCGCAGCCGGGCCAGGATCAGC<br>AGCCGGGAGGGGGTGGACAGGGCCTGGAGCGTGGTGGCGACCTTGGTGACGTTGTCCGCGTCCAGCCGCACGCGTGCG<br>GCGGCGTCCTGCGCGGTGGTGGCGGCTCCGTGACCCATGCGGTTCATCCTACTCACCACACATGAACACATGAATGAGTC<br>TTCATCCGTTCCTGTAAGGTGTGCCGC
SCO6513	GTCACCAGCCGCAAACCGGACGACCTCGAGGCGTTCTGCGAGACCTACCTCGCGCAGTTCGCCAAGGCGGCCGGCTGAG<br>ACGGACAGGAACAGCGCGGACGACCGAGCGACGGGCGGACGGGCCACCGGCGAGGGATCGCCGGTGGCCCGCCGTCG<br>CGCTCAGCCACCGGGCGCGGCGGCCTGGCCGCAGCCGCGTTCCCGGGCGCCCTCGCGGGTGCGGCCGGGGGCCACCG<br>GGCGCCGGGGGTTGCGGCGCAGCCACTCGCCCTCCAGCCGGGCCATGCGGTCGTTGTGGGCCCGCAGCGCGTCGTTCG<br>ACCCGTACAGCAGGGTGTCGTGGCGCGTGCGGTGGATGGTCTCCAGCTCTTTCATCAGCTGCTGGTCGTCCAGGCGGCC<br>CGGGTCCACTCCGGTCATGGTGTCGTCCCGCGTGTCGTGTTCGTTCATGCGTCCCGGGTACCCGCCCCGCAGCACTACCA<br>CCCCCGAGCGGCACCCGGGAGGGCAGCCAC
SCO6539	CGCGGCACCAGGCTCAGCACGCCCTCCGCGGCCGCCGCGGCCCGGGGCTCGCCGCCGCGGGAGTCGTCGTAGCGTTCC<br>GCTTCCTTGTCGTAGTCGAGCACACCGTCAGTGTGCCCCGTGGCCGGCGGCCAGGTCCTCCACCCTGCGGGCGAGCTCG<br>ACGTCCTTCTCGGTGACGGCCCCGCCCGCGCTGTGGGTGTGCACCGCGAGGGCGACCGTGTGGTAGCCGAGGGTCAGG<br>TCGGAGTGGTGGTCGAGCTCCTCCTGCACCTGGGCGACGTGGACGACCATCGCGGCCGCCGCGAAGTGCGAGCCGAGC<br>CGGTAGGAGCGGGTGAGACGCCCGGCGTCGAGGGACCAGCCGGGCAGGGTGGCCAGCCGCTCCTCCACCTCCTGCGGC<br>GACAGCGGTTCGGCGGGCATGGGCTGCGCTCCTCTCCTGGGCGGGTTCGCCTCAGCCTGCCACAGCACGGCCGCTCCG<br>GCGCGCCCCGGCGTCGACTACCGTTCCGGGT
SCO6707	GGCGGTGATGATGAGCAGGACCATGAGGAATCCGGGGAAGGGATTGGTGTCGTCGGGATCCGGACTGATCGCGATGGCG<br>ACGGAGAAGACGGCGATCTCCCCGACGACGTACGCACCCGCCAGCCACGGCTTCACCACGCCCTTGACCGCAGCCACCA<br>CGAAGGGAACGGCGGCTGCGAGTCCCAAGGTGACGATCGGGACCAGCGTCCAGACCACCTTGGCCAGGGCCGACGCGT<br>ACATGCGGGGGCGCACTGGCGTAAGCGGTGCGTTGATGTAGGGGCTGGCCACGAGTTCTCCCGCTGTGTCGACGACGTG<br>CAGGAATTGTGGTGCAACGATGCCGGCCTGTCAGGCGATTCGACCGGCCCAGCACGATCGAGTGATCGAGACGGAGTGT<br>CGTCATCGATCAAGCCGCGTGCGGTCAGCCCACCTGCCTGTCGCCGGGATGTATGGGCGTTGCGCCCGACAGGCGGGAT<br>GCCGCCGACGGCGTACAGAATCGGACC

SCO6709

TCGAATCGCCTGACAGGCCGGCATCGTTGCACCACAATTCCTGCACGTCGTCGACACAGCGGGAGAACTCGTGGCCAGCC
CCTACATCAACGCACCGCTTACGCCAGTGCGCCCCCGCATGTACGCGTCGGCCCTGGCCAAGGTGGTCTGGACGCTGGT
CCCGATCGTCACCTTGGGACTCGCAGCCGCCGTTCCCTTCGTGGTGGCTGCGGTCAAGGGCGTGGTGAAGCCGTGGCTG
GCGGGTGCGTACGTCGTCGGGGAGATCGCCGTCTTCTCCGTCGCCATCGCGATCAGTCCGGATCCCGACGACACCAATCC
CTTCCCCGGATTCCTCATGGTCCTGCTCATCATCACCGCCGCCACACACACCGCCCTCCTCGACAACGACCGGATCAGTGT
CGGCAAGTAGGCGCTCCCGGCCCTACGACCGAGGTCTGCGCCCGCTTTCGAGCCGGGCAGCCGATATGCGAACCGAAGG
CGCTGCGCCTACCGTGGCTCGC

SCO6740

GCGCGGTACTCGGCGCCGGAGAAGCGGTAGGGGCCGATGCCGGGGTAGGCGTTCAGCTCCAGGTCGGCGTGTTCGGCG
GTCTGCCAGGCGAAGTCCCGCCAGACGAAGTCGTCGCCGTCCCGCAGGATCACGGCGGTCACCGCGCCGCAGGCCAGG
TCGGCGCACTCGGGGCAGCCGTAGATGACGTACCGGCCGCCGGGCAGGGGCGCGTCGGCCTCCAGGAGCAGGCAGCG
GACCTGCGCGGTGAAGATGGCGGGCGGTACGTCGGAGGCGAGCGGGGAGACGGCGTCCAGATCGGAGAGCCGGAACAG
GAGGGGGCGTCCGTCGACGACGAAGTCCATGAAGTCGCGGTGGACCTGGAAGTCGCCGTCGGCGTGGACTCCGCCGGC
GCGCGTCGCCGGGGCCAGACCGAAGGTCGCGTACTCGGCAGCCATGCGGTGAGTATTCCCTCGGCCGGGCCTCTTGGAG
CACGCCGTCGGGCGTTCGGTTAACGTCCTGGT

SCO6747

GGAGGAGGACCTGGGCCGGGACGTCGCGCTGACCGTCGCCCGGCACCTGGTGGTGTTCCTGCGCCGGCCCGGGAACCA
GGCCCAGTTCAGCGCCCAGCTCGCCGCCCAGACCGCCCGACGCGAGCCGCTGCGGGAGCTGCAGCACTGGATCACCGA
GCACCCCGGCGACGACCTGAGCGTCGAGTCCCTCGCCGCCCGCGCCAGCCTCTCCCCGCGCCACTTCGCCCGCGCCTTC
CAGGCGGAGACCGGCACGACGCCGGGCCGCTACGTCGACCGGGTCCGGCTGGAGCACGCCCGCCGGCTCCTGGAGGAC
ACCGGCGACGGAGTCGAGGAGGTCTCCCGCGCCAGCGGCTACGGCACGCCGGAGGCCATGCGCCGTGCCTTCGTCAAG
GCGCTCGGCACCTCACCGGCGGAGTACCGCCGCCGGTTCCGGCCCGCGCCGGTCTCCTGACGCACCGGGACACCCACC
CGCCGCACCACCGAACCGAAAGAGGCAGCAC

SCO6748

GTGCGGCCGTGGTCGGTCTCCAGGGCGGTCGGGCGGTAGGGGTGGTCGTCCTCCACGGCCACCCGCACCCACTCGGGC
CCGACGGCGACCTCCACGGCGAGCACCGGGGAGAGCACCGCCGCGTGCCGGACCGCGTTCGTGACCAGTTCCGAGACG
ATCAGCAGCAGGGTCTGCGTCACGTCGTCGGAGACCGGCACGCCCTGGCGCACCAGCAGGTCGCGCACGGCGTGCCGG
GCCTGCGGGACCGAGGCGTCCACGGCGGGGGCGGTGAACCGCCAGACGCCTTCGTACGGCAGCGGCCCGGCGGGCCC
GGTCTCGTCCATCGTCCGGTCGCCACCCCTGGGCTCGATTGTCACCACACCCCTGAGTGTTGGTAACGCACCGTGCCGCC
CCCATGGACTGAACACAAGTCAGCATGTATCGAGCGGTTTCTGACCGTTGGCGCATGAGACGCGACCGTTCCTGTCGCGC
CCGCCCGGCCGCTCGAGGCGGAGAGGCCG

SCO6793	GACCGATAAGCCGGATACGCAACCGTTCCGGCATGGCCGCGAAGCAGTCGGACTCGATCAGAAACGGTCACCGTCGGTG<br>ACGGTCTACGGGGAGCCGGAGGTACCCCGTCGCGCGATCAGGGACCGCGCGGCCCGCCGCGCGGCCTCCGGGTCGAAG<br>CGGTCCGCGTGCCGGGTCGTCCGCGTGGCGCCACAGGGGCACGGAGCCTGAGCGGGGCGCAGCAGGCCGGGTGTGGT<br>CCACAGCCAGAGCCCGAACGCCCCCTCGACGTGCCGCAAGTTCCTGCGCCGGGCCGTCCTGTCAGGGCGCGAACCAGGC<br>GGTGGCCGTCACGGCGGCTTCCTCCCGGGAGACGGAGTTGTACCTGCGGGGCGCACATACGTGCACCATGCCCAGGTTC<br>TCGTCGGTCGCGATGCGGCCCCGGCGAGTATCGACGAACCCATTGACGCCCTCACGCCCCCGTGAAACAAGTATATGGAT<br>TTTGTATCTTCGACTCGCTGGGGGCCCG
SCO7254	CGCACGGCACCGCCGAGGCGGTGTGGCAGGCGGTGCGGCCCGTGCTCGAGAAGGAGGCGCGCGGATCGGTCGAGGCG<br>GTGGCCAGGGAACTGGACGCGGCCCGCGGCCGCAAGGACTTCGCGGCCGGGGTCGACGAGCTGTGGGAGAGCGCCCAC<br>ACGGGCCGTGTCCGGCTGCTGGCCGTCGAGGAGAACTTCAAGGTGACGATGCGCGACGACGGCGAGCATCTGCTCCCGG<br>CCGACGACGGCGACCTCGACGCCCGGGAGGACATCGTGGACGAGATCGTCGAACAGTGCCTGGAGACCGGCGCCGACG<br>TCCGTTTCGTGCCGGACGGCGCGCTCGGGGACGTGGACGGCATCGCGGGGGTACTCCGCTACTGACGCCGGCCGCCGC<br>ACCCGCGGACCGGCCCGCCCCCGGCCTCTTGACGCGCGTGAAAGCCTGTTCGCCGTACACGTACCCGCTGCCGGCGGGT<br>GCGCGGCGGACGACGAAGGAGCGGGCGCGGC
SCO7255	CCGACGACCCCGACCGCCCCACCGCCGCCCAGGTCACCGCGTCCCTCGACCGGCTGCGCGCCCGGTACGAGGACGTCT<br>CGCCGGTCTTCGGCCGGTACCGGCTCACCCAGGTGCTCATGTGCTACGGCAGGCCCAGGGGAACGGACTACATCCGGGA<br>CGACGTGAAGGACCTCGACACCGCGAGGATGCTCCTGGTCGGCACCCGCGGCGACCCGGCCACGCCGTACCGCTGGAC<br>GACGGAGACTGCGGACCGGCTCGGCCCCTCCGCCGTGGTCCTGGACAACCGGGGCGAGGGGCACACCGGCTACGCGTC<br>GTCCAAGTGCGTGCACCGCAAAGTCGACGACTTCCTGCTGTACGGCTCCCTGCCGCCCGACGGCAGCTCCTGCGGTCCC<br>GAGTCCACCGGCGACGGGTCCGGCTGAGCGGCGACGGGCCGGACCGAGCCATGGTCCGCCCCGAATGCCCGATATGCC<br>TTATATACGCGTGGAGCATATCGTGCTGAC
SCO7256	CGACGCCCCCGGCTCGCCGGAGTCGGCGCAGCCGTTGCCCGCGGCCGGGTTGAGGAGCCCCACCACGTTCACGGTGTT<br>GCCGCACACGTCGACCGGCACGTCCACCGGGAGCTGCACGGTGTTGCCGGAGATCAGCCCGGGCGAACCGGCCGCGGA<br>TCCCTCGGCGCCGGCCCCGTCGGACGCGAACGCCGCGTACGCCGGCATCGTCACGGCCAGCGCGCCTGAGGCGGCGAC<br>GGCGAGCACACCGTTTCGGGTAACCCTTCTCATGAGGCTCCCTGCCTTCCAGACATGGTCGCGGGCAGTCGCCCGCACCG<br>TCTAAAACGCGGGCGCACCATCCGAGTTATGGCTTATCGGTCTTTCACCCCATCGAGTGGGCGGTTCATCGAACAAGCGG<br>CGCACGGCCCCGTGCGTTGCCGCCCGGAGGCGCCGGGTGCGCGGCCCGCTTATGGTGAGCGAGGGCGCCGTCCACCG<br>GTCCGCGACGGGGCGCCCGGGAGGCATCG

SCO7380	CGGCGAGAGGATCGCCGGCGTTCCCGACCGCCGAGGGGCGCCTCGTCCTCCTCAGCCGGCCGCGTTGTCGGTGCGCAG<br>CCTCACCTGTTCCTCCAGCCGCCGCAGGCTGCCGTCGAGGGCCTCGGCGACCGTCCGCCGGCCCGGGTCGTGGGTGTC<br>GTCGAAGAACGACAGGTGCACCGTGACCTCGCTGGCCCCGCTGTCGAGCCCGGCGACCTGCAGCCAGCCGGCGTAGCTG<br>CCCTGTTCGCGGGTGCCCCACTCGAGCCGCAGCTGCTCGGGCCGGGCGCTCAGCAGCGCCGAGGTGTCCTGGTCGGTG<br>CGGTCCTCGTGCACGGTGACGGCGGGCGGTTCCTCCGGCTCCACGTGCAGGTCCCGGGGCAGCCAGGTCGCCAGCTGT<br>CCCACGTTCGCGGCCTGGTCGAAGACCTGCTCGGGCTGGGCGGGCATGGTGCGGGAACGCTCGTACTCGGTCATGCGGC<br>ACCACTCCTCGGCGCTCGGGGTCGTACGAG
SCO7389	CTACACCCACGTCCCGCACGACATCCGCGCCGACGCGGGCGAGGAGGGCATCACCGGCACCTGGGACACCGGGGAGCG<br>GGAACTGATGGCCGACCGGGTCGAACGCCAGGTGGAACGCTTCGCGCCCGGCTTCCGGGCTCTGATACGGGCCCGCCG<br>CGTGCTCGCCCCGCCGACGCTCCAGGCCCTGGACGCCAACCTGGAGGGTGGTGCCATCAACGGCGGCACCACCGCCATG<br>CACCAGCAACTCGTCTTCCGGCCCGTGCCCGGCACCGGGCGGCCCGAGACCCCGGTGGCCGGTCTCTTCCTCGCCTCCG<br>CGGGCGCCCACCCCGGGGGCGGCGTGCACGGGGCGCCCGGTGCCAACGCCGCGCGCGCCGCACTGCGCCAGCACCGG<br>TTCGCGGGCCTCGCCCGGATCCAGCGGGCCCTGACCCGGCGGGACCGGGCGGGTGACCGGCACTGACCGTCGCGACCG<br>TACGCCAAGTACCCGAGGAAAGGGCCGACCG
SCO7394	ACGTCGGCCCTGGTCTCGCCGGCGCCGATGGTGCCCTGGCAGTTCTTCTCCTGATCGACCGAGACGTCGATGGTGACGG<br>TGCTGCCGTCCTCCAACCGGGTGTCGCCCTTCACGTGCACGGACTTCGCCTGCCGGGTGGCCTCGACCGCCTTCGCGGC<br>GATGTCGTCGGCGCTCTGCCCCTCGAACGGCTTGCCCTCGTCACCCGAATCGCCGCCGCAGCCGGCCAGTAGGGCGACG<br>GACACGCTCGCGGTCGCCACAGCGGTTGCCACTTTGCCGGTACGCACTGGAAAGTCCTCCCCAAGTCTGTTCATAACCGT<br>CAAATCCCTTGCTGTCCCGATGTGTTGGCATCGGAACCCTTCGACTACCCGCGATCGACGGGACATGCACGTGATGGAGA<br>ATCCGTGACGGGCGGGTGAGCGGTCCCCGGTGTGGATTGGTCCCCGACAGCGCACGCGAAGTGGACCCCGTGACGGGC<br>CGCCACGCTCTCTAGCGTCGTGGCC
SCO7434	CCCACCGTCGACGGCGAATGGGACGGCGTGCACCGCATGCTGCGCGCCGTCGGCCTCAACCCCCGGGTGCTGCACGGC<br>GACTACCACACGGCCGCCTCCCTGGTCGCCACCGGCGAGGTCGTCACGGTCTGCCAGCCCAGTTCCCAGTCCCGCCCCG<br>ACACGGCGGTGCGGCGCCTGTACGGCGACCCCCTCGGCGTACGCCTGCTGCTGGCCGCCCGCACCCGGGCCGAGCTGG<br>ACGCCGTCTTCCCCGCCCTGGAGGACGCGTACTGGGAGGCCGCCCGCCAGTCCACCGCGTACCGGGAGTGGCTGGAAG<br>GGGGCGGGATCCGGACGCTGCCCCGGTGCCCGGTGGCCGCCACGGGAGGCGGCCGGGTGGAGTTCGTGCGGGCGCGC<br>TGAAGGCTCCCGGTCCCGCCGCCCACGGGACGTCGCCCCACCCCGGCCAATAGGGTGATTTTCATACCGGTCCGGTGTG<br>TCTGCGTGACGGAGCGGTTACGGACAGCATC

SCO7450	CACGACGAGCCCCGTGGCGGCATGCACACCGGCGGCGGCGCGCTGGCCGCGGTGAACGAGGGCGACTGGGACGGCGG<br>CGGCCGCGAGTCCAAGGAGGGCGGCTCGCAGACCTACCAGCAGGACGAGGGCGAGAGCTCCAGGGGCGGCGGTGAGCA<br>CGAGAAGCCGCGCGGCGGGATGCACACCGGCGGTGGCGGACTTGCCACGCCGACCACGACCGCGGGCGGGCTCGCCG<br>TCCTGGCCGTCGCCGCGACCGGCCTGTACGCCGTCCGACGCAAGAAGTCGGCTCACGGAGTGGCGTAAGCCGTGCCGG<br>GCGCCATAGCAGCCGGGCCGCCACCCAGGAACCACGGGGCGGCCCGGCTCGCCCCCGCCCACCCCGCCGCCCCTCGTG<br>TCCGTCCTGCCGCCGTGTCCCAGTGAGGTCGTCCCCGATGTCAGTCACCCCTCCCACCCCCGCCCCGGCCGACGACGAG<br>ACGACGACCGGTCAGGGCTCCCGCTCCGGCCTG
SCO7451	GGCCGGCGACACCAACCTCGTCGGCTGGTACGCCAAGGGCGTCTCGCCCGGCGAGAAGGGCACCTCGATCATCGCCGG<br>GCACGTGGACACCAAGACGTCGGCCGCCGTCTTCGCCCGCCTCGACCAGCTCGACAAGGGCGACAAGTTCCAGGTCCGG<br>CGCGCGGACGGGCGCAGCGCCACCTTCGTGGTCGACGGCCTGGAGACCTTCGCCAAGGACGAGTTCCCGAGCGACCGC<br>GTCTACGGCGACGCCGACCGGCCCGAGGTGCGACTGATCACCTGCGCGGGCGACTACGACCACAAGGTCAAGGACTACA<br>CGGACAACCTGGTCGTCTTCGCGCACCTCGCCTGAGGCCGCGCACCGGGCCGGCCGCGGCGCCGGTCACCCGCGCACC<br>GCACACGGGTCGCGCGACGGCGGGGCCCGGCACAGGATCGAGGCAAGCCGGCGTCGCATCACCGCGGCCGACGTTCAG<br>TTCCGCCCCCGCGAAGGAGATGTGCGCAGG
