# Supplementary material for: Genome-wide identification and evaluation of constitutive promoters in streptomycetes
Source: Microb Cell Fact. 2015 Oct 29;14:172. doi: 10.1186/s12934-015-0351-0 (PMC4625935; doi:10.1186/s12934-015-0351-0)
Supplement: Supplementary file 6 — 10.1186/s12934-015-0351-0 Growth curves of S. coelicolor M1146, S. venezuelae WVR2006, and S. albus J1074. [file 12934_2015_351_MOESM6_ESM.pdf]

Additional file 6: Figure S1

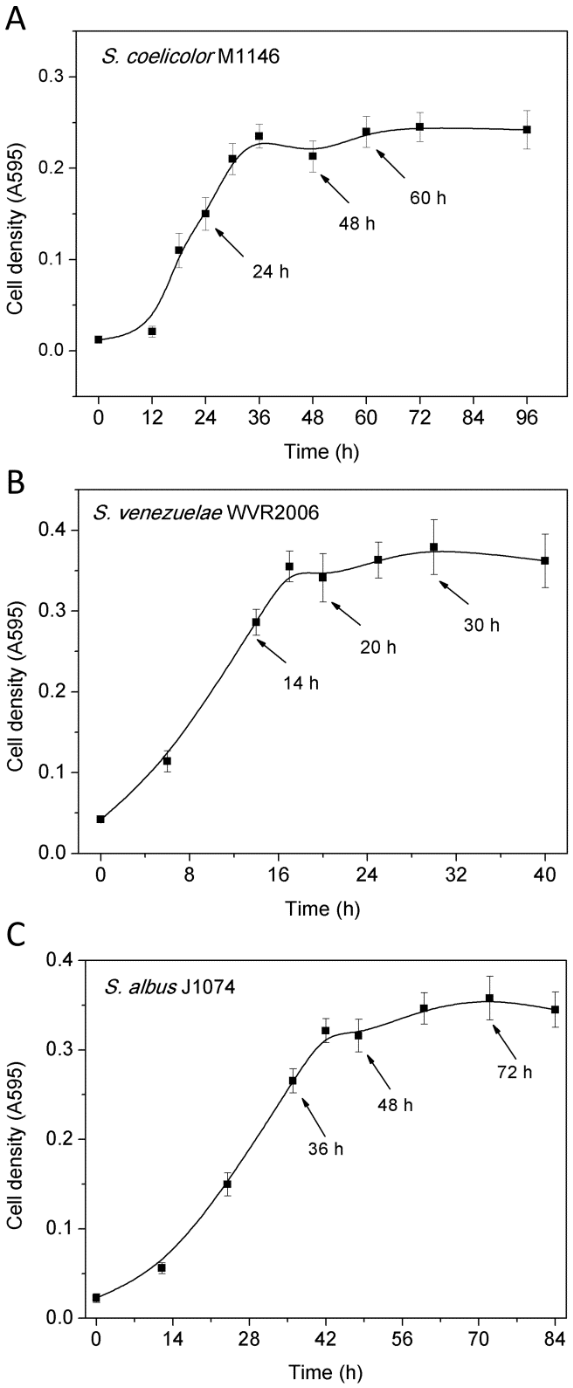

Figure S1 Growth curves of *S. coelicolor* M1146, *S. venezuelae* WVR2006, and *S. albus* J1074.

Cell growth was monitored by using the diphenylamine colorimetric method[1]. For growth quantification, cell pellets were harvested from 1 ml cultures by centrifugation

at 10,000 × *g* at room temperature for 10 min and washed twice with the appropriate buffer. The cell pellets were then resuspended with 2 ml diphenylamine reagent and incubated at 60 ℃ for 1 h. After centrifugation, the supernatants were transferred to 96-well microtiter plate and measured at 595 nm by a multifunctional microtiter plate reader (Synergy Hybrid Reader, BioTek, USA).

Reference

1. Zhao Y, Xiang S, Dai X, Yang K. A simplified diphenylamine colorimetric method for growth quantification. Appl Microbiol Biotechnol. 2013;97:5069-77.
