# Supplementary material for: Genome-wide identification and evaluation of constitutive promoters in streptomycetes
Source: Microb Cell Fact. 2015 Oct 29;14:172. doi: 10.1186/s12934-015-0351-0 (PMC4625935; doi:10.1186/s12934-015-0351-0)
Supplement: Supplementary file 7 — 10.1186/s12934-015-0351-0 Primers used in this work. [file 12934_2015_351_MOESM7_ESM.pdf]

Table S3 Primers used in this work

Primer name	Sequence (5'–3')
**For strain construction**	
ermEF	CGACAAAACTTTAGATCCTCGAGATCTGATATCGGAGACCAGAGCGAGTGTCCGTTCGAGTG
ermER	AGCTCTTCGCCTTTACGCATGAGACCATCCTACCAACCGGCACGGTTGTG
4503F	ACAAAACTTTAGATCCTCGAGATCTGATATCCCTCGCCGAGCGGGAGG
4503R	AGTGAACAGCTCTTCGCCTTTACGCATGGGCGGCTCCTGGGACGTG
4508F	ACTTTAGATCCTCGAGATCTGATATCAGGCCGTCGAGGGTGTCCTTG
4508R	AGTGAACAGCTCTTCGCCTTTACGCATGCTTGTCCTCCCCAGGCGAG
6740F	ACTTTAGATCCTCGAGATCTGATATCGCGCGGTACTCGGCGCCGGAG
6740R	AGTGAACAGCTCTTCGCCTTTACGCATACCAGGACGTTAACCGAAC
3410F	ACTTTAGATCCTCGAGATCTGATATCCGCCTGGACCGCCGCCTGTT
3410R	AGTGAACAGCTCTTCGCCTTTACGCATTCCCTCCTGTTGACCTCAGTA
4658F	ACTTTAGATCCTCGAGATCTGATATCGCGCCGGGTAGAGCGGGAAG
4658R	AGTGAACAGCTCTTCGCCTTTACGCATCGCGCCTCCCTCCTCATGACCTTG
2468F	ACTTTAGATCCTCGAGATCTGATATCCGCGCGCACGGCGGCGGCATC
2468R	AGTGAACAGCTCTTCGCCTTTACGCATGCGTGAAGTCTACGGGGGCGGAC
2074F	ACTTTAGATCCTCGAGATCTGATATCGCTGTTCCCAAGGCCCGCGGCACC
2074R	AGTGAACAGCTCTTCGCCTTTACGCATGTGAGTCCCTCAGCCTAGGTC
5768F	ACTTTAGATCCTCGAGATCTGATATCCCCGAGGACTCGGGGCCGTAG
5768R	AGTGAACAGCTCTTCGCCTTTACGCATGTGCGTCCCCTCTCCGACCT
gfp-cx-R	AGAGGTACGGGCTGCAGCCGGGCCTGGCTCATCATTTGTACAG
LNF	CCGTACTGACGGACACACCGAACGCCAAAGACCACCGGAAGGGAC
LNR	GTCCCTTCCGGTGGTCTTTGGCGTTCGGTGTGTCCGTCAGTACGG
IJF	ATGCGTAAAGGCGAAGAGCTGTTC
IJR	CGATATCAGATCTCGAGGATCTAAAG
LN0F	GCCAGGCCCGGCTGCAGCCCGTAC
LN0R	GAGACCATCCTACCAACCGGCACGG
JF	CCGTGCCGGTTGGTAGGATGGTCTCATGCGCAAGGTGCTCATCGCCAAC
JR	GTACGGGCTGCAGCCGGGCCTGGCCGTCCGCGATCCGGAACATCTCCC
6740R1	TGGCGATGAGCACCTTGCGCATGAGACCACCAGGACGTTAACCGAACGCCCGAC
5768R1	TGGCGATGAGCACCTTGCGCATGAGACCGTGCGTCCCCTCTCCGACCTCTC

2074R1	TGGCGATGAGCACCTTGCGCATGAGACCGTGAGTCCCTCAGCCTAGG TCCTTGAC
2468R1	TGGCGATGAGCACCTTGCGCATGAGACCGCGTGAAGTCTACGGGGGC GGACCGAC
**For real-time RT-qPCR**	
gfp-F	GGGTGAAGGTGACGCAACTAATG
gfp-R	CGGATAACGAGCAAAGCACTGA
hrdB-F-sc	GAGGACGGCGACAGCGAGTT
hrdB-R-sc	GACGCCGTACACCTTGCCGA
hrdB-F-sv	GCCGAGTCCGAGTCTGTGA
hrdB-R-sv	CTGGGTTGGCGGAATCTGGT
hrdB-F-sa	AGGCCCGCACCATCCGTATC
hrdB-R-sa	GGGTCATGTCGAGTTCCTTGGC
